# Clinical, cytogenetic and molecular genetic characterization of a tandem fusion translocation in a male Holstein cattle with congenital hypospadias and a ventricular septal defect

**DOI:** 10.1371/journal.pone.0227117

**Published:** 2020-01-10

**Authors:** Alessandra Iannuzzi, Marina Braun, Viviana Genualdo, Angela Perucatti, Sina Reinartz, Ioannis Proios, Maike Heppelmann, Jürgen Rehage, Kirsten Hülskötter, Andreas Beineke, Julia Metzger, Ottmar Distl

**Affiliations:** 1 Institute for the Animal Production System in Mediterranean Environment (ISPAAM), National Research Council (CNR), Naples, Italy; 2 Institute for Animal Breeding and Genetics, University of Veterinary Medicine Hannover, Hannover, Germany; 3 Clinic for Cattle, University of Veterinary Medicine Hannover, Hannover, Germany; 4 Department of Pathology, University of Veterinary Medicine Hannover, Hannover, Germany; University of Connecticut, UNITED STATES

## Abstract

Hypospadias, disorder of sex development (DSD), is a sporadic congenital abnormality of the genital region in male ruminants, which is characterized by a non-fused urethra during fetal development. Detailed clinical examination classified the hypospadias phenotype of a male Holstein calf studied here as the perineal type. In combined use of cytogenetic analysis and whole genome sequencing, a non-mosaic, pseudo-monosomy 59, XY + tan(18;27) was detected. This chromosomal aberration had its origin in a tandem fusion translocation of the bovine autosomes (BTA) 18 and 27 with an accompanying loss of genomic sequences mainly in the distal end of BTA 18 and the proximal end of BTA 27. The resulting phenotype included hypospadias, growth retardation and ventricular septal defect.

## Introduction

**Fig 1:**
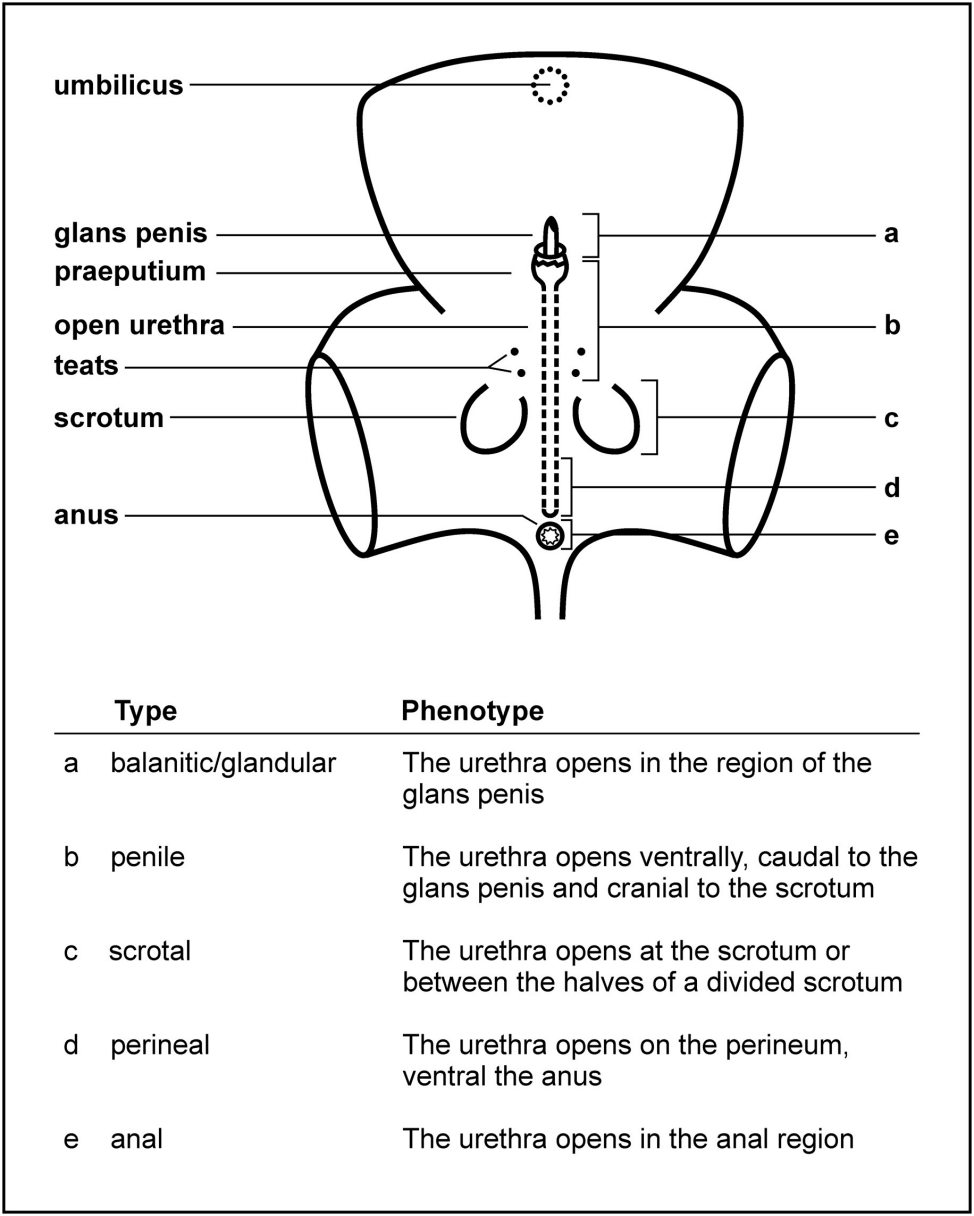
Classification of hypospadias phenotypes. Visual classification of different types of hypospadias modified from Smith, Brown [12]. The phenotypes of the different types (a–e) are shown below.

Hypospadias (OMIM 300758), an important congenital genital abnormality in humans [1] often associated with mental retardation [2], is characterized by a pathological opening of the urethra where the urethral meatus opens on the gland penis in about 50–75% of all cases (first degree hypospadias). It is after cryptorchidism the second most common human birth defect of the male genitalia, detected once every 330 male births [3] and up to now in humans, only three nonsense mutations within the *mastermind-like domain containing 1* (*MAMLD1*) gene have been correlated to it [4]. Further genes were supposed to be associated with hypospadias in humans [5]. It sporadically also occurs in male carnivores [6, 7], horses [8–11], sheep [12], goats [13], water buffaloes [14], cattle [15] (Table 1) and dogs [9, 16–18]. The characteristic phenotype observed in all these mammals is a result of a partial or complete failure of formation or fusion of the two urethral folds in the midline during phallus elongation [19, 20]. As a consequence, the urethral groove, which is formed by invagination of the urethral plate, and physiologically closes to form the tubular extrapelvic urethra in fetal development, remains open [12]. The fusion process begins proximally in the perineal region and extends distally toward the glans penis at the solid urethral plate [21]. It is accompanied by the failed development of the median raphe of the perineum, scrotum, penis (penile aplasia or hypoplasia, especially of the corpus cavernosum urethra) and prepuce [22, 23]. The scrotum can be partly or completely divided, which is particularly clear in the pendulous scrotum of ruminants [12, 24]. The site of fusion failure dictates the position of the abnormal opening of the urethra. It can be located in anal to distal positions along the shaft of the penis [25]. Depending on the manifestation of the urethral opening, hypospadias is classified into different types: balanitic/glandular, penile, scrotal, perineal and anal (Fig 1) [26]. In the perineal form, where the urethra is completely open, the urethral orifice appears to resemble a vulva [27]. If the animal also has a scrotum and penis, it may be mistaken for hermaphrodite or pseudohermaphrodite [23]. Cases of female calves, non-hermaphrodite or non-pseudohermaphrodite, with hypospadias in cattle are very rare [28, 29].

**Table 1:** Overview of different types of hypospadias in ruminants. Species, breed, number of cases, sex and phenotype are given.

Species	Breed Number of cases	Sex	Phenotype of the genital region	Other abnormalities	Reference
Cattle	Bunaji n = 2	Male Freemartinism	Urethra opened ventral posterior to the scrotum Bifurcated scrotum No preputial orifice	Atresia ani Imperforated left ear	Kumi-Diaka and Osori [15]
	Jersey crossbreed n = 1	Male	Complete absence of a preputial orifice	Atresia ani	Misk and Rilat [30]
	Indigenous Korean breed n = 3	Male	Aplasia of the penis along with an undescended testis, bifid scrotum and ventrally incomplete sheath (penile aplasia and unilateral cryptorchidism) Urethra opened cranially to the bifid scrotum or at the ventral perineum	-	Alam, Shin [23]
	Japanese Black, Holstein x Japanese Black, Jersey n = 18	Male	Urethra opened in the perineal (n = 18), periscrotal (n = 2) and glandular (n = 1) region	Anorectal anomalies (n = 9) Cardiovascular anomalies (n = 8) Cryptorchidism (n = 7)	Murakami [31]
	Indigenous Korean breed n = 3		Urethra opened in the perineal region Bifid scrotum Aplasia of the penis Incomplete sheath	-	Jeong, Seong [32]
	Simmental n = 1	Male	Urethra opened in the perineal region Penile and preputial aplasia	Rudimentary left kidney and left urethral anomaly	Pamuk, Korkmaz [33]
	Hereford n = 1	Male	Urethra opened in the glandular region of the penis	-	Vidal, Traslavina [34]
	Indigenous Korean breed n = 1	Male	Urethra opened in the perineal region	-	Park, Yang [35]
	Holstein-Friesians, local breeds n = 3	Male	-	-	Misk, Misk [14]
	Crossbreed n = 1	Female pseudo-hermaphroditism	A rudimentary urethral orifice below the anus Urethral diverticulum with a preputial opening	-	Abd-El-Hady and El-Din [28]
	Red Holstein n = 1	Male	Urethra opened in the perineal region Penile aplasia Bifid scrotum	-	Mihsler, Hussein [36]
	Boran n = 1	Male	Urethra opened in the ventral perineal region instead of the glans penis Penile aplasia Bifid scrotum	Atresia ani	Phiri, Sakala [27]
	- n = 1	Male	Bifid scrotum with bilaterally descended testes, incomplete prepucial sheath with penile urethral opening	Atresia ani	Potliya, Chaudhary [37]
	Nelore n = 1	Male	Urethra opened in the scrotal, anal and perineal region Bifid scrotum	Atresia ani and recti Omphalophlebitis	Torres, Lhamas [38]
	Holstein n = 2	Male	Urethra opened in the perineum (n = 1) Urethra opened in the ventral perineal region (n = 1) Penile and preputial aplasia	Atresia ani	Usta and Distl [24]
	Crossbreed n = 1	Female pseudo-hermaphroditism	Urethral diverticulum Hypoplastic penis with adhesion of the preputeal sheath along with penile hypospadias Rudimentary scrotum	-	Maiti, Raghuvanshi [29]
Buffalo	Bubalus bubalis n = 3	Male	-	-	Misk, Misk [14]
	Mediterranean breed n = 1	-	-	-	Volpato, Araujo [39]
Sheep	New Zealand Romney n = 16	Male	Urethra opened in a periscrotal position or close to the anus Scrotum was completely or partly divided	-	Smith, Brown [12]
	Suffolk, Texel, Down, Blue Face Leicester, Hill or Mountain sheep n = 15	Male	Urethra opened in a periscrotal position, close to the anus or close to the anal sphincter (bore a superficial resemblance to a vulva) A complete or incomplete divided scrotum Ulceration of the scrotal skin Penile aplasia The galea faced caudally and was fused to the preputial mucosa The penile shaft was tortuous and lacked a sigmoid flexure The vermiform appendage was absent (n = 13)	-	Smith, Brown [40]
	Rahmany, Barki, local breeds n = 2	-	-	-	Misk, Misk [14]
Goats	- n = 1	Male	Urethra opened in the scrotal region or penile position Penile and testicular hypoplasia Ectopic penis	-	Sakhaee and Azari [13]
	- n = 1	Male	Urethra opened below the anus Bifid scrotum with descended testis and ventrally incomplete sheath Aplasia of the penis	Absence of the tail Atresia ani	Veena, Sankar [41]
	Capra hircus n = 1	Male	Urethra open in the penile region Urinary tract was absent in the anterior part of the penile urethra Penile diverticulum	-	Omidi, Monjezi [42]
	- n = 2	Male	Urethra open in the penile region Two urethral diverticula caudal to the tip of the penis Testicular and penile hypoplasia	-	Bokhari [43]
	Baladi, Zarabi, local breeds n = 9	Male	-	-	Misk, Misk [14]
	Saanen n = 1	Male	Urethra open in the penile region Prescrotal diverticulum	-	Almubarak, Abdelghafar [44]

The aim of this study was to investigate a male Holstein calf with congenital hypospadias and ventricular septal defect (VSD) using cytogenetic analysis as well as whole genome sequencing data. To contribute to the understanding of this birth defect, the phenotype was classified according to the different types of hypospadias.

To our knowledge, we show the first report of an autosomal aberration associated to a phenotype of perineal hypospadias, VSD and growth retardation in Holstein cattle using the technical-laboratory combination of cytological and cytogenetic examinations, as well as whole genome sequencing.

## Results

### Clinical examination

**Fig 2:**
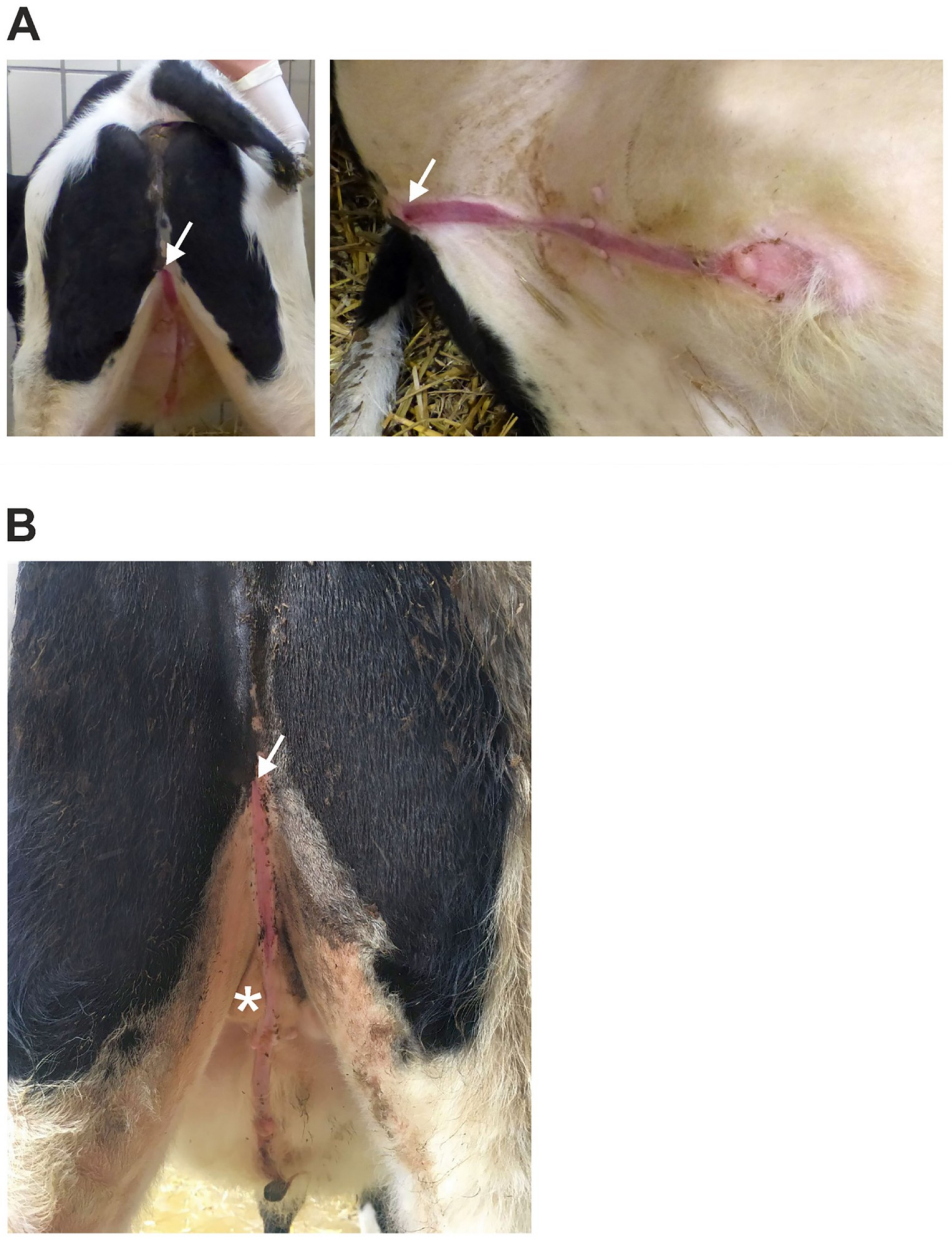
Hypospadias phenotype of the Holstein calf. (**A**) The Holstein calf showed a perineal hypospadias. The urethra (marked with an arrow) opened about 20 cm ventrally the anus. An opened sagittal slit run through a divided scrotum and ended at the prepuce. (**B**) At the age of ten months the testicles were fully descended into the scrotum beside the opened urethra (marked with an asterisk).

The affected calf showed a perineal hypospadias, with an opening of the urethra 20 cm (7.87 inch) ventral the anus. As a mainly complete opened sagittal slit, the urethra divided the scrotum and ended at the rudimentary prepuce. The mucous membrane of the urethra was clean, shiny and soft pink. Additionally, an incomplete penile aplasia was identified. Two palpable testicles were descended incompletely in the not fully formed scrotum. In the region of the scrotum the urethra showed a partly closure. The urination was controlled and rhythmical without signs of pain. The skin around the open urethra showed no indications of inflammation or irritations. No vulva was detected and the anus was open and contracted physiologically (Fig 2A). At the age of ten months, the scrotum was visible (Fig 2B).

**Fig 3:**
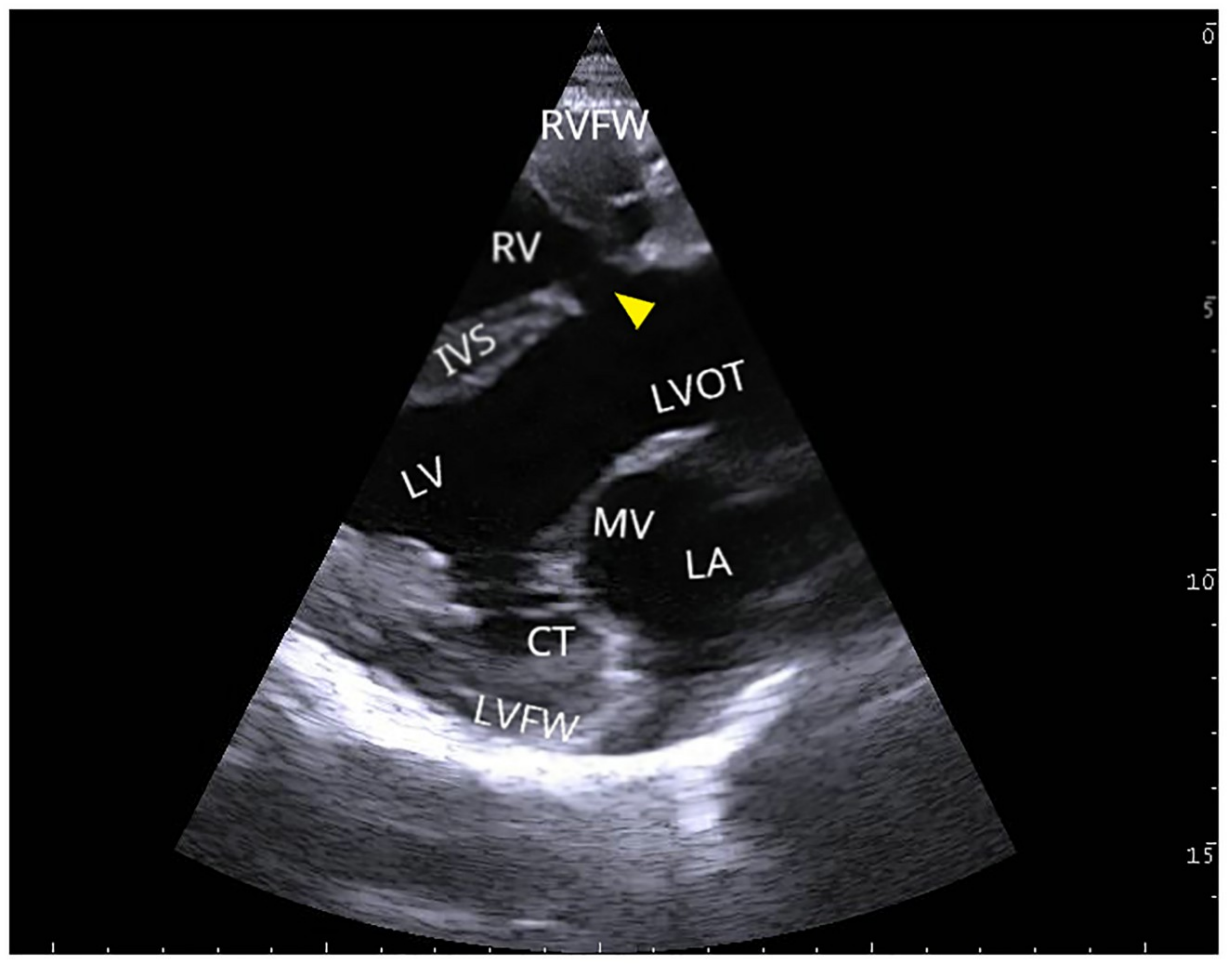
Ultrasound scan of the heart from the right side in the caudal long axle. Between the right ventricle (RV) and the left ventricle (LV), the ventricular septal defect (marked with an arrow) is visualized in the interventricular septum (IVS). The mitral valve (MV) with its *chordae tendineae* (CT), the left atrium (LA), the right and left free wall (RVFW, LVFW) and the left ventricle outflow tract (LVOT) were identified for orientation.

The auscultation of the heart revealed a heart murmur and heartbeats of high holosystolic intensity which were louder on the right than on the left side. In the fourth intercostal space, the ultrasound scan visualized a ventricular septal defect (VSD) of few centimeters underneath the aortic valve (Fig 3). The cardiac valves themselves showed no defects. The calf had a body weight of 68 kg when delivered at our clinic with an age of approximately 4 months. In comparison to normal male Holsteins, growth rate was reduced because a body weight between 120 and 140 kg should be expected at this age. Laboratory blood values were in the physiological range. The sire and the dam of the calf showed no signs of genital or urogenital abnormalities. This case occurred isolated within the herd for the first time according to the report of the farmer.

Over the two years the animal was observed, the scrotum with the testicles of the male calf were further on visible but descended incompletely. A notable growth retardation compared to animals of the same age was obvious after the two years observation period. The weight range of male black and white Holsteins with an age of 24–30 months in our clinic was at 550–650 kg. The animal investigated here had a weight of 292 kg with an age of 29 months. The head was proportional in size to the smaller body with a slightly broader forehead combined with a moderate narrow muzzle. The hind limbs showed severe soft pasterns which evolved to a mild lameness over time.

### Necropsy

The animal examined was in good body condition with a weight of 292 kg. The urethra was ventrally open starting about 20 cm (7.87 inch) ventral at the anus. The open sagittal slit run 44 cm (17.32 inch) to the prepuce. Parts of the rudimentary glans penis 1.5 x 2 x 1.5 cm (0.59 x 0.79 x 0.59 inch) stuck out the prepuce. The testicles had a size of 6 x 3.5 x 2.5 cm (2.36 x 1.38 x 0.98 inch) and were located outside the groin in the not completely developed scrotum.

The VSD had a diameter of 2 cm (0.79 inch). A subendocardial edema was seen in the right ventricle.

Histological examination of the distal part of the open urethra displayed metaplastic, cutaneous mucous membrane with focal surface erosions. The part of the urethra which was located inside the body revealed no abnormalities.

No histologically detectable abnormalities were identified in the part of the rudimentary glans penis. The testicles showed inactive germinal epithelium and focal hypoplasia of the Leydig cells. Additional multifocal hypoplasia of the Sertoli cells was seen. The epididymis had no storage of sperm.

### Cytogenetic analyses

**Fig 4:**
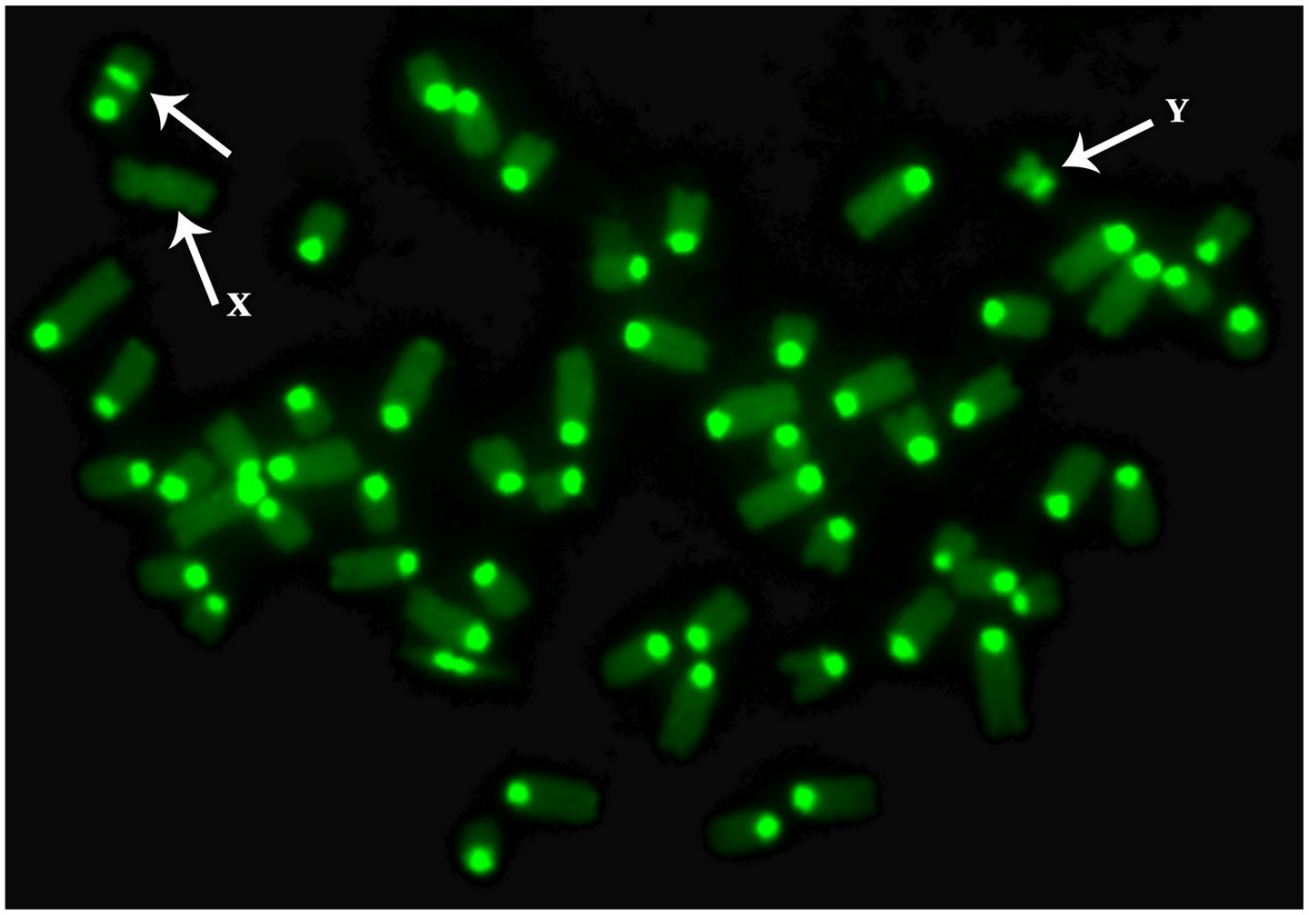
CBA-banded metaphase spreads of chromosomes. CBA-banded metaphase spread showing a chromosome (arrow) characterized by two constitutive heterochromatin blocks (C-band positive-centromeric and median position). Sex chromosomes are also indicated.

**Fig 5:**
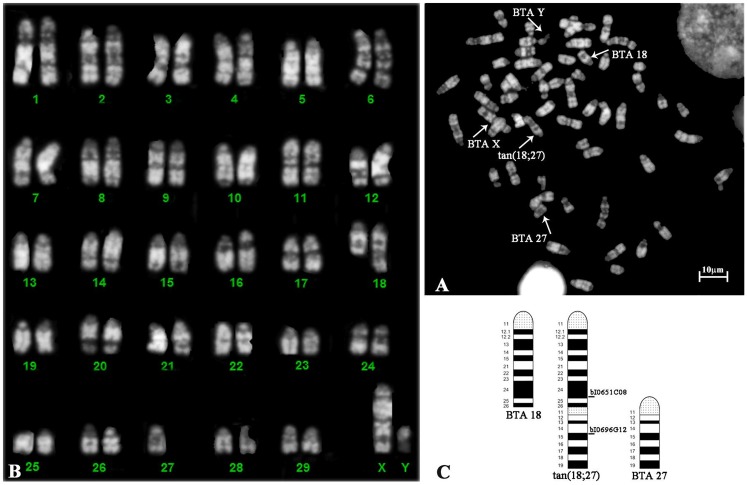
RBA-stained metaphase spread with corresponding karyotype and ideogram of bovine chromosomes 18 and 27. RBA-stained metaphase plate (**A**) and the corresponding karyotype (**B**) showing BTA 18, 27 and tan(18;27) involved chromosomes. (**C**) Ideogrammatic representation of the BTA chromosomes involved in the tandem fusion translocation and the localization of BACs used for FISH analysis.

The animal presented 2n = 59, XY chromosomes in all 150 conventional metaphase spreads counted with normal X and Y sex chromosomes. CBA banding revealed in all metaphase spreads two constitutive heterochromatin blocks (C-band positive-centromeric and median position) in an acrocentric chromosome (Fig 4) indicative of an extra centromere on it. In this way, a tandem fusion translocation (telomere-centromere) between two acrocentric autosomes was hypothesized. RBA-banding (Fig 5A) and the analysis of the corresponding karyotype (Fig 5B), on ten different metaphase spreads demonstrated that BTA 18 and BTA 27 chromosomes were involved in the chromosomal aberration classified as 59, XY + tan(18;27).

### FISH mapping experiments

**Fig 6:**
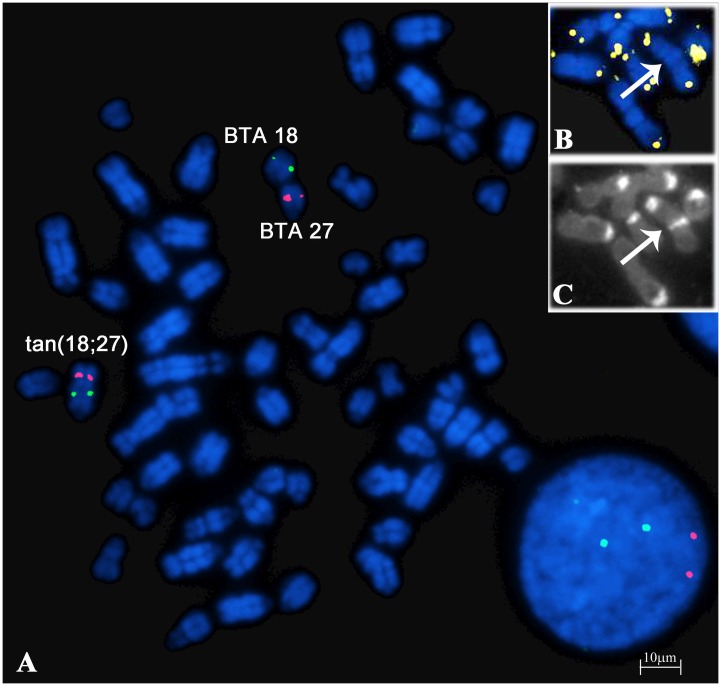
FISH on R-banded metaphase spreads with BAC probes and telomere FISH and CBA staining. (**A**) FISH on R-banded metaphase spreads of the carrier using INRA bovine BAC specific probes. BAC 651C08 hybridizes on both normal BTA 18 and the proximal part of abnormal tan(18;27) chromosomes (green signals); BAC 696G12 hybridizes on both normal BTA 27 and the distal part of abnormal tan(18;27) chromosomes (red signals). Abnormal tan(18;27) chromosome (arrows) after sequential telomere FISH and CBA staining techniques where telomeric signals (yellow) are present only in the distal ends of the autosome (**B**), while centromeric and median positive C-band (**C**) identified it.

FISH analysis using specific BAC probes further confirmed the data, mapping on both normal BTA 18 and 27 chromosomes and the abnormal autosome, as shown in Fig 6A. In addition, sequential telomere and C-banding techniques revealed the presence of the telomeres only in the distal ends of the abnormal autosome (Fig 6B and 6C), assuming that the distal region of BTA 18 chromosome has been lost in the aberration.

### Y chromosome detection

**Fig 7:**
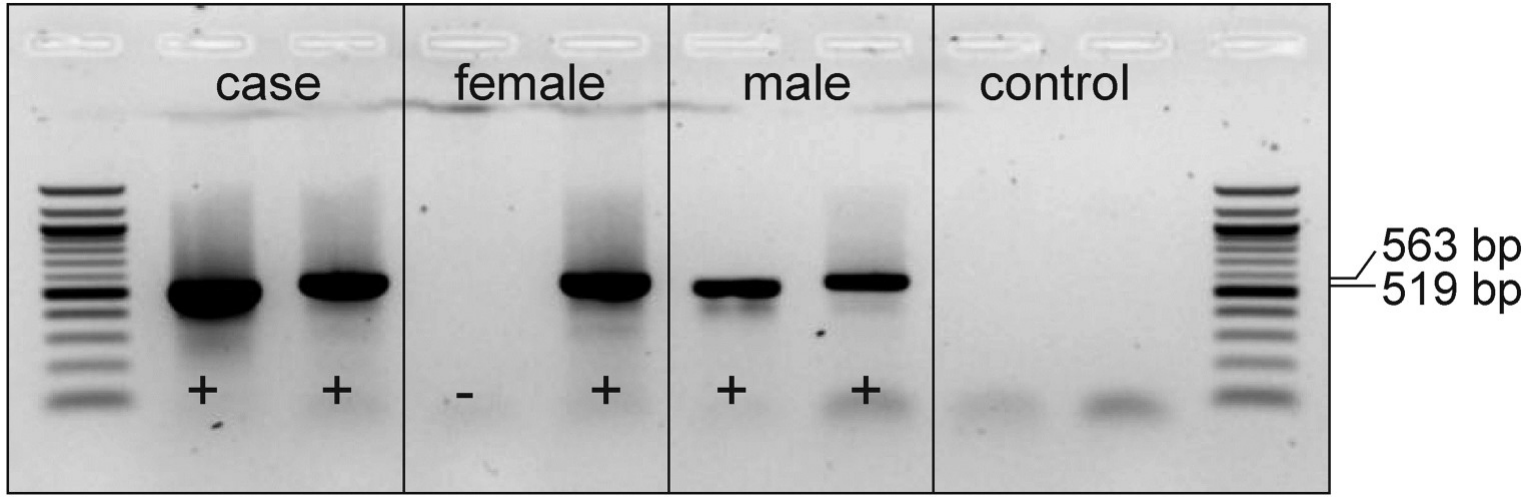
SRY-PCR. PCR-product of the 519 bp of the segment of the *SRY*-gene (left column in each section) confirmed the existence of the male specific Y sex chromosome in the examined case with hypospadias. One female and one male control Holstein cattle validated the result. The verification of the 563 bp amplicon of the *GON4L* gene (right column in each section) indicates the capability of the isolated DNA of the used samples.

The visualized PCR-product of 519 bp of the segment of the *sex determining region Y* (*SRY*) gene confirmed the existence of the male specific Y sex chromosome (Fig 7).

### Validation by structural variant detection

**Fig 8:**
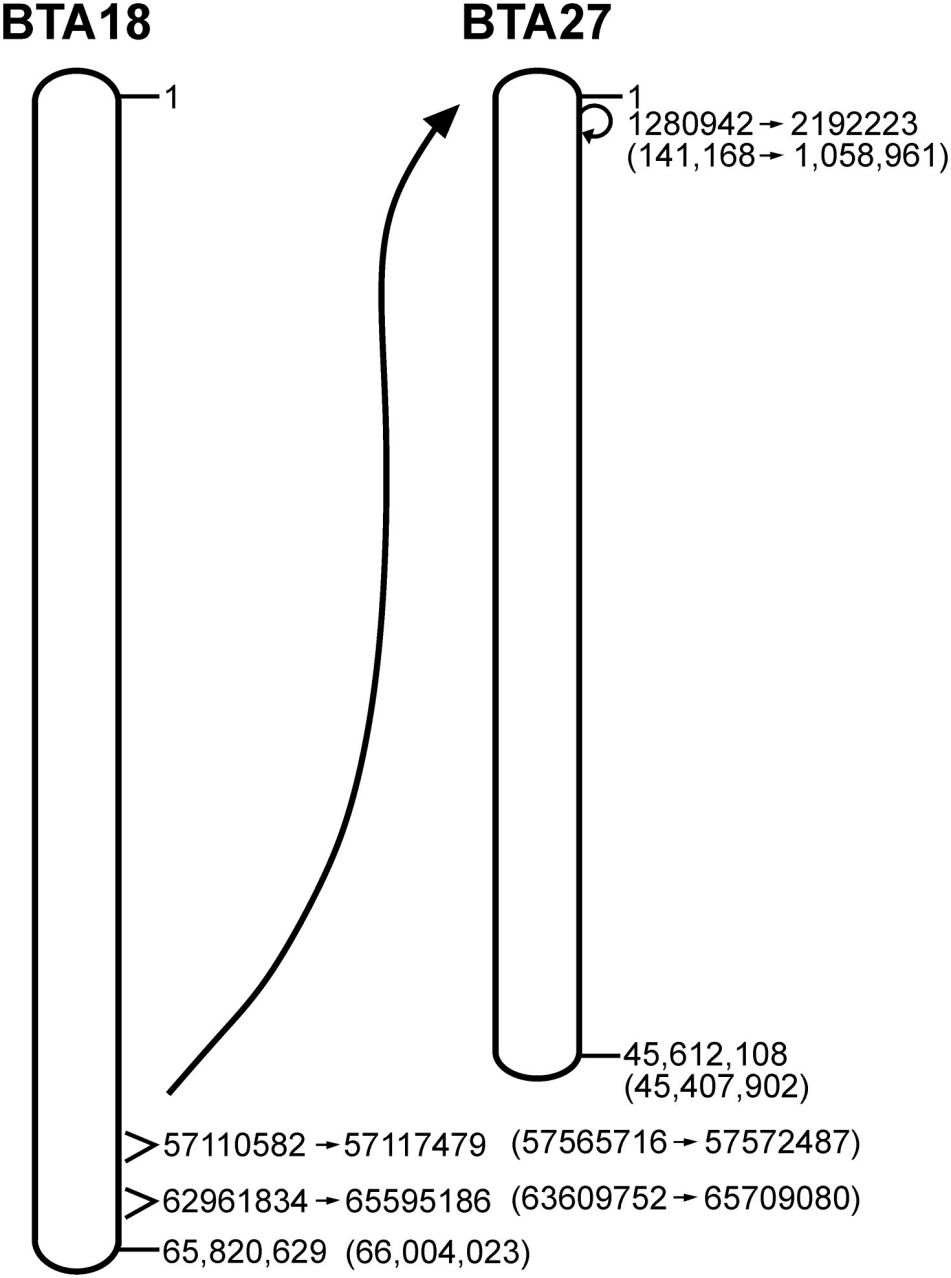
Structural variant analysis for BTA 18 and 27. Presentation of the tandem fusion translocation between the distal end of BTA 18 and proximal end of BTA 27 accompanied by loss of genome sequences. Positions are given according to ARS-UCD1.2 and in brackets according to UMD3.1 bovine reference assembly.

The identified structural variants for BTA 18 and 27, which occurred exclusively in the affected calf, are given in Table 2. Six of these variants agreed with the cytological identified tandem fusion of the distal end of BTA18 and the proximal end of BTA 27 (Fig 8). The region between the breakpoints BTA 18:57,110,582 (UMD3.1: 57,565,716) and BTA 18:65,595,186 (UMD3.1: 65,781,743) consisted of four deletions and one translocation. The deletion from BTA 18:65,593,935 to BTA 18:65,595,186 (UMD3.1: BTA 18:65,780,492 to BTA 18:65,781,743) marked the loss of genome sequences at the distal end of BTA18 occurring due to chromosomal fusion with BTA27. On BTA 27, a fusion between the breakpoints BTA 27:1,280,942 (UMD3.1: 141,168) and BTA 27:2,192,223 (UMD3.1: 1,058,961) were associated with the loss of genome sequences at the proximal end of BTA 27. Within deletions on BTA 18, the genes *CD33* (57,101,773–57,132,189 bp) and *ENSBTAG00000049556* (65,579,823–65,599,864 bp) became partly single-copied and *LILRA2* (62,970,281–62,974,443 bp) single-copied. Function of *CD33* and *LILRA2* were not associated with hypospadias. In summary, none of the detected structural variants included regions with candidate genes associated with hypospadias in previous reports (S1 Table).

**Table 2:** Filtering results for structural BTA18 and BTA27 variants heterozygous (wt/mut) in the affected calf and homozygous (wt/wt) or not detectable (-/-) in the controls according to ARS-UCD1.2. Regions affected by deletions and translocations cytogenetically classified as 59, XY + tan(18;27) (tandem fusion translocation) are in bold.

BTA	Position left breakpoint	Position right breakpoint	Structural variant	Chromosome size (bp)	Genotype		
					Affected calf	Control 1	Control 2
18	49418139	12:62895928	Translocation	66004023	wt/mut	wt/wt	wt/wt
**18**	**57110582**	**18:57117479**	**Deletion**	**66004023**	**wt/mut**	**wt/wt**	**wt/wt**
**18**	**62961834**	**18:62963176**	**Deletion**	**66004023**	**wt/mut**	**wt/wt**	**wt/wt**
**18**	**62964689**	**18:62979859**	**Deletion**	**66004023**	**wt/mut**	**-/-**	**-/-**
**18**	**64520333**	**18:64652617**	**Translocation**	**66004023**	**wt/mut**	**wt/wt**	**wt/wt**
**18**	**64652617**	**18:64520333**	**Translocation**	**66004023**	**wt/mut**	**wt/wt**	**wt/wt**
**18**	**65593935**	**18:65595186**	**Deletion**	**66004023**	**wt/mut**	**wt/wt**	**wt/wt**
**27**	**1280942**	**27:2192223**	**Translocation**	**45407902**	**wt/mut**	**wt/wt**	**wt/wt**
**27**	**2192223**	**27:1280942**	**Translocation**	**45407902**	**wt/mut**	**wt/wt**	**wt/wt**
27	6969231	27:7233126	Duplication	45407902	wt/mut	wt/wt	wt/wt

### Variant detection

Filtering WGS data gave 32,496,112 single base pair variants and 507,7103 indels, restricting on such with an effect on protein function, which were exclusively present in the hypospadias phenotype, revealed 1041 variants (S2 Table). A further filtering of these 1041 variants against candidate genes (S1 Table) retrieved only one variant located within exon 22 of the *diacylglycerol kinase kappa* gene (*DGKK*:g.93219478T>C) on the X chromosome. The affected calf was heterozygous mutated and all controls were wild type. The potential functional effects for this variant due to a single amino acid change (glutamine to arginine) were declared as tolerated (0.05) by SIFT and as benign (0.009) by PolyPhen-2.

## Discussion

Cytogenetic analysis of the examined Holstein calf revealed an abnormal diploid chromosome number (2n = 59, XY) due to a numerical chromosomal aberration (non-mosaic pseudo-monosomy) originated by a tandem fusion translocation between the distal end (telomeres) of BTA 18 and proximal end (centromere) of BTA 27, as also confirmed by FISH-mapping analysis with chromosome specific markers. Structural variant detection of WGS data validated this chromosomal fusion due to heterozygous loss of genome sequences at the distal end of BTA 18 and the proximal end of BTA 27.

The tandem fusion translocations are very rare in cattle. Although most of the studies have been performed in searching Robertsonian translocation rob(1;29), tandem fusion translocations are very easy to detect because the diploid number is reduced to 59 chromosomes as in the present case. An overview in cattle from various European breeds has revealed the presence of different chromosomal abnormalities, but not any animal was carrier of a tandem fusion translocation among the 45,837 cattle examined [45]. The only two previous cases of tandem fusions were those reported by Popescu and Pinheiro [46, 47].

We suggest that, histologically displayed pure monosomy metaphases, in fact were hidden unbalanced translocation due to specific investigations.

In previously reported cases in cattle, sheep and dogs with hypospadias, no chromosomal aberrations of the autosomes and sex chromosomes X and Y in metaphase were registered [22, 24, 25, 48]. On the other hand, in humans, unbalanced translocations were identified causing congenital abnormalities in patients, as fusion (unbalanced translocation) between chromosome 9 and Y [49] or diseases like myelodysplastic syndrome due to chromosome 1 and 7 fusion [50]. Further, monosomy 21 was identified as pseudo-monosomy due to unbalanced translocation between chromosome 18 and 21 [51]. Hypospadias were reported as a phenotype aspect of a patient showing a fusion of chromosome 7 and 22 [52].

In the present case, the X and Y chromosomes were detected in normal shape and the presence of SRY-gene in the Y chromosome was additionally confirmed. Therefore, we are convinced to have excluded structural aberrations of the X and Y chromosome causing the present phenotype of the calf. One variant, a base change, on the X-chromosome was detected in the candidate gene *DGKK*, which is reported as a major risk gene for mild to moderate hypospadias phenotypes in humans [53, 54]. Along with reports on humans, we suggest that this variant was not the causing mutation of the present severe phenotype [54]. Nevertheless, we do not exclude a potential effect due to a mild protein defect because the potential effect of this variant on protein function was classified as tolerated and benign.

The development of male external genitalia is especially dependent on hormonal mediation like androgen and their androgen receptors through enzymatic activities and hormonal transduction signals. However, it also requires a correct genetic program, cellular differentiation, complex tissue, epithelial-mesenchymal interactions [23, 55, 56]. The present chromosomal aberration seems to be a novel defect, which had its origin in the meiotic cell division (meiosis I or II), because the dam and the sire of the calf showed no indication of an abnormal phenotype.

Hypospadias in cattle is a sporadic congenital abnormality, its incidence varying between 0.3% [57] and 0.46% [31], and it seems to be a heterogeneously inherited condition with recessive or partial dominant mode of inheritance. Nevertheless, not all cases of hypospadias in cattle fit into these heredity models [58].

The present case of hypospadias was classified as the perineal type with mainly complete opening of the urethra and incomplete penile and preputial aplasia. Normally, the fusion of the urethral folds starts as a progressive formation proximally in the perineal region and stop distally at the glans penis. Therefore, the closing process of the urethra failed in the beginning, with the final effect of an almost completely open urethral groove. The urination was made by the opening distal the anus.

Hypospadias also coexist with other malformations especially in the genital and anorectal region, like cryptorchidism and atresia ani, which likely indicate an interrelation of these abnormalities [31]. Cryptorchidism describes a unilateral or bilateral insufficient descensus testis. Atresia ani is a congenital occlusion of the lumen of the digestive tract and results from the failure of the anal membranes to degenerate [27]. This probably resulted while the male genital development. The cloacal membrane is enclosed by the urogenital fold to form a septum dividing the urogenital sinus and the hindgut [21, 59]. Consequently, while the differentiation of the genital region, there could be more than one failure together. Both, cryptorchidism and atresia ani are common congenital abnormalities in neonates. In this case, the testis of the calf descended while growing up and no atresia ani was identified. Instead of this, a VSD underneath the aortic valve was identified. A few cases of hypospadias in cattle, which also had cardiovascular abnormalities are reported in eight bulls [31]. VSD is the most common congenital cardiovascular abnormality in calves and occur in 2.7% of the calves, which are affected with further birth defects [60]. VSD was also found in a calf affected by trisomy of BTA 28 and showing inferior brachygnathia, reduced growth and one extra aorta septal cup [61].

The reduced growth rate of the animal examined may correlate with the VSD. After diagnosing hypospadias, the animal and especially the reproductive tract has to be examined in detail to determine other congenital abnormalities.

Chromosome abnormalities are higher in infertile men and domestic animals [62] (between 2 and 10%) and this value increases up to 15% in azoospermic males [63]. Robertsonian translocation are the most frequent structural chromosomal abnormalities both in humans and in cattle, giving the formation of a dicentric abnormal chromosome and the loss of genetic material [63,64]. They can affect fertility with varying degrees of sperm alteration due to possibly impaired gametogenesis. In the tandem fusion translocation, we had a similar situation with the formation of a dicentric abnormal chromosome with two functional but separate centromeres and the loss of genetic material from BTA 18 and 27. In our case, we can assume the lack of spermatogenesis is probably due to the formation of unpaired chromosomes during meiosis for the presence of abnormal trivalent configurations.

Depending on the anatomical location of the urethral opening, a surgical correction is possible [13, 23]. But in general it is not recommended, because of accompanying other congenital abnormalities, especially atresia ani [28]. In this case, no surgery was necessary, because the ability to urinate without indications of infectious complications was given [34].

Cumbersome examination for chromosomal aberrations is a difficult complex process. Cytological preparation combined with FISH is indispensable for rough visual examination, but new techniques, which could give precise information about structural variants, such as Beadchip [62] and NGS, continue to emerge [63, 64]. In this study, we could detect and define a non-mosaic monosomy 59 due to a tandem fusion of two autosomes using the combination of cytogenetic examination and whole genome sequencing data analysis. Furthermore, several cytogenetic reports performed in humans with birth defects including hypospadias, revealed microdeletions or deletions, especially at the telomeric regions [65–69].

In conclusion, we presented the first report of a chromosomal aberration associated with a phenotype of perineal hypospadias, VSD and growth retardation in Holstein cattle. The relevant fusion of chromosome 18 and 27, which cause the non-mosaic pseudo-monosomy 59, was detected using the technical-laboratory combination of cytological and cytogenetic examinations, as well as whole genome sequencing.

## Material and methods

### Statement of Ethics

All animal work has been conducted according to the national and international guidelines for animal welfare. Sampling was approved by the Institutional Animal Care and Use Committee of Lower Saxony, the State Veterinary Office Niedersächsisches Landesamt für Verbraucherschutz und Lebensmittelsicherheit, Oldenburg, Germany (registration number 33.9-42502-05-04A247). Informed written consent from the cattle owner was obtained that the animal and its samples will be used for the present study and publication.

### Animal

The male black and white Holstein calf examined was born on May 7, 2016 on a German dairy farm. The calf was transferred in September 2016 to the Clinic for Cattle of the University of Veterinary Medicine Hannover. According to the owner, this was the first case with genital abnormalities in this dairy herd. The calf was euthanized on October 4, 2018 because of severe lameness in sequel of an accident. Euthanasia was performed by intravenous injection of Ursotamin^®^ (ketaminehydrochloride 100 mg/ml; Serumwerk Bernburg, Bernburg, Germany, 2 mg/kg, intravenous) combined with Xylavet^®^ (xylazine hydrochloride, 20 mg/ml, CP-Pharma, Burgdorf, Germany, 0.1 mg/kg, intravenous) as premedication followed by Release^®^ (pentobarbital-natrium 300 mg/ml, WDT Garbsen, Germany, 450 mg/10 kg body weight, intravenous).

### Clinical examination

The clinical examination of the affected calf included a general health check with auscultation of the cardiovascular and digestive system and screening of laboratory parameters. In this course, a special examination of the genital region and the urethra was performed. In addition, an ultrasound scan of the heart was carried out using the ultrasound scanner MyLab^™^One VET (Esaote Europe, Maastricht, The Netherlands) with a phased array probe (SP3630). The further development of the calf was recorded under controlled clinical conditions over two years.

### Necropsy

At the age of 2.5 years, the animal underwent necropsy after euthanization. All organs were examined for abnormalities, especially the urogenital tract. Tissue samples of the urethra, the rudimentary glans penis and the testicles were fixed and stained with hematoxylin-eosin for histological examination.

### Cell cultures

Peripheral blood samples were cultured in RPMI medium enriched with fetal calf serum (10%), antibiotic-antimycotic mixture (1%), L-glutamine (1%) and concanavalin A (20 μg/ml). Two types of cell cultures were conducted: either without adding any base analog (conventional) or with BrdU to obtain an R-banding (by late incorporation of BrdU) pattern [70, 71], whereas the colcemid treatment (0.1 μg/ml) was performed for the last hour.

### BAC probes

Two BACs belonging to the INRA bovine BAC library [72] were used to confirm the involved chromosomes in the aberration: BAC 651C08, containing the ISCNDB reference marker of BTA18 (GPI) [73], located on BTA 18q24 (position 44560941–44663929 according UMD3.1 genome assembly) and BAC 696G12 located on BTA 27q15-16 (position 24011371–24133841 according UMD3.1 genome assembly). DNA isolation was performed using CHORI- (Children’s Hospital Oakland Research Institute) recommended protocol (https://bacpacresources.org), after overnight growth at 37°C in LB medium supplemented with chloramphenicol. For the FISH experiment, 150 ng of each DNA probe were labeled with biotin and digoxigenin as reported in [74].

### Fluorescence in situ hybridization (FISH)

R-banded slides were treated for FISH analysis with BAC clones overnight in the presence of bovine COT-l DNA and sonicated salmon sperm allocated in a moist chamber following the hybridization protocols of [75]. Finally, chromosomes were counterstained in Vectashield H-1000 (Vector Lab) antifade solution. The images were captured by using a Leica CTR 5500 fluorescence microscope equipped with 100x oil immersion lens, DAPI, FITC, Texas Red specific filters and Photometrics Sensys camera. Thirty metaphase spread plates with double signals were analyzed using Cytovision specific software, and chromosome identification was performed according to ISCNDB 2000 [76].

### Sequential telomere and C-banding techniques

Conventional chromosomes were treated with sequential telomere and C-banding techniques. The telomere PNA probe, mapping on all telomeres, was hybridized on metaphase cells using the telomere PNA FISH kit/FITC (Dako Cytomation). At least 50 cells were analyzed using Cytovision software. After the analysis, the coverslip was removed, and the slide was washed in PBST (PBS 1X and Tween 20) solution, rinsed, and dried before C-banding (CBA). CBA followed the protocols reported in [77], and the same cells as above were analyzed.

### Y chromosome detection

In addition, to confirm the presence of the male specific Y chromosome, a segment of the *SRY* gene, the testis determining factor, was investigated using polymerase chain reaction (PCR). DNA samples from one male and one female Holstein cattle were added as positive and negative controls. DNA was amplified using the following primers according to Meinecke et al. (2003) [78]: forward primer 5´- AGCGCAAATGATCAGTGTG-3´ and reverse primer 5´- CCGTGTAGCCAATGTTACC-3´, which are specific for a 519 bp amplicon of coding sequence of the bovine *SRY* gene (ENSBTAG00000047779). Furthermore, we used the primer pairs forward primer 5´- AGCTTCCCAAGTGAGGAGTC-3´ and reverse primer 5´- TCCCTCTCCTCTCACCTCAA-3´ to produce a control PCR-product of 563 bp from *GON4L* gene [79] on chromosome 3, to verify amplification of the isolated DNA. Both protocols for PCR were run using 58°C as annealing temperature. PCR-products were separated using gel electrophoresis on a 1% agarose gel (Carl Roth, Karlsruhe, Germany), which was stained with ethidium bromide (Carl Roth) and photographed and processed under UV light with Gel iX20 Imager Windows Version (INTAS Science Imaging Instruments, Göttingen, Germany).

### Whole genome sequencing

For whole-genome sequencing, genomic DNA was isolated from an EDTA-blood sample from the *vena jugularis* of the affected calf using a chloroform extraction protocol [80]. Whole genome sequencing was performed on an Illumina NextSeq500 for 2x150 bp in paired-end mode. Mean coverage was at 8.79X with an average mapping rate of 97.7%. For data analysis, quality control was performed. Data files from the calf were mapped to the bovine reference genome ARS-UCD1.2 (Ensembl) using BWA, version 0.7.13 [81]. SAMtools 1.3.1 [82] and Picard tools (http://broadinstitute.github.io/picard/, version 2.9.4) were used for sorting, indexing and marking of duplicates of Bam-files. Variants were called with GATK, version 4.0 [83], using Base Quality Score Recalibration (BQSR), Haplotype Caller and Variant Recalibrator. We took all variants with a read depth >1 and < 1000 and quality score values >20 were chosen from the vcf-file for further analysis.

### Validation by structural variant detection

In order to check for candidate chromosomal aberrations, we used the breakpoint prediction framework LUMPY [84]. We compared data from two private control animals of the breeds Holstein with data from the affected calf. Structural variants were filtered out which were heterozygous mutant in the affected calf and homozygous wild type in both controls. These structural variants in the candidate regions were investigated for their potential functional effects by comparison of their genomic positions with those regions harboring candidate genes for hypospadias in humans and animals according to National Center for Biotechnology Information (NCBI, www.ncbi.nlm.nih.gov). The position of the structural variants in the UMD3.1 genome were compared with the genome database ARS-UCD1.2 using the ncbi genome remapping service (NCBI, www.ncbi.nlm.nih.gov/genome/tools/remap).

### Variant detection

We screened the WGS data for variants which occurred exclusively in the affected calf. Variants with a read depth of 2–999 and quality values >20 were selected. Data of the calf were compared with the data of 93 different variant callings from private controls of the breeds Holstein, Fleckvieh, Braunvieh, Vorderwald, German Angus, Galloway, Limousin, Charolais, Hereford, Tyrolean Grey and Miniature Zebu. We filtered out variants, which were homozygous or heterozygous mutant for the affected calf, and homozygous wild type in all controls using SAS (Statistical Analysis System, Cary, NC, USA), version 9.4. Those variants were chosen for further analysis whose effects were estimated as high or moderate according to prediction toolbox SNPEff, version 4.3 q (2017-08-30, SNPEff database, UMD3.1.86) [85]. Using the Variant Effect Predictor [86] for SIFT predictions [87], we investigated the potential functional effects of the final variants. In the next step, these variants were compared with candidate genes which were previously found to be associated with hypospadias in humans or domestic animals according to National Center for Biotechnology Information (NCBI, www.ncbi.nlm.nih.gov). To verify the previously investigated protein effect of the remaining variant, we applied the PolyPhen-2 (Polymorphism Phenotyping v2) tool.

## Availability of data and materials

Whole genome sequencing data of the affected animal were deposited in NCBI Sequence Read Archive (http://www.ncbi.nlm.nih.gov/sra) under SRA accession number PRJNA544605 (SAMN11843850).

## Supporting information

S1 Table Candidate genes for the hypospadias phenotypes in humans and animals according to NCBI. The bovine orthologues genes are presented and arranged by their chromosomal position.
(DOCX)

S2 Table Results of the filtered whole genome sequencing data. All 1041 variants homozygous or heterozygous mutant exclusively in the affected calf are given. Critical variants for the phenotype hypospadias are in bold.
(DOCX)

S1 Raw Images The file contains raw images of Figs 4–7. (PDF)
