# Supplementary material for: Clinical, cytogenetic and molecular genetic characterization of a tandem fusion translocation in a male Holstein cattle with congenital hypospadias and a ventricular septal defect
Source: PLoS One. 2020 Jan 10;15(1):e0227117. doi: 10.1371/journal.pone.0227117 (PMC6953810; doi:10.1371/journal.pone.0227117)
Supplement: S1 Table — The bovine orthologues genes are presented and arranged by their chromosomal position. (DOCX) [file pone.0227117.s001.docx]

**Table S1.** Candidate genes for the hypospadias phenotypes in human and animals according to NCBI. The bovine orthologues genes are presented and arranged by their chromosomal position.

| **Gene** | **Gene full name** | **Species** | **Gene ID** | **Bovine gene** | **BTA** | **Position** |
| --- | --- | --- | --- | --- | --- | --- |
| *TP63* | *tumor protein p63* | *Homo sapiens* | ENSBTAG00000015460 | *TP63* | 1 | 78012414-78283420 |
| *HOXD13* | *homeobox D13* | *Homo sapiens* | ENSBTAG00000004313 | *HOXD13* | 2 | 20854099-20856159 |
| *OBSL1* | *obscurin like 1* | *Homo sapiens* | ENSBTAG00000040338 | *OBSL1* | 2 | 108203750-108225840 |
| *ZEB2* | *zinc finger E-box binding homeobox 2* | *Homo sapiens* | ENSBTAG00000012615 | *ZEB2* | 2 | 52275074-52406207 |
| *HSD3B2* | *hydroxy-delta-5-steroid dehydrogenase, 3 beta- and steroid delta-isomerase 2* | *Homo sapiens* | ENSBTAG00000006769 | *HSD3B1* | 3 | 23813912-23823807 |
| *HOXA13* | *homeobox A13* | *Homo sapiens* | ENSBTAG00000014735 | *HOXA13* | 4 | 69297275-69298937 |
| *HOXA3* | *homeobox A3* | *Homo sapiens* | ENSBTAG00000008139 | *HOXA3* | 4 | 69357161-69389905 |
| *HOXA4* | *homeobox A4* | *Homo sapiens* | ENSBTAG00000001063 | *HOXA4* | 4 | 69366547-69368811 |
| *IGFBP3* | *insulin like growth factor binding protein 3* | *Homo sapiens* | ENSBTAG00000003994 | *IGFBP3* | 4 | 76705105-76712709 |
| *PIP* | *prolactin induced protein* | *Homo sapiens* | ENSBTAG00000006655 | *PIP* | 4 | 07308521-107309079 |
| *TAX1BP1* | *Tax1 binding protein 1* | *Homo sapiens* | ENSBTAG00000019020 | *TAX1BP1* | 4 | 68775902-68859811 |
| *CCDC59* | *coiled-coil domain containing 59* | *Homo sapiens* | ENSBTAG00000036282 | *CCDC59* | 5 | 11972439-11978864 |
| *FKBP4* | *FK506 binding protein 4* | *Homo sapiens* | ENSBTAG00000007605 | *FKBP4* | 5 | 107445596-107453517 |
| *Mir200c* | *microRNA 200c* | *Rattus norvegicus* | ENSBTAG00000029959 | *bta-mir-200c* | 5 | 103859436-103859500 |
| *Anxa3* | *annexin A3* | *Rattus norvegicus* | ENSBTAG00000010153 | *Anxa3* | 6 | 95065256-95136945 |
| *EBF1* | *EBF transcription factor 1* | *Homo sapiens* | ENSBTAG00000037508 | *EBF1* | 7 | 72396487-72804182 |
| *MAPK9* | *mitogen-activated protein kinase 9* | *Homo sapiens* | ENSBTAG00000004709 | *MAPK9* | 7 | 826695-892386 |
| *HSD17B3* | *hydroxysteroid 17-beta dehydrogenase 3* | *Homo sapiens* | ENSBTAG00000016867 | *HSD17B3* | 8 | 84382336-84436927 |
| *CTGF* | *connective tissue growth factor* | *Homo sapiens* | ENSBTAG00000006367 | *CTGF* | 9 | 70873221-70876455 |
| *ESR1* | *estrogen receptor 1* | *Homo sapiens* | ENSBTAG00000007159 | *ESR1* | 9 | 89969586-90255801 |
| *WTAP* | *WT1 associated protein* | *Homo sapiens* | ENSBTAG00000007974 | *WTAP* | 9 | 97434236-97460620 |
| *ESR2* | *estrogen receptor 2* | *Homo sapiens* | ENSBTAG00000004498 | *ESR2* | 10 | 76707922-76757370 |
| *GREM1* | *gremlin 1, DAN family BMP antagonist* | *Homo sapiens* | ENSBTAG00000046604 | *GREM1* | 10 | 30039413-30039967 |
| *Six1* | *sine oculis-related homeobox 1* | *Mus musculus* | ENSBTAG00000012139 | *SIX1* | 10 | 73068706-73072802 |
| *CYP1B1* | *cytochrome P450 family 1 subfamily B member 1* | *Homo sapiens* | ENSBTAG00000010531 | *CYP1B1* | 11 | 20493493-20499001 |
| *HAAO* | *3-hydroxyanthranilate 3,4-dioxygenase* | *Homo sapiens* | ENSBTAG00000004674 | *HAAO* | 11 | 25187252-25201563 |
| *Notch1* | *notch 1* | *Rattus norvegicus* | ENSBTAG00000022799 | *NOTCH1* | 11 | 103986874-104030692 |
| *NR5A1* | *nuclear receptor subfamily 5 group A member 1* | *Homo sapiens* | ENSBTAG00000009017 | *NR5A1* | 11 | 95514365-95538847 |
| *PKDCC* | *protein kinase domain containing, cytoplasmic* | *Homo sapiens* | ENSBTAG00000019382 | *PKDCC* | 11 | 24529639-24539289 |
| *SRD5A2* | *steroid 5 alpha-reductase 2* | *Homo sapiens* | ENSBTAG00000003108 | *SRD5A2* | 11 | 14347369-14393801 |
| *DGKH* | *diacylglycerol kinase eta* | *Homo sapiens* | ENSBTAG00000013879 | *DGKH* | 12 | 12293695-12405237 |
| *BMP7* | *bone morphogenetic protein 7* | *Homo sapiens* | ENSBTAG00000015362 | *BMP7* | 13 | 59425468-59510393 |
| *MKKS* | *McKusick-Kaufman syndrome* | *Homo sapiens* | ENSBTAG00000034987 | *MKKS* | 13 | 3574758-3582527 |
| *ZEB1* | *zinc finger E-box binding homeobox 1* | *Homo sapiens* | ENSBTAG00000020053 | *ZEB1* | 13 | 34063175-34261299 |
| *EYA1* | *EYA transcriptional coactivator and phosphatase 1* | *Homo sapiens*  *Mus musculus* | ENSBTAG00000011298 | *EYA1* | 14 | 36898122-37268909 |
| *TMEM70* | *transmembrane protein 70* | *Homo sapiens* | ENSBTAG00000012920 | *TMEM70* | 14 | 39354351-39362288 |
| *ST5* | *suppression of tumorigenicity 5* | *Homo sapiens* | ENSBTAG00000005356 | *ST5* | 15 | 44335342-44460360 |
| *WT1* | *Wilms tumor 1* | *Homo sapiens* | ENSBTAG00000047268 | *WT1* | 15 | 63913734-63965294 |
| *ATF3* | *activating transcription factor 3* | *Homo sapiens* | ENSBTAG00000008545 | *ATF3* | 16 | 72820026-72832974 |
| *MTHFR* | *methylenetetrahydrofolate reductase* | *Homo sapiens* | ENSBTAG00000020698 | *MTHFR* | 16 | 42750520-42765210 |
| *FGFR2* | *fibroblast growth factor receptor 2* | *Homo sapiens*  *Mus musculus* | ENSBTAG00000014064 | *FGFR2* | 17 | 35199615-35259135 |
| *PDGFC* | *platelet derived growth factor C* | *Homo sapiens* | ENSBTAG00000043959 | *PDGFC* | 17 | 43348702-43457620 |
| *CCDC8* | *coiled-coil domain containing 8* | *Homo sapiens* | ENSBTAG00000018181 | *CCDC8* | 18 | 54124479-54127099 |
| *IRX5* | *iroquois homeobox 5* | *Homo sapiens* | ENSBTAG00000004838 | *IRX5* | 18 | 23309540-23312757 |
| *IRX6* | *iroquois homeobox 6* | *Homo sapiens* | ENSBTAG00000016407 | *IRX6* | 18 | 23681599-23687572 |
| *ZFHX3* | *zinc finger homeobox 3* | *Homo sapiens* | ENSBTAG00000014636 | *ZFHX3* | 18 | 38325141-38491680 |
| *EXOC3* | *exocyst complex component 3* | *Homo sapiens* | ENSBTAG00000008606 | *EXOC3* | 20 | 71765789-71790498 |
| *SRD5A1* | *steroid 5 alpha-reductase 1* | *Homo sapiens* | ENSBTAG00000015478 | *SRD5A1* | 20 | 66681686-66718713 |
| *CYP11A1* | *cytochrome P450 family 11 subfamily A member 1* | *Homo sapiens* | ENSBTAG00000006934 | *CYP11A1* | 21 | 34724307-34734990 |
| *CYP1A1* | *cytochrome P450 family 1 subfamily A member 1* | *Homo sapiens* | ENSBTAG00000001021 | *CYP1A1* | 21 | 34342808-34345682 |
| *SIN3A* | *SIN3 transcription regulator family member A* | *Homo sapiens* | ENSBTAG00000009985 | *SIN3A* | 21 | 33796382-33840716 |
| *Ctnnb1* | *catenin (cadherin associated protein), beta 1* | *Mus musculus* | ENSBTAG00000016420 | *CTNNB1* | 22 | 13842703-13889468 |
| *EEFSEC* | *eukaryotic elongation factor, selenocysteine-tRNA specific* | *Homo sapiens* | ENSBTAG00000030962 | *EEFSEC* | 22 | 60063340-60172924 |
| *TGFBR2* | *transforming growth factor beta receptor 2* | *Homo sapiens* | ENSBTAG00000019832 | *TGFBR2* | 22 | 5141232-5233083 |
| *CUL7* | *cullin 7* | *Homo sapiens* | ENSBTAG00000012749 | *CUL7* | 23 | 16640766-16654717 |
| *DAAM2* | *dishevelled associated activator of morphogenesis 2* | *Homo sapiens* | ENSBTAG00000021381 | *DAAM2* | 23 | 13775149-13829110 |
| *SRCAP* | *Snf2 related CREBBP activator protein* | *Homo sapiens* | ENSBTAG00000013917 | *N/A* | 25 | 27089883-27117931 |
| *CYP17A1* | *cytochrome P450 family 17 subfamily A member 1* | *Homo sapiens* | ENSBTAG00000014335 | *CYP17A1* | 26 | 23694362-23700404 |
| *DKK1* | *dickkopf WNT signaling pathway inhibitor 1* | *Homo sapiens* | ENSBTAG00000014218 | *DKK1* | 26 | 6852970-6855647 |
| *FGF8* | *fibroblast growth factor 8* | *Homo sapiens*  *Rattus norvegicus* | ENSBTAG00000001530 | *FGF8* | 26 | 22375029-22380835 |
| *ADK* | *adenosine kinase* | *Homo sapiens* | ENSBTAG00000011072 | *ADK* | 28 | 30215525-30732444 |
| *KCNMA1* | *potassium calcium-activated channel subfamily M alpha 1* | *Homo sapiens* | ENSBTAG00000013300 | *KCNMA1* | 28 | 32807351-33587986 |
| *MAPK8* | *mitogen-activated protein kinase 8* | *Homo sapiens* | ENSBTAG00000007876 | *MAPK8* | 28 | 43160797-43196195 |
| *CDKN1C* | *cyclin dependent kinase inhibitor 1C* | *Homo sapiens* | ENSBTAG00000031184 | *CDKN1C* | 29 | 49368787-49370785 |
| *CCDC26* | *CCDC26 long non-coding RNA* | *Homo sapiens* | N/A | *N/A* | N/A | N/A |
| *DNAH6* | *dynein axonemal heavy chain 6* | *Homo sapiens* | N/A | *N/A* | N/A | N/A |
| *FGF10* | *fibroblast growth factor 10* | *Homo sapiens*  *Mus musculus* | N/A | *N/A* | N/A | N/A |
| *HOXA-AS2* | *HOXA cluster antisense RNA 2* | *Homo sapiens* | N/A | *N/A* | N/A | N/A |
| *HYSP3* | *Hypospadias 3, autosomal* | *Homo sapiens* | N/A | *N/A* | N/A | N/A |
| *HYSP4* | *Hypospadias 4, X-linked, susceptibilty to* | *Homo sapiens* | N/A | *N/A* | N/A | N/A |
| *OGS2* | *Opitz G syndrome, type II* | *Homo sapiens* | N/A | *N/A* | N/A | N/A |
| *PHACTR2-AS1* | *PHACTR2 antisense RNA 1* | *Homo sapiens* | N/A | *N/A* | N/A | N/A |
| *AR* | *androgen receptor* | *Homo sapiens* | ENSBTAG00000022255 | *AR* | X | 88410764-88621166 |
| *ATRX* | *ATRX, chromatin remodeler* | *Homo sapiens* | ENSBTAG00000038434 | *ATRX* | X | 79634445-79917815 |
| *BCOR* | *BCL6 corepressor* | *Homo sapiens* | ENSBTAG00000047339 | *BCOR* | X | 108883915-108907652 |
| *DGKK* | *diacylglycerol kinase kappa* | *Homo sapiens* | ENSBTAG00000022962 | *DGKK* | X | 93195774-93365863 |
| *FLNA* | *filamin A* | *Homo sapiens* | ENSBTAG00000011190 | *FLNA* | X | 40310757-40332714 |
| *MAMLD1* | *mastermind like domain containing 1* | *Homo sapiens* | ENSBTAG00000021713 | *N/A* | X | 33285824-33402706 |
| *MID1* | *midline 1* | *Homo sapiens* | ENSBTAG00000010152 | *MID1* | X | 139791005-139928580 |
| *NAA10* | *N(alpha)-acetyltransferase 10, NatA catalytic subunit* | *Homo sapiens* | ENSBTAG00000047702 | *NAA10* | X | 40058809-40063513 |
