# Supplementary material for: Clinical, cytogenetic and molecular genetic characterization of a tandem fusion translocation in a male Holstein cattle with congenital hypospadias and a ventricular septal defect
Source: PLoS One. 2020 Jan 10;15(1):e0227117. doi: 10.1371/journal.pone.0227117 (PMC6953810; doi:10.1371/journal.pone.0227117)
Supplement: S2 Table — All 1041 variants homozygous or heterozygous mutant exclusively in the affected calf are given. Critical variants for the phenotype hypospadias are in bold. (DOCX) [file pone.0227117.s002.docx]

**Table S2.** Results of the filtered whole genome sequencing data. All 1041 variants homozygous or heterozygous mutant exclusively in the affected calf are given. Critical variants for the phenotype hypospadias are in bold.

| **Gene** | **BTA** | **Position** | **Base change** | **Genotype** | **cDNA** | **Protein** | **Transcript** | **SIFT** |
| --- | --- | --- | --- | --- | --- | --- | --- | --- |
| *ENSBTAG00000047093* | 1 | 47062710 | C>G | 0/1 | c.805G>C | p.Glu269Gln | ENSBTAT00000065922 | Deleterious (0) |
| *CIP2A* | 1 | 53741127 | T>C | 0/1 | c.1231A>G | p.Ile411Val | ENSBTAT00000022784 | Tolerated (0.64) |
| *VGLL3* | 1 | 34695114 | C>A | 0/1 | c.207G>T | p.Glu69Asp | ENSBTAT00000037060.4 | Tolerated (0.51) |
| *VGLL3* | 1 | 34695120 | C>A | 0/1 | c.201G>T | p.Glu67Asp | ENSBTAT00000037060.4 | Tolerated (0.58) |
| *GABRR3* | 1 | 41891869 | TC>T | 0/1 | c.559delG | p.Asp187fs | ENSBTAT00000001857.4 | - |
| *ZNF596* | 1 | 45031788 | G>T | 0/1 | c.1050G>T | p.Gln350His | ENSBTAT00000002795.5 | Deleterious (0.03) |
| *NXPE3* | 1 | 46518394 | G>A | 0/1 | c.13XG>A | p.Val444Met | ENSBTAT00000017908.5 | Deleterious (0) |
| *MORC1* | 1 | 54258132 | A>G | 0/1 | c.787T>C | p.Tyr263His | ENSBTAT00000052196.2 | Tolerated (1) |
| *MORC1* | 1 | 54258140 | C>T | 0/1 | c.779G>A | p.Arg260Lys | ENSBTAT00000052196.2 | Tolerated (0.48) |
| *MORC1* | 1 | 54258178 | A>T | 0/1 | c.741T>A | p.Asp247Glu | ENSBTAT00000052196.2 | Tolerated (0.18) |
| *CD80* | 1 | 64905285 | C>T | 0/1 | c.633G>A | p.Met211Ile | ENSBTAT00000024042.4 | Tolerated (0.37) |
| *SLC12A8* | 1 | 70160019 | T>C | 0/1 | c.10XA>G | p.Ile344Val | ENSBTAT00000018208.5 | Tolerated (0.12) |
| *MUC4* | 1 | 71096963 | C>T | 0/1 | c.2974G>A | p.Gly992Ser | ENSBTAT00000019591.5 | Tolerated (1) |
| *BCL6* | 1 | 80198819 | CT>C | 1/1 | c.1972delT | p.Tyr658fs | ENSBTAT00000001978.4 | - |
| *TBCCD1* | 1 | 81263792 | C>A | 0/1 | c.442C>A | p.His148Asn | ENSBTAT00000004087.5 | Tolerated (0.67) |
| *TBCCD1* | 1 | 81263798 | G>C | 0/1 | c.448G>C | p.Ala150Pro | ENSBTAT00000004087.5 | Tolerated (0.09) |
| **Gene** | **BTA** | **Position** | **Base change** | **Genotype** | **cDNA** | **Protein** | **Transcript** | **SIFT** |
| *SI* | 1 | 103152838 | T>A | 0/1 | c.2994T>A | p.Asp998Glu | ENSBTAT00000023366.5 | Tolerated (0.91) |
| SI | 1 | 103152839 | T>C | 0/1 | c.2995T>C | p.Ser999Pro | ENSBTAT00000023366.5 | Tolerated (1) |
| SI | 1 | 103152848 | T>A | 0/1 | c.X04T>A | p.Ser1002Thr | ENSBTAT00000023366.5 | Tolerated (1) |
| SI | 1 | 103152878 | G>A | 0/1 | c.X34G>A | p.Asp1012Asn | ENSBTAT00000023366.5 | Tolerated (1) |
| ENSBTAG00000009622 | 1 | 112558111 | C>A | 0/1 | c.499G>T | p.Ala167Ser | ENSBTAT00000012673.5 | Tolerated (0.13) |
| GMPS | 1 | 112675711 | ATTG>A | 0/1 | c.66 68delCAA | p.Asn23del | ENSBTAT00000017X1.4 | - |
| GMPS | 1 | 112675716 | G>A | 0/1 | c.64C>T | p.His22Tyr | ENSBTAT00000017X1.4 | Tolerated (0.1) |
| AGTR1 | 1 | 120403103 | T>C | 0/1 | c.986A>G | p.Asn329Ser | ENSBTAT00000063575.1 | Tolerated (1) |
| PLOD2 | 1 | 123427521 | A>G | 0/1 | c.1159A>G | p.Lys387Glu | ENSBTAT00000031949.4 | Tolerated (0.58) |
| PLOD2 | 1 | 123427532 | A>T | 0/1 | c.1170A>T | p.Glu390Asp | ENSBTAT00000031949.4 | Tolerated (1) |
| PLOD2 | 1 | 123427545 | G>A | 0/1 | c.1183G>A | p.Val395Met | ENSBTAT00000031949.4 | Tolerated (0.13) |
| ARMC8 | 1 | 131945736 | T>G | 0/1 | c.381A>C | p.Arg127Ser | ENSBTAT00000026578.5 | Tolerated (0.32) |
| CEP63 | 1 | 135875161 | C>T | 0/1 | c.1613G>A | p.Arg538Gln | ENSBTAT00000009235.5 | Tolerated (0.66) |
| CEP63 | 1 | 135875197 | T>TTA | 0/1 | c.1576 1577insTA | p.Gln526fs | ENSBTAT00000009235.5 | - |
| CEP63 | 1 | 135875199 | ACC>A | 0/1 | c.1573 1574delGG | p.Gly525fs | ENSBTAT00000009235.5 | - |
| TUBGCP5 | 2 | 1183869 | T>G | 0/1 | c.1143A>C | p.Glu381Asp | ENSBTAT00000019582.5 | Tolerated (0.53) |
| TUBGCP5 | 2 | 1183874 | C>T | 0/1 | c.1138G>A | p.Ala380Thr | ENSBTAT00000019582.5 | Tolerated (1) |
| TUBGCP5 | 2 | 1183883 | C>T | 0/1 | c.1129G>A | p.Glu377Lys | ENSBTAT00000019582.5 | Tolerated (0.58) |
| TUBGCP5 | 2 | 1183891 | T>C | 0/1 | c.1121A>G | p.Asn374Ser | ENSBTAT00000019582.5 | Tolerated (1) |
| **Gene** | **BTA** | **Position** | **Base change** | **Genotype** | **cDNA** | **Protein** | **Transcript** | **SIFT** |
| MFSD6 | 2 | 5755731 | G>A | 0/1 | c.2018C>T | p.Thr673Ile | ENSBTAT00000010151.4 | Tolerated (0.2) |
| MFSD6 | 2 | 5755741 | T>G | 0/1 | c.2008A>C | p.Met670Leu | ENSBTAT00000010151.4 | Tolerated (0.76) |
| FAM171B | 2 | 9567800 | GT>G | 0/1 | c.1649delA | p.Asn550fs | ENSBTAT00000002209.5 | - |
| FAM171B | 2 | 9567804 | G>C | 0/1 | c.1646C>G | p.Ser549* | ENSBTAT00000002209.5 | - |
| FRZB | 2 | 13756986 | C>CCG | 0/1 | c.641 642insCG | p.Ala215fs | ENSBTAT00000014572.3 | - |
| FRZB | 2 | 13756988 | G>GGT | 0/1 | c.643 644insGT | p.Ala215fs | ENSBTAT00000014572.3 | - |
| FRZB | 2 | 13756990 | AGTAG>A | 0/1 | c.646 649delGTAG | p.Val216fs | ENSBTAT00000014572.3 | - |
| FRZB | 2 | 13757044 | A>C | 0/1 | c.699A>C | p.Glu233Asp | ENSBTAT00000014572.3 | Tolerated (1) |
| TTN | 2 | 18219510 | T>G | 0/1 | c.36357T>G | p.Ile12119Met | ENSBTAT00000061449.2 | - |
| TTN | 2 | 18219550 | C>T | 0/1 | c.36397C>T | p.Leu12133Phe | ENSBTAT00000061449.2 | - |
| TTN | 2 | 18268191 | T>A | 0/1 | c.57689T>A | p.Phe192XTyr | ENSBTAT00000061449.2 | - |
| TTN | 2 | 18X4076 | T>A | 0/1 | c.85314T>A | p.Asp28438Glu | ENSBTAT00000061449.2 | - |
| ITGA6 | 2 | 24150528 | G>C | 0/1 | c.2475C>G | p.Asp825Glu | ENSBTAT00000022960.4 | Tolerated (0.36) |
| ITGA6 | 2 | 24150531 | C>A | 0/1 | c.2472G>T | p.Glu824Asp | ENSBTAT00000022960.4 | Tolerated (0.72) |
| DCAF17 | 2 | 25051322 | T>G | 0/1 | c.1020A>C | p.Leu340Phe | ENSBTAT00000010105.5 | Deleterious (0) |
| FASTKD1 | 2 | 26742831 | A>G | 0/1 | c.221A>G | p.Asn74Ser | ENSBTAT00000055855.1 | Tolerated (0.29) |
| FASTKD1 | 2 | 26742832 | T>G | 0/1 | c.222T>G | p.Asn74Lys | ENSBTAT00000055855.1 | Tolerated (0.1) |
| FASTKD1 | 2 | 26753973 | T>C | 0/1 | c.569T>C | p.Ile190Thr | ENSBTAT00000055855.1 | Tolerated (0.09) |
| FASTKD1 | 2 | 26753979 | G>A | 0/1 | c.575G>A | p.Arg192Gln | ENSBTAT00000055855.1 | Tolerated (1) |
| **Gene** | **BTA** | **Position** | **Base change** | **Genotype** | **cDNA** | **Protein** | **Transcript** | **SIFT** |
| FASTKD1 | 2 | 26753990 | G>C | 0/1 | c.586G>C | p.Glu196Gln | ENSBTAT00000055855.1 | Tolerated (0.11) |
| FASTKD1 | 2 | 26753993 | A>C | 0/1 | c.589A>C | p.Lys197Gln | ENSBTAT00000055855.1 | Tolerated (0.74) |
| FASTKD1 | 2 | 26754008 | G>A | 0/1 | c.604G>A | p.Ala202Thr | ENSBTAT00000055855.1 | Tolerated (0.62) |
| FASTKD1 | 2 | 26754012 | A>AAC | 0/1 | c.609 610insCA | p.Leu204fs | ENSBTAT00000055855.1 | - |
| FASTKD1 | 2 | 26754014 | TTA>T | 0/1 | c.611 612delTA | p.Leu204fs | ENSBTAT00000055855.1 | - |
| FASTKD1 | 2 | 26754031 | G>A | 0/1 | c.627G>A | p.Met209Ile | ENSBTAT00000055855.1 | Tolerated (0.87) |
| FASTKD1 | 2 | 26754047 | AAT>A | 0/1 | c.645 646delTA | p.Thr216fs | ENSBTAT00000055855.1 | - |
| FASTKD1 | 2 | 26754051 | C>CGT | 0/1 | c.647 648insGT | p.Ala217fs | ENSBTAT00000055855.1 | - |
| LRP2 | 2 | 26989160 | G>C | 0/1 | c.2931G>C | p.Met977Ile | ENSBTAT00000005988.5 | - |
| LRP2 | 2 | 26994712 | G>A | 0/1 | c.3811G>A | p.Gly1271Arg | ENSBTAT00000005988.5 | - |
| CDX2 | 2 | 36675831 | A>G | 0/1 | c.353A>G | p.Gln118Arg | ENSBTAT00000056635.1 | Tolerated (0.11) |
| CDX2 | 2 | 36675840 | A>G | 0/1 | c.362A>G | p.Asp121Gly | ENSBTAT00000056635.1 | Tolerated (0.16) |
| CDX2 | 2 | 36675847 | G>T | 0/1 | c.369G>T | p.Glu123Asp | ENSBTAT00000056635.1 | Tolerated (1) |
| CDX2 | 2 | 36675889 | T>A | 0/1 | c.411T>A | p.Asp137Glu | ENSBTAT00000056635.1 | Tolerated (1) |
| NEB | 2 | 44651378 | G>A | 0/1 | c.11198G>A | p.Arg3733Lys | ENSBTAT00000061000.2 | Tolerated (1) |
| NEB | 2 | 44651390 | A>G | 0/1 | c.11210A>G | p.Tyr3737Cys | ENSBTAT00000061000.2 | - |
| NEB | 2 | 44712320 | G>A | 0/1 | c.17059G>A | p.Glu5687Lys | ENSBTAT00000061000.2 | Deleterious (0.02) |
| NEB | 2 | 44712323 | A>T | 0/1 | c.17062A>T | p.Thr5688Ser | ENSBTAT00000061000.2 | - |
| STEAP3 | 2 | 71424415 | T>G | 0/1 | c.1408T>G | p.Ser470Ala | ENSBTAT00000009356.4 | Tolerated (1) |
| **Gene** | **BTA** | **Position** | **Base change** | **Genotype** | **cDNA** | **Protein** | **Transcript** | **SIFT** |
| STEAP3 | 2 | 71424422 | G>A | 0/1 | c.1415G>A | p.Arg472Lys | ENSBTAT00000009356.4 | Tolerated (0.96) |
| STEAP3 | 2 | 71424436 | G>A | 0/1 | c.1429G>A | p.Val477Met | ENSBTAT00000009356.4 | Tolerated (0.14) |
| NABP1 | 2 | 80648966 | T>C | 0/1 | c.5XT>C | p.Ile177Thr | ENSBTAT00000024821.4 | Tolerated (0.41) |
| NABP1 | 2 | 80648981 | C>A | 0/1 | c.545C>A | p.Pro182Gln | ENSBTAT00000024821.4 | Deleterious (0.05) |
| BMPR2 | 2 | 91459221 | A>G | 0/1 | c.1987A>G | p.Ile663Val | ENSBTAT00000008419.4 | Tolerated (0.94) |
| BMPR2 | 2 | 91459223 | A>G | 0/1 | c.1989A>G | p.Ile663Met | ENSBTAT00000008419.4 | Tolerated (0.13) |
| INO80D | 2 | 94812743 | C>A | 0/1 | c.1203G>T | p.Leu401Phe | ENSBTAT00000038286.4 | Tolerated (1) |
| MAP2 | 2 | 97952656 | A>C | 0/1 | c.537A>C | p.Glu179Asp | ENSBTAT00000024134.4 | Tolerated low confidence(0.08) |
| MAP2 | 2 | 97952663 | C>T | 0/1 | c.544C>T | p.Pro182Ser | ENSBTAT00000024134.4 | Tolerated low confidence(1) |
| IKZF2 | 2 | 101594593 | T>C | 0/1 | c.106A>G | p.Ser36Gly | ENSBTAT00000034358.3 | Tolerated (0.45) |
| CXCR1 | 2 | 106937561 | A>T | 0/1 | c.1019T>A | p.Leu340* | ENSBTAT00000038197.4 | - |
| SLC23A3 | 2 | 107839433 | C>T | 0/1 | c.949G>A | p.Asp317Asn | ENSBTAT00000016751.5 | Tolerated (0.13) |
| ACSL3 | 2 | 111879554 | C>T | 0/1 | c.1570C>T | p.Pro524Ser | ENSBTAT00000022939.5 | Deleterious (0) |
| ACSL3 | 2 | 111879595 | G>A | 0/1 | c.1611G>A | p.Met537Ile | ENSBTAT00000022939.5 | Deleterious (0.05) |
| COL4A3 | 2 | 116249782 | A>G | 0/1 | c.1508A>G | p.Glu503Gly | ENSBTAT00000028418.5 | - |
| COL4A3 | 2 | 116249785 | C>A | 0/1 | c.1511C>A | p.Pro504Gln | ENSBTAT00000028418.5 | - |
| COL4A3 | 2 | 116288462 | A>C | 0/1 | c.4582A>C | p.Met1528Leu | ENSBTAT00000028418.5 | - |
| COL4A3 | 2 | 116288467 | A>G | 0/1 | c.4587A>G | p.Ile1529Met | ENSBTAT00000028418.5 | - |
| **Gene** | **BTA** | **Position** | **Base change** | **Genotype** | **cDNA** | **Protein** | **Transcript** | **SIFT** |
| PHC2 | 2 | 121184283 | A>T | 0/1 | c.712A>T | p.Thr238Ser | ENSBTAT00000006041.5 | Tolerated (0.79) |
| IL22RA1 | 2 | 129371241 | A>C | 0/1 | c.555A>C | p.Glu185Asp | ENSBTAT00000001455.3 | Deleterious (0.04) |
| IL22RA1 | 2 | 129371243 | T>A | 0/1 | c.557T>A | p.Phe186Tyr | ENSBTAT00000001455.3 | Tolerated (1) |
| IL22RA1 | 2 | 129371251 | G>A | 0/1 | c.565G>A | p.Val189Ile | ENSBTAT00000001455.3 | Tolerated (0.69) |
| IL22RA1 | 2 | 129371261 | C>G | 0/1 | c.575C>G | p.Thr192Ser | ENSBTAT00000001455.3 | Tolerated (0.21) |
| IL22RA1 | 2 | 129371292 | G>C | 0/1 | c.606G>C | p.Met202Ile | ENSBTAT00000001455.3 | Tolerated (0.38) |
| IL22RA1 | 2 | 129371297 | G>C | 0/1 | c.611G>C | p.Cys204Ser | ENSBTAT00000001455.3 | Tolerated (0.4) |
| IL22RA1 | 2 | 129371299 | A>G | 0/1 | c.613A>G | p.Ile205Val | ENSBTAT00000001455.3 | Tolerated (1) |
| IL22RA1 | 2 | 129371310 | G>T | 0/1 | c.624G>T | p.Leu208Phe | ENSBTAT00000001455.3 | Tolerated (0.3) |
| VWA5B1 | 2 | 132914461 | C>T | 0/1 | c.1055G>A | p.Arg352His | ENSBTAT00000017965.5 | Tolerated (1) |
| UBR4 | 2 | 134079724 | G>C | 0/1 | c.3193G>C | p.Ala1065Pro | ENSBTAT00000020277.5 | - |
| UBR4 | 2 | 134079750 | A>C | 0/1 | c.3219A>C | p.Glu1073Asp | ENSBTAT00000020277.5 | - |
| UBR4 | 2 | 134079761 | G>C | 0/1 | c.32XG>C | p.Ser1077Thr | ENSBTAT00000020277.5 | - |
| UBR4 | 2 | 134079792 | C>A | 0/1 | c.3261C>A | p.Asp1087Glu | ENSBTAT00000020277.5 | - |
| ATP13A2 | 2 | 136181328 | G>T | 0/1 | c.29XG>T | p.Arg977Leu | ENSBTAT00000010946.5 | Tolerated (0.44) |
| ATP13A2 | 2 | 136181331 | T>C | 0/1 | c.2933T>C | p.Val978Ala | ENSBTAT00000010946.5 | Tolerated (0.35) |
| ADCY10 | 3 | 904504 | G>C | 0/1 | c.4590G>C | p.Glu15XAsp | ENSBTAT00000001400.4 | Tolerated (1) |
| RXRG | 3 | 3615068 | A>G | 0/1 | c.385A>G | p.Thr129Ala | ENSBTAT00000022350.3 | Tolerated (0.42) |
| NIT1 | 3 | 8394334 | C>A | 0/1 | c.299G>T | p.Arg100Leu | ENSBTAT00000026843.5 | Tolerated (1) |
| **Gene** | **BTA** | **Position** | **Base change** | **Genotype** | **cDNA** | **Protein** | **Transcript** | **SIFT** |
| NIT1 | 3 | 8394343 | T>G | 0/1 | c.290A>C | p.Glu97Ala | ENSBTAT00000026843.5 | Tolerated (0.8) |
| NECTIN4 | 3 | 84X873 | A>G | 0/1 | c.188A>G | p.Glu63Gly | ENSBTAT00000023767.4 | Tolerated (0.43) |
| NECTIN4 | 3 | 84X912 | G>C | 0/1 | c.227G>C | p.Gly76Ala | ENSBTAT00000023767.4 | Tolerated (0.81) |
| RRNAD1 | 3 | 14150725 | T>C | 0/1 | c.217A>G | p.Arg73Gly | ENSBTAT00000001990.2 | Tolerated (0.54) |
| GATAD2B | 3 | 16639627 | T>G | 0/1 | c.650T>G | p.Val217Gly | ENSBTAT00000008766.2 | Tolerated (0.53) |
| GATAD2B | 3 | 166396X | G>C | 0/1 | c.653G>C | p.Gly218Ala | ENSBTAT00000008766.2 | Deleterious (0.01) |
| LINGO4 | 3 | 19021206 | T>G | 0/1 | c.922T>G | p.SerX8Ala | ENSBTAT00000054778.2 | Tolerated (0.07) |
| ZNF687 | 3 | 19557312 | C>T | 0/1 | c.139G>A | p.Val47Ile | ENSBTAT00000033811.3 | Tolerated low confidence(0.35) |
| ZNF687 | 3 | 19557319 | T>A | 0/1 | c.132A>T | p.Glu44Asp | ENSBTAT00000033811.3 | Tolerated (0.22) |
| VPS72 | 3 | 19677714 | G>GCTGGATCCTACTCCCACAGCTTCTGCACTGACTCCCCATGCCGGCACTGGACCCGTTGTCCCTCCACC | 0/1 | c.707+1 707+2insCTGGATCCTACTCCCACAGCTTCTGCACTGACTCCCCATGCCGGCACTGGACCCGTTGTCCCTCCACC |  |  |  |
| FAM63A | 3 | 19812780 | A>G | 0/1 | c.533A>G | p.Lys178Arg | ENSBTAT00000033792.2 | Tolerated (0.13) |
| APH1A | 3 | 20416098 | GT>G | 1/1 | c.21delT | p.Phe7fs | ENSBTAT00000046131.3 | - |
| MTMR11 | 3 | 20722184 | A>G | 0/1 | c.832A>G | p.Met278Val | ENSBTAT00000016579.5 | Tolerated (0.65) |
| MTMR11 | 3 | 20722200 | G>C | 0/1 | c.848G>C | p.Ser283Thr | ENSBTAT00000016579.5 | Tolerated (0.08) |
| RNF115 | 3 | 21705042 | A>G | 0/1 | c.803A>G | p.Gln268Arg | ENSBTAT00000010691.4 | Tolerated (1) |
| RNF115 | 3 | 21705055 | A>C | 0/1 | c.816A>C | p.Arg272Ser | ENSBTAT00000010691.4 | Tolerated (1) |
| **Gene** | **BTA** | **Position** | **Base change** | **Genotype** | **cDNA** | **Protein** | **Transcript** | **SIFT** |
| RNF115 | 3 | 21705060 | G>A | 0/1 | c.821G>A | p.Gly274Glu | ENSBTAT00000010691.4 | Tolerated (1) |
| PDZK1 | 3 | 21780618 | A>C | 0/1 | c.488A>C | p.Lys163Thr | ENSBTAT00000007638.5 | Tolerated (1) |
| PDZK1 | 3 | 21780657 | A>G | 0/1 | c.527A>G | p.Asp176Gly | ENSBTAT00000007638.5 | Tolerated (0.38) |
| CHD1L | 3 | 22489051 | C>A | 0/1 | c.2422G>T | p.Ala808Ser | ENSBTAT00000027762.4 | Tolerated (1) |
| PDE4DIP | 3 | 22942199 | A>C | 0/1 | c.4519T>G | p.Ser1507Ala | ENSBTAT00000061284.2 | Tolerated (0.69) |
| GDAP2 | 3 | 25436048 | A>AGGC | 0/1 | c.815 816insGGC | p.Asp272delinsGluAla | ENSBTAT00000021791.5 | - |
| IGSF3 | 3 | 26764919 | A>G | 0/1 | c.946A>G | p.Met316Val | ENSBTAT00000006374.5 | Tolerated (0.61) |
| AMY2B | 3 | 39934028 | C>T | 0/1 | c.854G>A | p.Ser285Asn | ENSBTAT00000025421.3 | Tolerated (0.81) |
| C1orf146 | 3 | 51327560 | A>G | 0/1 | c.278T>C | p.Phe93Ser | ENSBTAT00000040290.4 | Tolerated (0.47) |
| C1orf146 | 3 | 51327577 | A>C | 0/1 | c.261T>G | p.Asn87Lys | ENSBTAT00000040290.4 | Deleterious (0.03) |
| CLCA2 | 3 | 57779572 | T>G | 0/1 | c.766A>C | p.Ser256Arg | ENSBTAT00000033512.4 | Tolerated (0.09) |
| MCOLN3 | 3 | 59272649 | T>C | 0/1 | c.902T>C | p.IleX1Thr | ENSBTAT00000022589.5 | Tolerated (0.14) |
| MCOLN3 | 3 | 59272657 | G>C | 0/1 | c.910G>C | p.ValX4Leu | ENSBTAT00000022589.5 | Tolerated (0.22) |
| MCOLN3 | 3 | 59272669 | G>A | 0/1 | c.922G>A | p.ValX8Met | ENSBTAT00000022589.5 | Deleterious (0.01) |
| ASB17 | 3 | 69195375 | A>G | 0/1 | c.239A>G | p.His80Arg | ENSBTAT00000007708.5 | Tolerated low confidence(1) |
| ASB17 | 3 | 69195379 | A>C | 0/1 | c.243A>C | p.Leu81Phe | ENSBTAT00000007708.5 | Tolerated low confidence(1) |
| ASB17 | 3 | 69195383 | G>C | 0/1 | c.247G>C | p.Val83Leu | ENSBTAT00000007708.5 | Tolerated low confidence(1) |
| **Gene** | **BTA** | **Position** | **Base change** | **Genotype** | **cDNA** | **Protein** | **Transcript** | **SIFT** |
| TNNI3K | 3 | 70548792 | T>C | 0/1 | c.2252A>G | p.Asn751Ser | ENSBTAT00000001278.5 | Tolerated low confidence(1) |
| LEPR | 3 | 80092100 | G>A | 0/1 | c.2101C>T | p.Leu701Phe | ENSBTAT00000007764.4 | Tolerated (0.97) |
| LEPR | 3 | 80092126 | A>G | 0/1 | c.2075T>C | p.Leu692Ser | ENSBTAT00000007764.4 | Tolerated (0.81) |
| LEPR | 3 | 80092141 | T>C | 0/1 | c.2060A>G | p.His687Arg | ENSBTAT00000007764.4 | Tolerated (1) |
| LEPR | 3 | 80092168 | C>T | 0/1 | c.2033G>A | p.Arg678Lys | ENSBTAT00000007764.4 | Tolerated (1) |
| DOCK7 | 3 | 83592311 | A>G | 0/1 | c.3244A>G | p.Ile1082Val | ENSBTAT00000026487.5 | Tolerated (0.85) |
| ZFYVE9 | 3 | 94690239 | C>A | 0/1 | c.1185G>T | p.Glu395Asp | ENSBTAT00000018986.5 | Tolerated low confidence(0.19) |
| ZFYVE9 | 3 | 94690248 | T>G | 0/1 | c.1176A>C | p.Glu392Asp | ENSBTAT00000018986.5 | Tolerated low confidence(0.44) |
| ZFYVE9 | 3 | 94691012 | G>C | 0/1 | c.412C>G | p.Leu138Val | ENSBTAT00000018986.5 | Tolerated low confidence(0.28) |
| ZFYVE9 | 3 | 94691039 | C>G | 0/1 | c.385G>C | p.Glu129Gln | ENSBTAT00000018986.5 | Tolerated low confidence(0.07) |
| ZFYVE9 | 3 | 94691042 | C>T | 0/1 | c.382G>A | p.Val128Ile | ENSBTAT00000018986.5 | Tolerated low confidence(0.15) |
| NSUN4 | 3 | 100351760 | T>A | 0/1 | c.1105A>T | p.Met369Leu | ENSBTAT00000021120.5 | Tolerated (0.11) |
| NSUN4 | 3 | 100351817 | T>C | 0/1 | c.1048A>G | p.Asn350Asp | ENSBTAT00000021120.5 | Tolerated (1) |
| TSPAN1 | 3 | 100499754 | C>T | 0/1 | c.196G>A | p.Val66Met | ENSBTAT00000017715.2 | Deleterious (0.04) |
| HECTD3 | 3 | 101623710 | G>A | 0/1 | c.406G>A | p.Asp136Asn | ENSBTAT00000025X7.4 | Tolerated (0.06) |
| ENSBTAG00000047871 | 3 | 102526773 | T>C | 0/1 | c.1031A>G | p.Lys344Arg | ENSBTAT00000063796.1 | Tolerated (0.71) |
| **Gene** | **BTA** | **Position** | **Base change** | **Genotype** | **cDNA** | **Protein** | **Transcript** | **SIFT** |
| ENSBTAG00000047871 | 3 | 102526786 | G>A | 0/1 | c.1018C>T | p.His340Tyr | ENSBTAT00000063796.1 | Tolerated (0.7) |
| ENSBTAG00000047871 | 3 | 102526788 | T>C | 0/1 | c.1016A>G | p.Lys339Arg | ENSBTAT00000063796.1 | Tolerated (1) |
| TIE1 | 3 | 1032976X | A>G | 0/1 | c.37T>C | p.Phe13Leu | ENSBTAT00000004917.4 | Tolerated (1) |
| SFPQ | 3 | 111143131 | A>G | 0/1 | c.1294A>G | p.Ser432Gly | ENSBTAT00000021719.5 | Tolerated (0.36) |
| SFPQ | 3 | 111143134 | G>A | 0/1 | c.1297G>A | p.Glu433Lys | ENSBTAT00000021719.5 | Deleterious (0.02) |
| CSMD2 | 3 | 112791259 | A>G | 0/1 | c.5278A>G | p.Thr1760Ala | ENSBTAT00000007609.5 | Tolerated (0.13) |
| CSMD2 | 3 | 112831631 | G>A | 0/1 | c.6700G>A | p.Val2234Ile | ENSBTAT00000007609.5 | Tolerated (0.31) |
| MROH2A | 3 | 114057093 | G>T | 0/1 | c.2548C>A | p.Leu850Met | ENSBTAT00000023177.5 | Tolerated (1) |
| MROH2A | 3 | 114057102 | T>C | 0/1 | c.2539A>G | p.Thr847Ala | ENSBTAT00000023177.5 | Tolerated (0.77) |
| ENSBTAG00000027319 | 4 | 8567044 | C>T | 0/1 | c.545C>T | p.Ser182Leu | ENSBTAT00000039166.4 | Tolerated (0.12) |
| ENSBTAG00000027319 | 4 | 8567058 | A>T | 0/1 | c.559A>T | p.Thr187Ser | ENSBTAT00000039166.4 | Tolerated (0.86) |
| ENSBTAG00000027319 | 4 | 8567093 | A>G | 0/1 | c.594A>G | p.Ile198Met | ENSBTAT00000039166.4 | Deleterious (0) |
| CASD1 | 4 | 11779288 | C>G | 0/1 | c.1996C>G | p.Gln666Glu | ENSBTAT00000012005.5 | Tolerated (1) |
| FAM185A | 4 | 44370428 | T>C | 0/1 | c.1051T>C | p.Phe351Leu | ENSBTAT00000019809.4 | Tolerated (1) |
| FAM185A | 4 | 44370431 | C>A | 0/1 | c.1054C>A | p.Leu352Met | ENSBTAT00000019809.4 | Tolerated (1) |
| FAM185A | 4 | 44370440 | A>G | 0/1 | c.1063A>G | p.Thr355Ala | ENSBTAT00000019809.4 | Tolerated (1) |
| GPR22 | 4 | 48789091 | G>C | 0/1 | c.809G>C | p.Ser270Thr | ENSBTAT00000064577.1 | Tolerated (0.77) |
| EEPD1 | 4 | 61358692 | C>A | 0/1 | c.1468G>T | p.Ala490Ser | ENSBTAT00000033712.3 | Tolerated (1) |
| AVL9 | 4 | 64372037 | A>G | 0/1 | c.706T>C | p.Ser236Pro | ENSBTAT00000004049.4 | Tolerated (0.26) |
| **Gene** | **BTA** | **Position** | **Base change** | **Genotype** | **cDNA** | **Protein** | **Transcript** | **SIFT** |
| AVL9 | 4 | 64372043 | G>A | 0/1 | c.700C>T | p.Pro234Ser | ENSBTAT00000004049.4 | Tolerated (1) |
| AVL9 | 4 | 64372046 | C>T | 0/1 | c.697G>A | p.Val233Met | ENSBTAT00000004049.4 | Tolerated (0.14) |
| AVL9 | 4 | 64372052 | C>G | 0/1 | c.691G>C | p.Val231Leu | ENSBTAT00000004049.4 | Tolerated (0.38) |
| AVL9 | 4 | 64372056 | T>A | 0/1 | c.687A>T | p.Glu229Asp | ENSBTAT00000004049.4 | Tolerated (1) |
| AVL9 | 4 | 64372061 | C>T | 0/1 | c.682G>A | p.Asp228Asn | ENSBTAT00000004049.4 | Tolerated (0.07) |
| AVL9 | 4 | 64372067 | G>A | 0/1 | c.676C>T | p.Pro226Ser | ENSBTAT00000004049.4 | Tolerated (1) |
| NFE2L3 | 4 | 70212091 | C>T | 0/1 | c.1792G>A | p.Val598Ile | ENSBTAT00000005413.3 | Tolerated (0.85) |
| NFE2L3 | 4 | 70212204 | C>T | 0/1 | c.1679G>A | p.Ser560Asn | ENSBTAT00000005413.3 | Tolerated (0.39) |
| NFE2L3 | 4 | 70212232 | T>C | 0/1 | c.1651A>G | p.Ile551Val | ENSBTAT00000005413.3 | Tolerated (0.5) |
| C7orf31 | 4 | 71150111 | C>A | 0/1 | c.147C>A | p.Phe49Leu | ENSBTAT00000025898.4 | Tolerated (0.94) |
| C7orf31 | 4 | 71150115 | C>G | 0/1 | c.151C>G | p.Gln51Glu | ENSBTAT00000025898.4 | Tolerated (1) |
| C7orf31 | 4 | 71150128 | G>A | 0/1 | c.164G>A | p.Arg55Gln | ENSBTAT00000025898.4 | Tolerated (0.45) |
| C7orf31 | 4 | 71150171 | G>A | 0/1 | c.207G>A | p.Met69Ile | ENSBTAT00000025898.4 | Tolerated (1) |
| C7orf31 | 4 | 71150208 | T>TATC | 0/1 | c.244 245insATC | p.Cys82delinsTyrArg | ENSBTAT00000025898.4 | - |
| C7orf31 | 4 | 71150210 | TGAACC>T | 0/1 | c.247 251delGAACC | p.Glu83fs | ENSBTAT00000025898.4 | - |
| C7orf31 | 4 | 71150216 | TCCTAGAG>T | 0/1 | c.253 259delCCTAGAG | p.Pro85fs | ENSBTAT00000025898.4 | - |
| CFAP69 | 4 | 74946775 | A>G | 0/1 | c.601A>G | p.Ile201Val | ENSBTAT00000004561.3 | Tolerated (1) |
| CFAP69 | 4 | 74946788 | A>G | 0/1 | c.614A>G | p.Glu205Gly | ENSBTAT00000004561.3 | Deleterious (0.03) |
| CFAP69 | 4 | 74946789 | G>C | 0/1 | c.615G>C | p.Glu205Asp | ENSBTAT00000004561.3 | Tolerated (0.11) |
| **Gene** | **BTA** | **Position** | **Base change** | **Genotype** | **cDNA** | **Protein** | **Transcript** | **SIFT** |
| LMOD2 | 4 | 88746465 | A>C | 0/1 | c.148A>C | p.Met50Leu | ENSBTAT00000002790.5 | Tolerated (1) |
| LMOD2 | 4 | 88746467 | G>A | 0/1 | c.150G>A | p.Met50Ile | ENSBTAT00000002790.5 | Deleterious (0.03) |
| HYAL4 | 4 | 88965279 | G>A | 0/1 | c.259G>A | p.Val87Ile | ENSBTAT00000007294.4 | Tolerated (0.14) |
| HYAL4 | 4 | 88965327 | C>T | 0/1 | c.X7C>T | p.Pro103Ser | ENSBTAT00000007294.4 | Tolerated (1) |
| TNPO3 | 4 | 937449X | A>T | 0/1 | c.1768T>A | p.Leu590Met | ENSBTAT00000006573.5 | Tolerated (0.12) |
| TCAF2 | 4 | 107814184 | G>C | 0/1 | c.779C>G | p.Ala260Gly | ENSBTAT00000007200.4 | Tolerated (0.11) |
| OR6B1 | 4 | 108022496 | C>A | 0/1 | c.433C>A | p.His145Asn | ENSBTAT00000052577.2 | Deleterious (0.01) |
| SSPO | 4 | 113469158 | G>T | 0/1 | c.8974G>T | p.Ala2992Ser | ENSBTAT00000043975.3 | - |
| ZDHHC17 | 5 | 6247X6 | A>G | 0/1 | c.1192A>G | p.Ile398Val | ENSBTAT00000028999.3 | Tolerated (1) |
| SLC6A15 | 5 | 14573318 | T>C | 0/1 | c.2066A>G | p.Asn689Ser | ENSBTAT00000024524.1 | Tolerated low confidence(0.87) |
| SLC6A15 | 5 | 14573388 | G>C | 0/1 | c.1996C>G | p.Pro666Ala | ENSBTAT00000024524.1 | Tolerated (0.85) |
| CEP290 | 5 | 17944847 | G>T | 0/1 | c.3937C>A | p.Leu1313Met | ENSBTAT00000005450.4 | Tolerated (1) |
| CEP290 | 5 | 17944859 | T>C | 0/1 | c.3925A>G | p.Lys1X9Glu | ENSBTAT00000005450.4 | Tolerated (0.5) |
| CEP290 | 5 | 17944862 | G>T | 0/1 | c.3922C>A | p.Leu1X8Met | ENSBTAT00000005450.4 | - |
| CEP290 | 5 | 17944898 | C>G | 0/1 | c.3886G>C | p.Glu1296Gln | ENSBTAT00000005450.4 | Tolerated (0.71) |
| DUSP6 | 5 | 19276876 | T>C | 0/1 | c.1075A>G | p.Thr359Ala | ENSBTAT00000006022.4 | Tolerated (0.63) |
| SP1 | 5 | 268033X | T>C | 0/1 | c.94A>G | p.Ser32Gly | ENSBTAT00000003929.4 | Tolerated low confidence(1) |
| NCKAP5L | 5 | X260819 | TC>T | 0/1 | c.3217delC | p.Gln1073fs | ENSBTAT00000006377.4 | - |
| **Gene** | **BTA** | **Position** | **Base change** | **Genotype** | **cDNA** | **Protein** | **Transcript** | **SIFT** |
| TUBA1A | 5 | X824778 | C>T | 0/1 | c.640C>T | p.Arg214Cys | ENSBTAT00000001948.5 | Tolerated low confidence(0.09) |
| TUBA1A | 5 | X824799 | C>T | 0/1 | c.661C>T | p.Arg221Cys | ENSBTAT00000001948.5 | Deleterious low confidence(0.03) |
| TUBA1A | 5 | X824821 | A>G | 0/1 | c.683A>G | p.Asn228Ser | ENSBTAT00000001948.5 | Deleterious low confidence(0) |
| OR8S1 | 5 | 31634122 | G>A | 0/1 | c.74C>T | p.Ala25Val | ENSBTAT00000055858.2 | Tolerated (0.34) |
| OR8S1 | 5 | 31634128 | G>A | 0/1 | c.68C>T | p.Thr23Ile | ENSBTAT00000055858.2 | Tolerated (1) |
| ANO6 | 5 | 35059726 | C>CTGT | 0/1 | c.2553 2554insACA | p.Val851 Asp852insThr | ENSBTAT00000003770.4 | - |
| ANO6 | 5 | 35059735 | C>G | 0/1 | c.2545G>C | p.Ala849Pro | ENSBTAT00000003770.4 | Tolerated (0.25) |
| ANO6 | 5 | 35059738 | CTAT>C | 0/1 | c.2539 2541delATA | p.Ile847del | ENSBTAT00000003770.4 | - |
| ANO6 | 5 | 35059746 | T>C | 0/1 | c.2534A>G | p.Lys845Arg | ENSBTAT00000003770.4 | Tolerated (0.43) |
| ANO6 | 5 | 35059763 | T>C | 0/1 | c.2517A>G | p.Ile839Met | ENSBTAT00000003770.4 | Tolerated (0.23) |
| ANO6 | 5 | 35059767 | T>G | 0/1 | c.2513A>C | p.Asn838Thr | ENSBTAT00000003770.4 | Tolerated (0.05) |
| KIF21A | 5 | 42084854 | A>G | 0/1 | c.1351A>G | p.Thr451Ala | ENSBTAT00000006351.5 | Tolerated (1) |
| PTPRB | 5 | 43200476 | T>C | 0/1 | c.4754T>C | p.Phe1585Ser | ENSBTAT00000020345.5 | Tolerated (0.38) |
| ATP5B | 5 | 57120542 | G>A | 0/1 | c.142G>A | p.Ala48Thr | ENSBTAT00000017710.5 | Tolerated low confidence(0.32) |
| ATP5B | 5 | 57120548 | G>A | 0/1 | c.148G>A | p.Ala50Thr | ENSBTAT00000017710.5 | Tolerated low confidence(0.7) |
| ATP5B | 5 | 57120575 | A>G | 0/1 | c.175A>G | p.Thr59Ala | ENSBTAT00000017710.5 | Tolerated low confidence(1) |
| **Gene** | **BTA** | **Position** | **Base change** | **Genotype** | **cDNA** | **Protein** | **Transcript** | **SIFT** |
| BAZ2A | 5 | 57158692 | T>C | 0/1 | c.5533T>C | p.Ser1845Pro | ENSBTAT00000038215.4 | Tolerated (0.55) |
| RBMS2 | 5 | 57188394 | T>C | 0/1 | c.196A>G | p.Ser66Gly | ENSBTAT00000023705.3 | Tolerated (1) |
| TIMELESS | 5 | 57285638 | G>A | 0/1 | c.1363G>A | p.Val455Met | ENSBTAT00000027263.4 | Tolerated (0.42) |
| TIMELESS | 5 | 57285642 | G>C | 0/1 | c.1367G>C | p.Cys456Ser | ENSBTAT00000027263.4 | Tolerated (1) |
| ENSBTAG00000031097 | 5 | 59618637 | A>G | 0/1 | c.803T>C | p.Phe268Ser | ENSBTAT00000008283.5 | Deleterious low confidence(0.04) |
| ELFN2 | 5 | 76173878 | C>T | 0/1 | c.2092G>A | p.Val698Ile | ENSBTAT00000009548.5 | Tolerated (1) |
| ELFN2 | 5 | 76173885 | GCCACCACCACCGCTACTC>G | 0/1 | c.2067 2084delGAGTAGCGGTGGTGGTGG | p.Ser690 Gly695del | ENSBTAT00000009548.5 | - |
| ELFN2 | 5 | 76173908 | T>C | 0/1 | c.2062A>G | p.Ser688Gly | ENSBTAT00000009548.5 | Tolerated (1) |
| ELFN2 | 5 | 76173914 | T>C | 0/1 | c.2056A>G | p.Ser686Gly | ENSBTAT00000009548.5 | Tolerated (0.42) |
| FAR2 | 5 | 80702254 | T>C | 0/1 | c.322A>G | p.Ile108Val | ENSBTAT00000014725.5 | Tolerated (1) |
| FAR2 | 5 | 80702265 | T>C | 0/1 | c.311A>G | p.His104Arg | ENSBTAT00000014725.5 | Tolerated (0.53) |
| FAR2 | 5 | 80702266 | G>A | 0/1 | c.310C>T | p.His104Tyr | ENSBTAT00000014725.5 | Tolerated (0.07) |
| FAR2 | 5 | 80702281 | T>G | 0/1 | c.295A>C | p.Lys99Gln | ENSBTAT00000014725.5 | Tolerated (0.44) |
| FAR2 | 5 | 80702328 | G>T | 0/1 | c.248C>A | p.Ser83Tyr | ENSBTAT00000014725.5 | Tolerated (0.78) |
| CD163 | 5 | 102269892 | G>A | 0/1 | c.2233G>A | p.Asp745Asn | ENSBTAT00000026215.4 | Tolerated (0.57) |
| CD163 | 5 | 102269893 | A>G | 0/1 | c.2234A>G | p.Asp745Gly | ENSBTAT00000026215.4 | Tolerated (0.22) |
| NOP2 | 5 | 104221548 | G>A | 0/1 | c.1312G>A | p.Ala438Thr | ENSBTAT00000019607.4 | Tolerated (1) |
| NCAPD2 | 5 | 104247747 | A>G | 0/1 | c.3193T>C | p.Phe1065Leu | ENSBTAT00000019603.4 | Tolerated (1) |
| **Gene** | **BTA** | **Position** | **Base change** | **Genotype** | **cDNA** | **Protein** | **Transcript** | **SIFT** |
| FAM227A | 5 | 110782177 | G>C | 0/1 | c.763C>G | p.Leu255Val | ENSBTAT00000013979.5 | Tolerated (0.29) |
| MGAT3 | 5 | 111424803 | G>A | 0/1 | c.1517G>A | p.Arg506Gln | ENSBTAT00000016591.4 | Tolerated low confidence(0.55) |
| MGAT3 | 5 | 111424832 | T>C | 0/1 | c.1546T>C | p.Trp516Arg | ENSBTAT00000016591.4 | Tolerated low confidence(0.76) |
| MGAT3 | 5 | 111424842 | A>G | 0/1 | c.1556A>G | p.Lys519Arg | ENSBTAT00000016591.4 | Tolerated low confidence(1) |
| MGAT3 | 5 | 111424847 | T>C | 0/1 | c.1561T>C | p.Ser521Pro | ENSBTAT00000016591.4 | Tolerated low confidence(1) |
| TNRC6B | 5 | 112133269 | A>G | 0/1 | c.3238A>G | p.Ser1080Gly | ENSBTAT00000028072.5 | Tolerated (0.25) |
| SNU13 | 5 | 113257842 | A>G | 0/1 | c.88T>C | p.CysXArg | ENSBTAT00000008022.3 | Tolerated (0.24) |
| SNU13 | 5 | 113257852 | A>AAC | 0/1 | c.76 77dupGT | p.Gln27fs | ENSBTAT00000008022.3 | Deleterious (0) |
| SNU13 | 5 | 113257856 | A>C | 0/1 | c.74T>G | p.Leu25Arg | ENSBTAT00000008022.3 | Deleterious (0.03) |
| SNU13 | 5 | 113257857 | GA>G | 0/1 | c.72delT | p.Leu25fs | ENSBTAT00000008022.3 | - |
| SNU13 | 5 | 113257875 | T>G | 0/1 | c.55A>C | p.Thr19Pro | ENSBTAT00000008022.3 | - |
| PARVB | 5 | 115570938 | TC>T | 0/1 | c.838delC | p.His280fs | ENSBTAT00000029X7.5 | - |
| MAD2L1 | 6 | 6014242 | G>A | 0/1 | c.163G>A | p.Val55Ile | ENSBTAT00000049824.2 | Tolerated (0.06) |
| MAD2L1 | 6 | 6014243 | T>C | 0/1 | c.164T>C | p.Val55Ala | ENSBTAT00000049824.2 | Deleterious (0) |
| USP53 | 6 | 7168893 | T>G | 0/1 | c.1742A>C | p.Asn581Thr | ENSBTAT00000049054.3 | Tolerated (0.18) |
| USP53 | 6 | 7168905 | T>C | 0/1 | c.17XA>G | p.Asn577Ser | ENSBTAT00000049054.3 | Tolerated (1) |
| USP53 | 6 | 7168914 | T>C | 0/1 | c.1721A>G | p.Asn574Ser | ENSBTAT00000049054.3 | Tolerated (0.73) |
| **Gene** | **BTA** | **Position** | **Base change** | **Genotype** | **cDNA** | **Protein** | **Transcript** | **SIFT** |
| USP53 | 6 | 7168917 | G>T | 0/1 | c.1718C>A | p.Ser573Tyr | ENSBTAT00000049054.3 | Tolerated (0.83) |
| USP53 | 6 | 7168920 | C>G | 0/1 | c.1715G>C | p.Ser572Thr | ENSBTAT00000049054.3 | Tolerated (0.46) |
| USP53 | 6 | 7168936 | G>T | 0/1 | c.1699C>A | p.Arg567Ser | ENSBTAT00000049054.3 | Deleterious (0.03) |
| NDST3 | 6 | 8089705 | G>A | 0/1 | c.1864C>T | p.Leu622Phe | ENSBTAT00000064652.1 | Tolerated (0.07) |
| NDST4 | 6 | 11684671 | A>G | 0/1 | c.862A>G | p.Ile288Val | ENSBTAT00000049596.2 | Tolerated (0.83) |
| ADH5 | 6 | 26912539 | G>A | 0/1 | c.524G>A | p.Gly175Asp | ENSBTAT00000021X4.5 | Deleterious (0) |
| ADH5 | 6 | 26912571 | G>A | 0/1 | c.556G>A | p.Ala186Thr | ENSBTAT00000021X4.5 | Tolerated (1) |
| METAP1 | 6 | 26942977 | G>T | 0/1 | c.466C>A | p.Leu156Met | ENSBTAT00000021X3.5 | Tolerated (0.27) |
| LGI2 | 6 | 46128209 | GGT>G | 0/1 | c.1157 1158delAC | p.His386fs | ENSBTAT00000023124.3 | - |
| SEPSECS | 6 | 46255111 | G>C | 0/1 | c.1073C>G | p.Ala358Gly | ENSBTAT00000019464.3 | Tolerated (0.32) |
| SEPSECS | 6 | 46255112 | C>T | 0/1 | c.1072G>A | p.Ala358Thr | ENSBTAT00000019464.3 | Tolerated (0.48) |
| SEPSECS | 6 | 46255135 | T>C | 0/1 | c.1049A>G | p.Asn350Ser | ENSBTAT00000019464.3 | Tolerated (0.79) |
| SEPSECS | 6 | 46255154 | T>C | 0/1 | c.10XA>G | p.Met344Val | ENSBTAT00000019464.3 | Tolerated (0.41) |
| SLC34A2 | 6 | 46743432 | T>G | 0/1 | c.871T>G | p.Ser291Ala | ENSBTAT00000002023.5 | Tolerated (0.8) |
| SLC34A2 | 6 | 46743436 | T>C | 0/1 | c.875T>C | p.Val292Ala | ENSBTAT00000002023.5 | Tolerated (1) |
| SLC34A2 | 6 | 46743438 | C>A | 0/1 | c.877C>A | p.Gln293Lys | ENSBTAT00000002023.5 | Tolerated (0.85) |
| SLC34A2 | 6 | 46743450 | A>C | 0/1 | c.889A>C | p.Met297Leu | ENSBTAT00000002023.5 | Tolerated (1) |
| RBPJ | 6 | 47429076 | A>G | 0/1 | c.1444A>G | p.Thr482Ala | ENSBTAT00000004696.4 | Tolerated low confidence(0.34) |
| KLB | 6 | 60173951 | C>G | 0/1 | c.118C>G | p.Arg40Gly | ENSBTAT00000019297.5 | Tolerated (0.4) |
| **Gene** | **BTA** | **Position** | **Base change** | **Genotype** | **cDNA** | **Protein** | **Transcript** | **SIFT** |
| KLB | 6 | 60173960 | A>C | 0/1 | c.127A>C | p.Thr43Pro | ENSBTAT00000019297.5 | Tolerated (0.31) |
| KLB | 6 | 60173984 | A>G | 0/1 | c.151A>G | p.Thr51Ala | ENSBTAT00000019297.5 | Tolerated (1) |
| KLB | 6 | 60193984 | C>G | 0/1 | c.1122C>G | p.His374Gln | ENSBTAT00000019297.5 | Tolerated (0.34) |
| KLB | 6 | 60205865 | A>G | 0/1 | c.2743A>G | p.Ile915Val | ENSBTAT00000019297.5 | Tolerated (1) |
| SRD5A3 | 6 | 72518048 | C>A | 0/1 | c.823C>A | p.Leu275Met | ENSBTAT00000019855.5 | Tolerated (0.47) |
| SRD5A3 | 6 | 72518084 | T>C | 0/1 | c.859T>C | p.Tyr287His | ENSBTAT00000019855.5 | Tolerated (1) |
| AASDH | 6 | 73422475 | T>C | 0/1 | c.1171A>G | p.Asn391Asp | ENSBTAT00000027426.4 | Tolerated (0.28) |
| TECRL | 6 | 81512110 | T>C | 0/1 | c.1027A>G | p.Ile343Val | ENSBTAT00000034572.2 | Tolerated (0.54) |
| TECRL | 6 | 81512125 | T>G | 0/1 | c.1012A>C | p.Lys338Gln | ENSBTAT00000034572.2 | Tolerated (0.36) |
| ADAMTS3 | 6 | 89438485 | C>T | 0/1 | c.337G>A | p.Ala113Thr | ENSBTAT00000061447.2 | Tolerated (0.61) |
| SHROOM3 | 6 | 93364787 | G>A | 0/1 | c.326G>A | p.Arg109Gln | ENSBTAT00000026154.5 | Tolerated (1) |
| WFS1 | 6 | 104674210 | G>T | 0/1 | c.2158C>A | p.His720Asn | ENSBTAT00000066249.1 | Tolerated (1) |
| WFS1 | 6 | 104674213 | C>T | 0/1 | c.2155G>A | p.Val719Ile | ENSBTAT00000066249.1 | Tolerated (1) |
| DRD5 | 6 | 107576740 | G>T | 0/1 | c.1223G>T | p.Arg408Leu | ENSBTAT00000056172.2 | Tolerated (1) |
| DRD5 | 6 | 107576770 | C>G | 0/1 | c.1253C>G | p.Ala418Gly | ENSBTAT00000056172.2 | Tolerated (0.39) |
| ADD1 | 6 | 107949391 | T>C | 0/1 | c.850A>G | p.Ile284Val | ENSBTAT00000028152.4 | Tolerated (1) |
| WHSC1 | 6 | 109826766 | CT>C | 0/1 | c.3952delT | p.Ser1318fs | ENSBTAT00000010497.4 | - |
| SLC25A42 | 7 | 4055410 | C>G | 0/1 | c.261G>C | p.Leu87Phe | ENSBTAT00000002374.4 | Tolerated (0.55) |
| SLC25A42 | 7 | 4055424 | G>T | 0/1 | c.247C>A | p.His83Asn | ENSBTAT00000002374.4 | Tolerated (0.74) |
| **Gene** | **BTA** | **Position** | **Base change** | **Genotype** | **cDNA** | **Protein** | **Transcript** | **SIFT** |
| MAP1S | 7 | 5335772 | T>C | 0/1 | c.3167A>G | p.Asp1056Gly | ENSBTAT00000019513.4 | Tolerated (0.08) |
| MAP1S | 7 | 5335806 | C>G | 0/1 | c.3133G>C | p.Val1045Leu | ENSBTAT00000019513.4 | Deleterious (0) |
| MAP1S | 7 | 5336911 | T>C | 0/1 | c.2948A>G | p.Tyr983Cys | ENSBTAT00000019513.4 | Deleterious (0) |
| MAP1S | 7 | 5336914 | C>T | 0/1 | c.2945G>A | p.Cys982Tyr | ENSBTAT00000019513.4 | Tolerated (0.57) |
| MYO9B | 7 | 5891914 | C>T | 0/1 | c.254G>A | p.Ser85Asn | ENSBTAT00000014779.3 | Tolerated (0.93) |
| CACNA1A | 7 | 13419670 | A>G | 0/1 | c.2623A>G | p.Ser875Gly | ENSBTAT00000019752.5 | Tolerated (1) |
| CALR | 7 | 13734007 | A>C | 0/1 | c.663T>G | p.Asp221Glu | ENSBTAT00000020111.2 | Tolerated (0.52) |
| CALR | 7 | 137340X | C>A | 0/1 | c.640G>T | p.Ala214Ser | ENSBTAT00000020111.2 | Tolerated (0.2) |
| ZNF699 | 7 | 15X2386 | T>C | 0/1 | c.1618A>G | p.Thr540Ala | ENSBTAT0000005X09.2 | Tolerated (0.77) |
| DNMT1 | 7 | 15947742 | G>A | 0/1 | c.382C>T | p.Pro128Ser | ENSBTAT00000003549.5 | Tolerated low confidence(1) |
| DNMT1 | 7 | 15947746 | GCC>G | 0/1 | c.376 377delGG | p.Gly126fs | ENSBTAT00000003549.5 | - |
| DNMT1 | 7 | 15947751 | T>TTC | 0/1 | c.372 373insGA | p.Lys125fs | ENSBTAT00000003549.5 | - |
| DNMT1 | 7 | 15947762 | A>C | 0/1 | c.362T>G | p.Val121Gly | ENSBTAT00000003549.5 | Tolerated low confidence(0.39) |
| DNMT1 | 7 | 15947772 | A>G | 0/1 | c.352T>C | p.Cys118Arg | ENSBTAT00000003549.5 | Tolerated low confidence(0.73) |
| SLC44A2 | 7 | 16354107 | T>C | 0/1 | c.1900T>C | p.Ser634Pro | ENSBTAT00000003403.4 | Tolerated (0.45) |
| PRAM1 | 7 | 18331816 | C>T | 0/1 | c.1831G>A | p.Val611Ile | ENSBTAT00000045X1.3 | Tolerated (1) |
| TUBB4A | 7 | 19211299 | T>G | 0/1 | c.1153T>G | p.Phe385Val | ENSBTAT00000014560.3 | Deleterious low confidence(0) |
| **Gene** | **BTA** | **Position** | **Base change** | **Genotype** | **cDNA** | **Protein** | **Transcript** | **SIFT** |
| TUBB4A | 7 | 19211353 | A>G | 0/1 | c.1207A>G | p.Met403Val | ENSBTAT00000014560.3 | Tolerated low confidence(0.05) |
| FBN2 | 7 | 26894273 | CG>C | 0/1 | c.8816delG | p.Gly2939fs | ENSBTAT00000020360.5 | - |
| PHAX | 7 | 28565413 | C>T | 0/1 | c.686G>A | p.Arg229Lys | ENSBTAT00000035608.2 | Tolerated (1) |
| DMXL1 | 7 | 36058895 | G>C | 0/1 | c.2773C>G | p.Leu925Val | ENSBTAT00000065277.1 | Tolerated (0.61) |
| NSD1 | 7 | 40052886 | A>G | 0/1 | c.3542A>G | p.Lys1181Arg | ENSBTAT00000034204.4 | Tolerated low confidence(1) |
| NSD1 | 7 | 40052927 | T>C | 0/1 | c.3583T>C | p.Ser1195Pro | ENSBTAT00000034204.4 | Tolerated low confidence(1) |
| NSD1 | 7 | 400529X | G>T | 0/1 | c.3586G>T | p.Ala1196Ser | ENSBTAT00000034204.4 | Tolerated low confidence(0.94) |
| ENSBTAG00000046034 | 7 | 40468340 | G>A | 0/1 | c.842C>T | p.Pro281Leu | ENSBTAT00000062946.1 | Deleterious low confidence(0.01) |
| OR6F1 | 7 | 42479969 | A>G | 0/1 | c.290A>G | p.Lys97Arg | ENSBTAT00000056844.2 | Tolerated (0.66) |
| OR6F1 | 7 | 42479978 | G>C | 0/1 | c.299G>C | p.Ser100Thr | ENSBTAT00000056844.2 | Tolerated (1) |
| OR6F1 | 7 | 42479983 | T>A | 0/1 | c.X4T>A | p.Ser102Thr | ENSBTAT00000056844.2 | Deleterious (0.04) |
| OR6F1 | 7 | 42479992 | A>G | 0/1 | c.313A>G | p.Ser105Gly | ENSBTAT00000056844.2 | Tolerated (1) |
| ENSBTAG00000046474 | 7 | 42914076 | C>A | 0/1 | c.245C>A | p.Ser82Tyr | ENSBTAT00000066287.1 | Tolerated (1) |
| ENSBTAG00000046474 | 7 | 42914084 | C>G | 0/1 | c.253C>G | p.Leu85Val | ENSBTAT00000066287.1 | Deleterious (0.03) |
| AFF4 | 7 | 46103659 | C>T | 0/1 | c.1726G>A | p.Ala576Thr | ENSBTAT00000012480.5 | Tolerated (1) |
| SEC24A | 7 | 47793186 | T>G | 0/1 | c.1024T>G | p.Ser342Ala | ENSBTAT00000043111.2 | Tolerated (1) |
| MYOZ3 | 7 | 64033619 | C>T | 0/1 | c.25C>T | p.Pro9Ser | ENSBTAT00000035177.4 | Tolerated (0.08) |
| **Gene** | **BTA** | **Position** | **Base change** | **Genotype** | **cDNA** | **Protein** | **Transcript** | **SIFT** |
| MYOZ3 | 7 | 64033628 | A>G | 0/1 | c.34A>G | p.Thr12Ala | ENSBTAT00000035177.4 | Tolerated (1) |
| MYOZ3 | 7 | 64033631 | A>G | 0/1 | c.37A>G | p.Thr13Ala | ENSBTAT00000035177.4 | Tolerated (0.72) |
| FAT2 | 7 | 64753939 | T>A | 0/1 | c.3743A>T | p.Tyr1248Phe | ENSBTAT00000061431.2 | Tolerated (1) |
| FAT2 | 7 | 64774192 | G>C | 0/1 | c.500G>C | p.Ser167Thr | ENSBTAT00000004553.5 | Tolerated (1) |
| TENM2 | 7 | 82563354 | A>G | 0/1 | c.4600A>G | p.Ile1534Val | ENSBTAT000000350X.4 | Tolerated (1) |
| KIAA0825 | 7 | 96287995 | G>T | 0/1 | c.72C>A | p.Asp24Glu | ENSBTAT00000000923.5 | Tolerated (0.23) |
| ERAP2 | 7 | 987X494 | G>T | 0/1 | c.892G>T | p.Val298Leu | ENSBTAT00000001164.5 | Tolerated (0.28) |
| ERAP2 | 7 | 987X506 | G>A | 0/1 | c.904G>A | p.AspX2Asn | ENSBTAT00000001164.5 | Deleterious (0.02) |
| FZD3 | 8 | 10058063 | T>C | 0/1 | c.334A>G | p.Lys112Glu | ENSBTAT00000061597.2 | Tolerated (0.36) |
| NDUFB6 | 8 | 11373149 | C>G | 0/1 | c.122C>G | p.Ser41Trp | ENSBTAT00000007761.2 | Tolerated (0.32) |
| NDUFB6 | 8 | 11373154 | G>A | 0/1 | c.127G>A | p.Val43Met | ENSBTAT00000007761.2 | Tolerated (0.47) |
| ENSBTAG00000025929 | 8 | 222X780 | C>G | 0/1 | c.594G>C | p.Gln198His | ENSBTAT00000048509.3 | Deleterious (0.03) |
| ENSBTAG00000025929 | 8 | 222X781 | T>C | 0/1 | c.593A>G | p.Gln198Arg | ENSBTAT00000048509.3 | Tolerated (1) |
| ENSBTAG00000025929 | 8 | 222X791 | T>A | 0/1 | c.583A>T | p.Ile195Phe | ENSBTAT00000048509.3 | Tolerated (0.71) |
| PLIN2 | 8 | 25137124 | A>G | 0/1 | c.1031A>G | p.Asn344Ser | ENSBTAT00000007519.3 | Tolerated (0.6) |
| MPDZ | 8 | 31217041 | C>A | 0/1 | c.3262C>A | p.Leu1088Met | ENSBTAT00000061015.2 | Tolerated (1) |
| ENSBTAG00000047550 | 8 | 39038267 | G>A | 0/1 | c.1138G>A | p.Ala380Thr | ENSBTAT00000063356.1 | Tolerated (1) |
| ENSBTAG00000047550 | 8 | 39038X1 | C>T | 0/1 | c.1172C>T | p.Ala391Val | ENSBTAT00000063356.1 | Tolerated (0.14) |
| C9orf135 | 8 | 46235541 | A>C | 0/1 | c.79A>C | p.Met27Leu | ENSBTAT00000047514.3 | Tolerated (1) |
| **Gene** | **BTA** | **Position** | **Base change** | **Genotype** | **cDNA** | **Protein** | **Transcript** | **SIFT** |
| C9orf135 | 8 | 46235561 | A>C | 0/1 | c.99A>C | p.Leu33Phe | ENSBTAT00000047514.3 | Deleterious (0) |
| C9orf135 | 8 | 46235563 | C>G | 0/1 | c.101C>G | p.Thr34Ser | ENSBTAT00000047514.3 | Tolerated (0.22) |
| ENSBTAG00000047788 | 8 | 53599792 | A>C | 0/1 | c.854A>C | p.Lys285Thr | ENSBTAT00000064443.1 | Tolerated (0.37) |
| ENSBTAG00000047788 | 8 | 53599815 | A>G | 0/1 | c.877A>G | p.Lys293Glu | ENSBTAT00000064443.1 | Tolerated (0.85) |
| ENSBTAG00000047788 | 8 | 53599822 | C>A | 0/1 | c.884C>A | p.Pro295Gln | ENSBTAT00000064443.1 | Tolerated (1) |
| ENSBTAG00000047788 | 8 | 53599825 | C>G | 0/1 | c.887C>G | p.Thr296Ser | ENSBTAT00000064443.1 | Tolerated (0.58) |
| ENSBTAG00000047788 | 8 | 53599839 | A>G | 0/1 | c.901A>G | p.IleX1Val | ENSBTAT00000064443.1 | Tolerated (1) |
| ENSBTAG00000011402 | 8 | 60072214 | A>G | 0/1 | c.807A>G | p.Ile269Met | ENSBTAT00000015155.4 | Tolerated (0.09) |
| ENSBTAG00000011402 | 8 | 60072224 | A>T | 0/1 | c.817A>T | p.Met273Leu | ENSBTAT00000015155.4 | Tolerated (0.23) |
| DOK2 | 8 | 69725608 | G>T | 0/1 | c.835C>A | p.Pro279Thr | ENSBTAT00000004337.5 | Deleterious (0.02) |
| NTRK2 | 8 | 79401693 | C>G | 0/1 | c.656C>G | p.Thr219Ser | ENSBTAT00000061120.2 | Tolerated (1) |
| ZCCHC6 | 8 | 80899520 | C>T | 0/1 | c.166G>A | p.Val56Ile | ENSBTAT00000002277.5 | Tolerated (0.58) |
| ENSBTAG00000009903 | 8 | 83543176 | C>T | 0/1 | c.382G>A | p.Ala128Thr | ENSBTAT0000001X74.4 | Tolerated low confidence(1) |
| CDC14B | 8 | 84656405 | A>G | 0/1 | c.619T>C | p.Tyr207His | ENSBTAT00000015397.4 | Tolerated (1) |
| CDC14B | 8 | 84656441 | A>G | 0/1 | c.583T>C | p.Phe195Leu | ENSBTAT00000015397.4 | Tolerated (0.96) |
| CDC14B | 8 | 84656444 | A>G | 0/1 | c.580T>C | p.Phe194Leu | ENSBTAT00000015397.4 | Deleterious (0) |
| CDC14B | 8 | 84656446 | C>T | 0/1 | c.578G>A | p.Gly193Asp | ENSBTAT00000015397.4 | Deleterious (0.03) |
| CTSL2 | 8 | 84979261 | T>C | 0/1 | c.364A>G | p.Lys122Glu | ENSBTAT00000022710.3 | Tolerated (0.99) |
| CTSL2 | 8 | 84979263 | G>C | 0/1 | c.362C>G | p.Thr121Ser | ENSBTAT00000022710.3 | Tolerated (0.99) |
| **Gene** | **BTA** | **Position** | **Base change** | **Genotype** | **cDNA** | **Protein** | **Transcript** | **SIFT** |
| CTSL2 | 8 | 84979285 | C>T | 0/1 | c.340G>A | p.Val114Ile | ENSBTAT00000022710.3 | Deleterious (0.01) |
| CTSL2 | 8 | 84979286 | G>C | 0/1 | c.339C>G | p.Asp113Glu | ENSBTAT00000022710.3 | Deleterious (0.01) |
| CTSL2 | 8 | 84979290 | A>G | 0/1 | c.335T>C | p.Val112Ala | ENSBTAT00000022710.3 | Tolerated (0.74) |
| CTSL2 | 8 | 84979294 | G>A | 0/1 | c.331C>T | p.Leu111Phe | ENSBTAT00000022710.3 | Tolerated (0.74) |
| CTSL2 | 8 | 84979X4 | A>T | 0/1 | c.321T>A | p.His107Gln | ENSBTAT00000022710.3 | Tolerated (1) |
| CTSL2 | 8 | 84979312 | G>T | 0/1 | c.313C>A | p.Leu105Met | ENSBTAT00000022710.3 | Tolerated (1) |
| ROR2 | 8 | 87574798 | A>C | 0/1 | c.1364A>C | p.Asn455Thr | ENSBTAT00000061589.2 | Tolerated (0.13) |
| TMEM246 | 8 | 92828428 | A>C | 0/1 | c.964T>G | p.Ser322Ala | ENSBTAT00000027776.5 | Tolerated (1) |
| GRIN3A | 8 | 9X87376 | C>T | 0/1 | c.1249G>A | p.Val417Met | ENSBTAT00000024549.5 | Tolerated (0.37) |
| OR13C8 | 8 | 96092863 | T>A | 0/1 | c.490T>A | p.Leu164Met | ENSBTAT00000050504.3 | Tolerated (1) |
| ACTL7B | 8 | 100139146 | T>C | 0/1 | c.578A>G | p.Lys193Arg | ENSBTAT00000025932.5 | Tolerated (1) |
| ACTL7B | 8 | 100139174 | A>G | 0/1 | c.550T>C | p.Ser184Pro | ENSBTAT00000025932.5 | Tolerated (0.07) |
| ACTL7B | 8 | 100139180 | A>G | 0/1 | c.544T>C | p.Ser182Pro | ENSBTAT00000025932.5 | Deleterious (0.04) |
| ACTL7B | 8 | 100139192 | T>G | 0/1 | c.532A>C | p.Met178Leu | ENSBTAT00000025932.5 | Tolerated (0.1) |
| ACTL7B | 8 | 100139201 | T>C | 0/1 | c.523A>G | p.Ile175Val | ENSBTAT00000025932.5 | Tolerated (1) |
| ACTL7B | 8 | 100139205 | G>C | 0/1 | c.519C>G | p.Phe173Leu | ENSBTAT00000025932.5 | Tolerated (0.07) |
| ACTL7B | 8 | 100139210 | T>C | 0/1 | c.514A>G | p.Thr172Ala | ENSBTAT00000025932.5 | Tolerated (0.13) |
| ACTL7B | 8 | 100139241 | G>C | 0/1 | c.483C>G | p.Asn161Lys | ENSBTAT00000025932.5 | Deleterious (0) |
| ACTL7B | 8 | 100139243 | T>G | 0/1 | c.481A>C | p.Asn161His | ENSBTAT00000025932.5 | Deleterious (0.04) |
| **Gene** | **BTA** | **Position** | **Base change** | **Genotype** | **cDNA** | **Protein** | **Transcript** | **SIFT** |
| ACTL7B | 8 | 100139246 | T>C | 0/1 | c.478A>G | p.Ser160Gly | ENSBTAT00000025932.5 | Tolerated (0.09) |
| ACTL7B | 8 | 100139249 | T>C | 0/1 | c.475A>G | p.Thr159Ala | ENSBTAT00000025932.5 | Tolerated (0.07) |
| ACTL7B | 8 | 100139255 | T>C | 0/1 | c.469A>G | p.Ser157Gly | ENSBTAT00000025932.5 | Tolerated (0.07) |
| ACTL7B | 8 | 100139273 | C>G | 0/1 | c.451G>C | p.Val151Leu | ENSBTAT00000025932.5 | Tolerated (1) |
| MUSK | 8 | 101904778 | T>A | 0/1 | c.451T>A | p.Ser151Thr | ENSBTAT00000065708.1 | Tolerated (0.47) |
| PTP4A1 | 9 | 512432 | G>A | 0/1 | c.44G>A | p.Arg15Lys | ENSBTAT00000002933.5 | Tolerated (1) |
| ADGRB3 | 9 | 7946272 | GC>G | 0/1 | c.465delC | p.Ser156fs | ENSBTAT00000063222.1 | - |
| ADGRB3 | 9 | 7946276 | C>CA | 0/1 | c.468 469insA | p.Trp157fs | ENSBTAT00000063222.1 | - |
| ADGRB3 | 9 | 7946293 | C>T | 0/1 | c.485C>T | p.Ala162Val | ENSBTAT00000063222.1 | Tolerated (1) |
| ADGRB3 | 9 | 7946329 | T>A | 0/1 | c.521T>A | p.Leu174Gln | ENSBTAT00000063222.1 | Tolerated (0.33) |
| COL12A1 | 9 | 14912067 | C>T | 0/1 | c.6511G>A | p.Val2171Met | ENSBTAT00000026725.5 | Tolerated (0.09) |
| COL12A1 | 9 | 14912081 | A>G | 0/1 | c.6497T>C | p.Val2166Ala | ENSBTAT00000026725.5 | Tolerated (0.42) |
| LCA5 | 9 | 19328026 | C>A | 0/1 | c.1525G>T | p.Gly509Trp | ENSBTAT00000061609.2 | Deleterious (0) |
| ENSBTAG00000038849 | 9 | 28154816 | G>C | 0/1 | c.351G>C | p.Glu117Asp | ENSBTAT00000056240.2 | Tolerated (1) |
| ENSBTAG00000038849 | 9 | 28154822 | TGAA>T | 0/1 | c.363 365delAGA | p.Glu121del | ENSBTAT00000056240.2 | - |
| ENSBTAG00000038849 | 9 | 28154846 | G>T | 0/1 | c.381G>T | p.Glu127Asp | ENSBTAT00000056240.2 | Tolerated (1) |
| CEP85L | 9 | 32867687 | C>G | 0/1 | c.1208C>G | p.Thr403Ser | ENSBTAT00000017179.4 | Tolerated (0.34) |
| CEP85L | 9 | 32867720 | C>T | 0/1 | c.1241C>T | p.Ala414Val | ENSBTAT00000017179.4 | Tolerated (0.35) |
| PRDM1 | 9 | 44357881 | T>C | 0/1 | c.2263A>G | p.Met755Val | ENSBTAT00000001081.5 | Tolerated (1) |
| **Gene** | **BTA** | **Position** | **Base change** | **Genotype** | **cDNA** | **Protein** | **Transcript** | **SIFT** |
| PRDM1 | 9 | 44357886 | ATGT>A | 0/1 | c.2255 2257delACA | p.Asn752del | ENSBTAT00000001081.5 | - |
| USP45 | 9 | 50986179 | G>A | 0/1 | c.727G>A | p.Val243Met | ENSBTAT00000037865.2 | Tolerated (0.2) |
| KLHL32 | 9 | 53392719 | A>G | 0/1 | c.727T>C | p.Cys243Arg | ENSBTAT00000006977.4 | Deleterious (0.05) |
| GABRR1 | 9 | 61832383 | G>C | 0/1 | c.684G>C | p.Leu228Phe | ENSBTAT00000015496.4 | Deleterious (0) |
| CYB5R4 | 9 | 6650X67 | A>G | 0/1 | c.53T>C | p.Val18Ala | ENSBTAT00000054027.1 | Tolerated (0.14) |
| STXBP5 | 9 | 85765448 | A>G | 0/1 | c.2422A>G | p.Thr808Ala | ENSBTAT00000061280.2 | Tolerated (0.25) |
| STXBP5 | 9 | 85765457 | G>T | 0/1 | c.2431G>T | p.Ala811Ser | ENSBTAT00000061280.2 | Tolerated (0.55) |
| SYNE1 | 9 | 90X4913 | T>A | 0/1 | c.24825A>T | p.Glu8275Asp | ENSBTAT00000012328.5 | Tolerated (0.07) |
| SYNE1 | 9 | 90X4917 | G>T | 0/1 | c.24821C>A | p.Pro8274Gln | ENSBTAT00000012328.5 | Tolerated (0.52) |
| SYNE1 | 9 | 90487178 | G>T | 0/1 | c.15515C>A | p.Thr5172Lys | ENSBTAT00000012328.5 | - |
| SYNE1 | 9 | 90487206 | A>T | 0/1 | c.15487T>A | p.Leu5163Ile | ENSBTAT00000012328.5 | - |
| CCR6 | 9 | 103461431 | C>CGAGG | 0/1 | c.592 593insAGGG | p.Ala198fs | ENSBTAT00000037034.4 | - |
| CCR6 | 9 | 103461433 | CCGTG>C | 0/1 | c.594 597delCGTG | p.Val199fs | ENSBTAT00000037034.4 | - |
| CCR6 | 9 | 103461441 | G>A | 0/1 | c.601G>A | p.Glu201Lys | ENSBTAT00000037034.4 | Tolerated (0.5) |
| CCR6 | 9 | 103461450 | C>A | 0/1 | c.610C>A | p.Arg204Ser | ENSBTAT00000037034.4 | Tolerated (0.71) |
| CCR6 | 9 | 103461451 | G>A | 0/1 | c.611G>A | p.Arg204His | ENSBTAT00000037034.4 | Tolerated (0.15) |
| AP3B1 | 10 | 9146110 | C>T | 0/1 | c.1714G>A | p.Asp572Asn | ENSBTAT00000006605.4 | Deleterious (0) |
| DMGDH | 10 | 10031189 | C>T | 0/1 | c.1780G>A | p.Val594Ile | ENSBTAT00000036611.4 | Tolerated (1) |
| DMGDH | 10 | 10031210 | A>T | 0/1 | c.1759T>A | p.Ser587Thr | ENSBTAT00000036611.4 | Tolerated (0.59) |
| **Gene** | **BTA** | **Position** | **Base change** | **Genotype** | **cDNA** | **Protein** | **Transcript** | **SIFT** |
| IGDCC4 | 10 | 12278627 | T>G | 0/1 | c.897A>C | p.Arg299Ser | ENSBTAT00000008442.4 | Deleterious (0) |
| CD276 | 10 | 20346452 | G>C | 0/1 | c.1176G>C | p.Leu392Phe | ENSBTAT00000026X0.5 | Tolerated (0.57) |
| DHRS1 | 10 | 20707147 | G>A | 0/1 | c.215G>A | p.Arg72Gln | ENSBTAT00000013522.3 | Tolerated (0.31) |
| DHRS1 | 10 | 20707150 | A>G | 0/1 | c.218A>G | p.Asn73Ser | ENSBTAT00000013522.3 | Tolerated (0.76) |
| DHRS1 | 10 | 20707151 | C>G | 0/1 | c.219C>G | p.Asn73Lys | ENSBTAT00000013522.3 | Tolerated (0.93) |
| DHRS1 | 10 | 20707171 | G>A | 0/1 | c.239G>A | p.Arg80Gln | ENSBTAT00000013522.3 | Tolerated (0.52) |
| MYH7 | 10 | 21329671 | T>A | 0/1 | c.1097T>A | p.Leu366Gln | ENSBTAT00000061X6.2 | Tolerated (1) |
| METTL3 | 10 | 25729061 | TTG>T | 0/1 | c.1175 1176delTG | p.Val392fs | ENSBTAT00000020151.4 | - |
| OR11H7 | 10 | 26917567 | C>T | 0/1 | c.96G>A | p.Met32Ile | ENSBTAT00000002208.5 | Tolerated (1) |
| ENSBTAG00000038672 | 10 | 27190719 | A>C | 0/1 | c.139T>G | p.Cys47Gly | ENSBTAT00000055011.2 | Deleterious (0.01) |
| ENSBTAG00000038672 | 10 | 27190738 | T>C | 0/1 | c.120A>G | p.Ile40Met | ENSBTAT00000055011.2 | Tolerated (1) |
| ENSBTAG00000038672 | 10 | 27190743 | G>C | 0/1 | c.115C>G | p.Leu39Val | ENSBTAT00000055011.2 | Tolerated (0.06) |
| ANKRD63 | 10 | 36017617 | A>T | 0/1 | c.748T>A | p.Cys250Ser | ENSBTAT00000064185.1 | Tolerated low confidence(0.91) |
| ANKRD63 | 10 | 36017626 | G>C | 0/1 | c.739C>G | p.Pro247Ala | ENSBTAT00000064185.1 | Tolerated low confidence(1) |
| ANKRD63 | 10 | 36017639 | G>C | 0/1 | c.726C>G | p.Phe242Leu | ENSBTAT00000064185.1 | Tolerated low confidence(0.37) |
| ANKRD63 | 10 | 36017640 | A>G | 0/1 | c.725T>C | p.Phe242Ser | ENSBTAT00000064185.1 | Tolerated low confidence(0.2) |
| SOS2 | 10 | 4X96822 | T>G | 0/1 | c.3762A>C | p.Gln1254His | ENSBTAT00000007299.5 | Tolerated low confidence(0.73) |
| **Gene** | **BTA** | **Position** | **Base change** | **Genotype** | **cDNA** | **Protein** | **Transcript** | **SIFT** |
| SOS2 | 10 | 4X96839 | C>G | 0/1 | c.3745G>C | p.Val1249Leu | ENSBTAT00000007299.5 | Tolerated low confidence(0.54) |
| SOS2 | 10 | 4X96967 | G>T | 0/1 | c.3617C>A | p.Thr1206Asn | ENSBTAT00000007299.5 | Tolerated low confidence(1) |
| NIN | 10 | 43672476 | T>C | 0/1 | c.3515A>G | p.Lys1172Arg | ENSBTAT00000061216.2 | Tolerated (0.3) |
| NIN | 10 | 43672480 | T>C | 0/1 | c.3511A>G | p.Ile1171Val | ENSBTAT00000061216.2 | Tolerated (0.33) |
| NIN | 10 | 43672486 | T>C | 0/1 | c.3505A>G | p.Lys1169Glu | ENSBTAT00000061216.2 | Tolerated (0.27) |
| RAB8B | 10 | 46847068 | ATGC>A | 0/1 | c.325-1 326delGCA | p.His109fs | ENSBTAT00000014802.4 | - |
| NEDD4 | 10 | 54537074 | A>T | 0/1 | c.538A>T | p.Asn180Tyr | ENSBTAT00000018331.5 | Deleterious low confidence(0) |
| NEDD4 | 10 | 54537081 | A>T | 0/1 | c.545A>T | p.Asn182Ile | ENSBTAT00000018331.5 | Tolerated low confidence(0.48) |
| WDR72 | 10 | 5669X75 | G>T | 0/1 | c.355G>T | p.Ala119Ser | ENSBTAT00000061231.2 | Tolerated (0.54) |
| WDR72 | 10 | 5669X76 | C>T | 0/1 | c.356C>T | p.Ala119Val | ENSBTAT00000061231.2 | Tolerated (0.16) |
| WDR72 | 10 | 5669X86 | A>AGGG | 0/1 | c.367 368insGGG | p.Thr122 Glu123insGly | ENSBTAT00000061231.2 | - |
| WDR72 | 10 | 56693151 | T>C | 0/1 | c.431T>C | p.Val144Ala | ENSBTAT00000061231.2 | Tolerated (0.69) |
| WDR72 | 10 | 56693153 | A>G | 0/1 | c.433A>G | p.Ile145Val | ENSBTAT00000061231.2 | Tolerated (1) |
| WDR72 | 10 | 56693168 | G>T | 0/1 | c.448G>T | p.Val150Leu | ENSBTAT00000061231.2 | Tolerated (0.48) |
| MYO5A | 10 | 58096645 | G>A | 0/1 | c.2648G>A | p.Cys883Tyr | ENSBTAT00000008516.5 | Tolerated (0.2) |
| MYO5A | 10 | 58096661 | C>G | 0/1 | c.2664C>G | p.Ile888Met | ENSBTAT00000008516.5 | Tolerated (0.18) |
| TRPM7 | 10 | 59901756 | A>T | 0/1 | c.2640A>T | p.Glu880Asp | ENSBTAT00000044081.3 | Tolerated (0.3) |
| **Gene** | **BTA** | **Position** | **Base change** | **Genotype** | **cDNA** | **Protein** | **Transcript** | **SIFT** |
| TRPM7 | 10 | 59901769 | G>A | 0/1 | c.2653G>A | p.Val885Ile | ENSBTAT00000044081.3 | Tolerated (0.25) |
| USP8 | 10 | 59984941 | T>C | 0/1 | c.3241A>G | p.Thr1081Ala | ENSBTAT00000033814.4 | Tolerated low confidence(0.72) |
| USP8 | 10 | 59984943 | A>G | 0/1 | c.3239T>C | p.Ile1080Thr | ENSBTAT00000033814.4 | Tolerated low confidence(0.66) |
| USP8 | 10 | 59984944 | T>C | 0/1 | c.3238A>G | p.Ile1080Val | ENSBTAT00000033814.4 | Tolerated low confidence(0.19) |
| SLC12A1 | 10 | 62335595 | C>T | 0/1 | c.2635G>A | p.Gly879Ser | ENSBTAT00000046583.3 | Tolerated (0.71) |
| SLC12A1 | 10 | 62335640 | T>A | 0/1 | c.2590A>T | p.Ser864Cys | ENSBTAT00000046583.3 | Tolerated (0.13) |
| SLC12A1 | 10 | 62335658 | T>C | 0/1 | c.2572A>G | p.Thr858Ala | ENSBTAT00000046583.3 | Tolerated (0.59) |
| WDHD1 | 10 | 67720598 | A>C | 0/1 | c.460T>G | p.Ser154Ala | ENSBTAT00000025453.5 | Tolerated (1) |
| LGALS3 | 10 | 67860829 | A>T | 0/1 | c.660A>T | p.Glu220Asp | ENSBTAT00000043753.2 | Deleterious (0.03) |
| LGALS3 | 10 | 678608X | C>T | 0/1 | c.661C>T | p.Pro221Ser | ENSBTAT00000043753.2 | Tolerated (0.81) |
| LGALS3 | 10 | 67860855 | A>G | 0/1 | c.686A>G | p.Asn229Ser | ENSBTAT00000043753.2 | Deleterious (0) |
| LGALS3 | 10 | 67860861 | C>T | 0/1 | c.692C>T | p.Ala231Val | ENSBTAT00000043753.2 | Deleterious (0.03) |
| ARID4A | 10 | 70855021 | T>C | 0/1 | c.545T>C | p.Val182Ala | ENSBTAT00000025155.5 | Tolerated (0.74) |
| ARID4A | 10 | 70855039 | G>C | 0/1 | c.563G>C | p.Ser188Thr | ENSBTAT00000025155.5 | Tolerated (1) |
| ARID4A | 10 | 70855043 | A>T | 0/1 | c.567A>T | p.Glu189Asp | ENSBTAT00000025155.5 | Tolerated (0.62) |
| PCNX4 | 10 | 72584116 | A>G | 0/1 | c.1258A>G | p.Ile420Val | ENSBTAT00000018737.5 | Tolerated (1) |
| PCNX4 | 10 | 72584134 | T>G | 0/1 | c.1276T>G | p.Phe426Val | ENSBTAT00000018737.5 | Tolerated (0.08) |
| **Gene** | **BTA** | **Position** | **Base change** | **Genotype** | **cDNA** | **Protein** | **Transcript** | **SIFT** |
| PCNX4 | 10 | 72584148 | C>G | 0/1 | c.1290C>G | p.Phe4XLeu | ENSBTAT00000018737.5 | Tolerated (0.81) |
| PCNX4 | 10 | 72584198 | A>G | 0/1 | c.1340A>G | p.Lys447Arg | ENSBTAT00000018737.5 | Tolerated (0.82) |
| PCNX4 | 10 | 72584203 | A>G | 0/1 | c.1345A>G | p.Met449Val | ENSBTAT00000018737.5 | Tolerated (0.51) |
| ENSBTAG00000046X9 | 10 | 73133365 | C>T | 0/1 | c.478G>A | p.Ala160Thr | ENSBTAT00000065236.1 | Tolerated (1) |
| HIF1A | 10 | 741X443 | A>G | 0/1 | c.949A>G | p.Ile317Val | ENSBTAT00000027885.5 | Tolerated (1) |
| PLEKHG3 | 10 | 77194351 | G>A | 0/1 | c.2356G>A | p.Ala786Thr | ENSBTAT00000039904.4 | Tolerated (0.96) |
| RDH11 | 10 | 80135484 | C>T | 0/1 | c.901G>A | p.ValX1Ile | ENSBTAT00000002535.4 | Tolerated (0.32) |
| RDH11 | 10 | 80135522 | T>C | 0/1 | c.863A>G | p.His288Arg | ENSBTAT00000002535.4 | Tolerated (0.52) |
| RDH11 | 10 | 80135523 | G>T | 0/1 | c.862C>A | p.His288Asn | ENSBTAT00000002535.4 | Tolerated (0.23) |
| SIPA1L1 | 10 | 83589225 | G>T | 0/1 | c.3943G>T | p.Ala1315Ser | ENSBTAT00000035650.4 | Tolerated (0.54) |
| SIPA1L1 | 10 | 83589247 | T>A | 0/1 | c.3965T>A | p.Phe1322Tyr | ENSBTAT00000035650.4 | Tolerated (1) |
| RPS6KL1 | 10 | 86565053 | A>G | 0/1 | c.464T>C | p.Val155Ala | ENSBTAT00000063286.1 | Deleterious (0.01) |
| MLH3 | 10 | 86671094 | C>T | 0/1 | c.4271G>A | p.Arg1424Lys | ENSBTAT00000061421.2 | Tolerated (1) |
| GPATCH2L | 10 | 88469923 | TG>T | 0/1 | c.163delG | p.Ala55fs | ENSBTAT00000004016.5 | - |
| ENSBTAG00000040167 | 10 | 92890454 | C>T | 0/1 | c.299G>A | p.Arg100His | ENSBTAT00000054915.2 | Tolerated (0.16) |
| SPG11 | 10 | 104019931 | G>C | 0/1 | c.5585C>G | p.Thr1862Ser | ENSBTAT00000000470.5 | Tolerated (0.35) |
| SPG11 | 10 | 104019969 | G>C | 0/1 | c.5547C>G | p.Asn1849Lys | ENSBTAT00000000470.5 | Tolerated (1) |
| SPG11 | 10 | 104019970 | T>A | 0/1 | c.5546A>T | p.Asn1849Ile | ENSBTAT00000000470.5 | Deleterious (0) |
| SPG11 | 10 | 104019977 | A>G | 0/1 | c.5539T>C | p.Phe1847Leu | ENSBTAT00000000470.5 | Deleterious (0.04) |
| **Gene** | **BTA** | **Position** | **Base change** | **Genotype** | **cDNA** | **Protein** | **Transcript** | **SIFT** |
| SPG11 | 10 | 104019986 | C>T | 0/1 | c.55XG>A | p.Glu1844Lys | ENSBTAT00000000470.5 | Deleterious (0.03) |
| SPG11 | 10 | 104019995 | A>T | 0/1 | c.5521T>A | p.Leu1841Ile | ENSBTAT00000000470.5 | Deleterious (0.01) |
| CNNM3 | 11 | 2732197 | C>G | 0/1 | c.1092C>G | p.Asp364Glu | ENSBTAT00000014394.5 | Tolerated (1) |
| RTKN | 11 | 10184294 | A>C | 0/1 | c.1170A>C | p.Glu390Asp | ENSBTAT00000000619.3 | Tolerated (0.61) |
| RTKN | 11 | 10184316 | G>A | 0/1 | c.1192G>A | p.Ala398Thr | ENSBTAT00000000619.3 | Tolerated (0.96) |
| BIRC6 | 11 | 15111888 | A>T | 0/1 | c.10411A>T | p.Met3471Leu | ENSBTAT00000061440.2 | - |
| BIRC6 | 11 | 15111892 | A>G | 0/1 | c.10415A>G | p.Lys3472Arg | ENSBTAT00000061440.2 | - |
| BIRC6 | 11 | 15111933 | T>A | 0/1 | c.10456T>A | p.Ser3486Thr | ENSBTAT00000061440.2 | - |
| BIRC6 | 11 | 15111936 | A>G | 0/1 | c.10459A>G | p.Ile3487Val | ENSBTAT00000061440.2 | - |
| BIRC6 | 11 | 15111974 | G>T | 0/1 | c.10497G>T | p.Leu3499Phe | ENSBTAT00000061440.2 | - |
| LRPPRC | 11 | 26237375 | CA>C | 0/1 | c.2432delT | p.Leu811fs | ENSBTAT00000021773.5 | - |
| EPCAM | 11 | 29636350 | TA>T | 0/1 | c.945delA | p.Ter315fs | ENSBTAT00000008487.5 | - |
| B3GNT2 | 11 | 60685801 | C>A | 0/1 | c.763C>A | p.Leu255Ile | ENSBTAT00000024024.4 | Tolerated (1) |
| B3GNT2 | 11 | 60685829 | G>A | 0/1 | c.791G>A | p.Gly264Glu | ENSBTAT00000024024.4 | Tolerated (1) |
| EHBP1 | 11 | 61261672 | TG>T | 0/1 | c.343delG | p.Ala115fs | ENSBTAT00000061137.2 | - |
| PCYOX1 | 11 | 68561910 | A>G | 0/1 | c.212A>G | p.Lys71Arg | ENSBTAT00000044517.3 | Tolerated (0.61) |
| PCYOX1 | 11 | 68561915 | A>G | 0/1 | c.217A>G | p.Lys73Glu | ENSBTAT00000044517.3 | Tolerated (1) |
| PCYOX1 | 11 | 68561939 | T>G | 0/1 | c.241T>G | p.Leu81Val | ENSBTAT00000044517.3 | Tolerated (1) |
| PCYOX1 | 11 | 68561943 | A>C | 0/1 | c.245A>C | p.Asn82Thr | ENSBTAT00000044517.3 | Tolerated (1) |
| **Gene** | **BTA** | **Position** | **Base change** | **Genotype** | **cDNA** | **Protein** | **Transcript** | **SIFT** |
| PCYOX1 | 11 | 68561949 | A>G | 0/1 | c.251A>G | p.Gln84Arg | ENSBTAT00000044517.3 | Tolerated (0.25) |
| CLIP4 | 11 | 70705936 | G>A | 0/1 | c.515C>T | p.Thr172Ile | ENSBTAT00000009702.4 | Tolerated (0.07) |
| DNMT3A | 11 | 74046880 | T>C | 0/1 | c.794T>C | p.Ile265Thr | ENSBTAT00000032936.4 | Tolerated (0.07) |
| DNMT3A | 11 | 74055247 | G>A | 0/1 | c.2257G>A | p.Val753Met | ENSBTAT00000032936.4 | Deleterious (0.02) |
| DNMT3A | 11 | 74055256 | G>A | 0/1 | c.2266G>A | p.Asp756Asn | ENSBTAT00000032936.4 | Deleterious (0.02) |
| KIDINS220 | 11 | 885076X | C>G | 0/1 | c.2018C>G | p.Ala673Gly | ENSBTAT00000061437.2 | Tolerated (1) |
| KIDINS220 | 11 | 88507671 | A>C | 0/1 | c.2059A>C | p.Met687Leu | ENSBTAT00000061437.2 | Tolerated (1) |
| KIDINS220 | 11 | 88558759 | G>A | 0/1 | c.4531G>A | p.Gly1511Ser | ENSBTAT00000061437.2 | Tolerated low confidence(0.77) |
| CMPK2 | 11 | 90046077 | T>G | 0/1 | c.850T>G | p.Ser284Ala | ENSBTAT00000026613.5 | Tolerated (0.73) |
| GPR21 | 11 | 94189817 | C>T | 0/1 | c.854C>T | p.Ala285Val | ENSBTAT00000053314.1 | Tolerated (0.38) |
| OLFML2A | 11 | 95805462 | A>G | 0/1 | c.1900A>G | p.Lys634Glu | ENSBTAT00000006372.2 | Deleterious (0) |
| OLFML2A | 11 | 95805474 | C>T | 0/1 | c.1912C>T | p.Leu638Phe | ENSBTAT00000006372.2 | Deleterious (0) |
| NUP188 | 11 | 99486440 | GT>G | 0/1 | c.1320delT | p.His442fs | ENSBTAT000000140X.5 | - |
| NUP188 | 11 | 99486444 | C>A | 0/1 | c.1321C>A | p.Pro441Thr | ENSBTAT000000140X.5 | Deleterious (0) |
| EXOSC2 | 11 | 1009977X | G>T | 0/1 | c.63G>T | p.Glu21Asp | ENSBTAT00000063775.1 | Tolerated (0.75) |
| EXOSC2 | 11 | 100997732 | G>C | 0/1 | c.65G>C | p.Ser22Thr | ENSBTAT00000063775.1 | Tolerated (0.64) |
| SPACA9 | 11 | 102959378 | A>T | 0/1 | c.X4A>T | p.Thr102Ser | ENSBTAT00000006841.2 | Tolerated (0.72) |
| OLFM4 | 12 | 10667671 | G>A | 0/1 | c.1244C>T | p.Ala415Val | ENSBTAT000000X920.3 | Tolerated (0.15) |
| RB1 | 12 | 18298482 | T>C | 0/1 | c.1748T>C | p.Val583Ala | ENSBTAT00000008728.4 | Tolerated (1) |
| **Gene** | **BTA** | **Position** | **Base change** | **Genotype** | **cDNA** | **Protein** | **Transcript** | **SIFT** |
| RB1 | 12 | 18298490 | T>C | 0/1 | c.1756T>C | p.Phe586Leu | ENSBTAT00000008728.4 | Tolerated (0.79) |
| RB1 | 12 | 18298496 | C>T | 0/1 | c.1762C>T | p.Pro588Ser | ENSBTAT00000008728.4 | Tolerated (0.52) |
| RCBTB2 | 12 | 18340109 | A>G | 0/1 | c.392T>C | p.Ile131Thr | ENSBTAT00000008733.5 | Tolerated (0.26) |
| RCBTB2 | 12 | 18340145 | T>C | 0/1 | c.356A>G | p.Tyr119Cys | ENSBTAT00000008733.5 | Tolerated (0.54) |
| RCBTB2 | 12 | 18340160 | G>T | 0/1 | c.341C>A | p.Ala114Asp | ENSBTAT00000008733.5 | Tolerated (0.24) |
| CKAP2 | 12 | 21600823 | A>C | 0/1 | c.1795T>G | p.Cys599Gly | ENSBTAT00000052675.1 | Tolerated (0.61) |
| CKAP2 | 12 | 21600858 | C>G | 0/1 | c.1760G>C | p.Cys587Ser | ENSBTAT00000052675.1 | Tolerated (0.14) |
| CKAP2 | 12 | 21600859 | A>G | 0/1 | c.1759T>C | p.Cys587Arg | ENSBTAT00000052675.1 | Deleterious (0) |
| CKAP2 | 12 | 21600892 | T>A | 0/1 | c.1726A>T | p.Lys576* | ENSBTAT00000052675.1 | - |
| CKAP2 | 12 | 21600897 | G>A | 0/1 | c.1721C>T | p.Thr574Ile | ENSBTAT00000052675.1 | Tolerated (0.11) |
| CKAP2 | 12 | 21600910 | G>T | 0/1 | c.1708C>A | p.Leu570Ile | ENSBTAT00000052675.1 | Tolerated (0.37) |
| CKAP2 | 12 | 21600912 | C>G | 0/1 | c.1706G>C | p.Arg569Pro | ENSBTAT00000052675.1 | Deleterious (0) |
| TRPC4 | 12 | 24085235 | C>A | 0/1 | c.820C>A | p.Arg274Ser | ENSBTAT00000012378.3 | Tolerated (0.45) |
| TRPC4 | 12 | 24085245 | A>G | 0/1 | c.8XA>G | p.Asn277Ser | ENSBTAT00000012378.3 | Tolerated (0.78) |
| TRPC4 | 12 | 24085253 | C>A | 0/1 | c.838C>A | p.Leu280Met | ENSBTAT00000012378.3 | Tolerated (0.08) |
| PARP4 | 12 | 36710640 | C>T | 0/1 | c.1341G>A | p.Met447Ile | ENSBTAT00000035651.4 | Deleterious (0.04) |
| PARP4 | 12 | 36710674 | T>G | 0/1 | c.1X7A>C | p.His436Pro | ENSBTAT00000035651.4 | Tolerated (1) |
| PARP4 | 12 | 36710677 | C>T | 0/1 | c.1X4G>A | p.Arg435Lys | ENSBTAT00000035651.4 | Tolerated (1) |
| PARP4 | 12 | 36710691 | G>C | 0/1 | c.1290C>G | p.His4XGln | ENSBTAT00000035651.4 | Tolerated (0.65) |
| **Gene** | **BTA** | **Position** | **Base change** | **Genotype** | **cDNA** | **Protein** | **Transcript** | **SIFT** |
| PARP4 | 12 | 36710696 | C>T | 0/1 | c.1285G>A | p.Gly429Ser | ENSBTAT00000035651.4 | Tolerated (1) |
| PARP4 | 12 | 36710698 | T>A | 0/1 | c.1283A>T | p.Gln428Leu | ENSBTAT00000035651.4 | Tolerated (0.78) |
| PARP4 | 12 | 36710699 | G>A | 0/1 | c.1282C>T | p.Gln428* | ENSBTAT00000035651.4 | - |
| PARP4 | 12 | 36710711 | T>C | 0/1 | c.1270A>G | p.Thr424Ala | ENSBTAT00000035651.4 | Tolerated (0.62) |
| PARP4 | 12 | 36710716 | C>T | 0/1 | c.1265G>A | p.Ser422Asn | ENSBTAT00000035651.4 | Tolerated (1) |
| PARP4 | 12 | 36710722 | C>T | 0/1 | c.1259G>A | p.Arg420Lys | ENSBTAT00000035651.4 | Tolerated (0.2) |
| TBC1D4 | 12 | 50708644 | C>T | 0/1 | c.2414G>A | p.Ser805Asn | ENSBTAT00000066234.1 | Tolerated (0.32) |
| MYCBP2 | 12 | 52505263 | T>C | 0/1 | c.13133A>G | p.Asn4378Ser | ENSBTAT00000025100.5 | - |
| USP6NL | 13 | 12831268 | T>A | 0/1 | c.1145T>A | p.Phe382Tyr | ENSBTAT00000025957.5 | Tolerated (0.43) |
| ITIH5 | 13 | 16403729 | G>A | 0/1 | c.2675G>A | p.Arg892Lys | ENSBTAT00000048699.3 | Tolerated (1) |
| ITGB1 | 13 | 20274025 | A>G | 0/1 | c.626A>G | p.Asn209Ser | ENSBTAT00000056716.1 | Tolerated (0.39) |
| ARMC3 | 13 | 24270836 | C>T | 0/1 | c.557C>T | p.Thr186Ile | ENSBTAT00000061467.2 | Deleterious (0.04) |
| ARMC3 | 13 | 24270859 | CCT>C | 0/1 | c.582 583delTC | p.Pro195fs | ENSBTAT00000061467.2 | Deleterious (0.01) |
| ARMC3 | 13 | 24270864 | T>TCA | 0/1 | c.585 586insCA | p.Ile196fs | ENSBTAT00000061467.2 | - |
| GPR158 | 13 | 26526387 | A>G | 0/1 | c.3488A>G | p.Gln1163Arg | ENSBTAT00000032890.3 | Tolerated (0.22) |
| GPR158 | 13 | 26526390 | C>A | 0/1 | c.3491C>A | p.Pro1164Gln | ENSBTAT00000032890.3 | Tolerated (0.57) |
| GPR158 | 13 | 26526399 | T>C | 0/1 | c.3500T>C | p.Met1167Thr | ENSBTAT00000032890.3 | Tolerated (1) |
| GPR158 | 13 | 26526400 | G>A | 0/1 | c.3501G>A | p.Met1167Ile | ENSBTAT00000032890.3 | Tolerated (0.52) |
| TMEM236 | 13 | 32492755 | G>A | 0/1 | c.464G>A | p.Arg155Lys | ENSBTAT00000025264.4 | Tolerated (0.61) |
| **Gene** | **BTA** | **Position** | **Base change** | **Genotype** | **cDNA** | **Protein** | **Transcript** | **SIFT** |
| ENSBTAG00000027444 | 13 | 34947072 | T>G | 0/1 | c.984A>C | p.Leu328Phe | ENSBTAT00000052009.2 | - |
| ENSBTAG00000027444 | 13 | 34947077 | A>G | 0/1 | c.979T>C | p.Ser327Pro | ENSBTAT00000052009.2 | - |
| ENSBTAG00000027444 | 13 | 34947086 | T>C | 0/1 | c.970A>G | p.Thr324Ala | ENSBTAT00000052009.2 | Tolerated (0.5) |
| ENSBTAG00000027444 | 13 | 34947089 | G>C | 0/1 | c.967C>G | p.Leu323Val | ENSBTAT00000052009.2 | Tolerated (0.66) |
| SNRPB | 13 | 53137393 | A>G | 0/1 | c.23A>G | p.Lys8Arg | ENSBTAT00000001379.4 | Tolerated (0.11) |
| YTHDF1 | 13 | 54812777 | GC>G | 1/1 | c.979delC | p.Gln327fs | ENSBTAT00000013703.5 | - |
| SYCP2 | 13 | 57180881 | TAG>T | 0/1 | c.2677 2678delGA | p.Glu893fs | ENSBTAT00000004704.5 | - |
| DNMT3B | 13 | 62703841 | G>C | 0/1 | c.365G>C | p.Ser122Thr | ENSBTAT00000005094.5 | Tolerated low confidence(0.74) |
| DNMT3B | 13 | 62703846 | A>G | 0/1 | c.370A>G | p.Thr124Ala | ENSBTAT00000005094.5 | Tolerated low confidence(0.79) |
| DNMT3B | 13 | 62703853 | C>G | 0/1 | c.377C>G | p.Thr126Ser | ENSBTAT00000005094.5 | Tolerated low confidence(1) |
| MYH7B | 13 | 64911210 | G>A | 0/1 | c.5227G>A | p.Ala1743Thr | ENSBTAT00000039242.4 | Tolerated (0.33) |
| MROH8 | 13 | 6675X77 | A>T | 0/1 | c.2170T>A | p.Ser724Thr | ENSBTAT00000018251.5 | Tolerated (0.91) |
| MROH8 | 13 | 66753115 | T>C | 0/1 | c.2132A>G | p.Lys711Arg | ENSBTAT00000018251.5 | Tolerated (0.1) |
| RBPJL | 13 | 74377868 | A>C | 0/1 | c.707A>C | p.Asp236Ala | ENSBTAT00000014992.5 | Tolerated (1) |
| ENSBTAG00000009675 | 13 | 75936270 | T>A | 0/1 | c.18T>A | p.Phe6Leu | ENSBTAT00000065334.1 | Deleterious (0.01) |
| ENSBTAG00000009675 | 13 | 75936286 | G>A | 0/1 | c.34G>A | p.Val12Ile | ENSBTAT00000065334.1 | Deleterious (0.01) |
| ENSBTAG00000009675 | 13 | 75936310 | C>T | 0/1 | c.58C>T | p.His20Tyr | ENSBTAT00000065334.1 | Deleterious (0.05) |
| ENSBTAG00000009675 | 13 | 75936311 | A>G | 0/1 | c.59A>G | p.His20Arg | ENSBTAT00000065334.1 | Tolerated (0.21) |
| **Gene** | **BTA** | **Position** | **Base change** | **Genotype** | **cDNA** | **Protein** | **Transcript** | **SIFT** |
| ENSBTAG00000009675 | 13 | 75936317 | G>A | 0/1 | c.65G>A | p.Ser22Asn | ENSBTAT00000065334.1 | Tolerated (0.21) |
| ENSBTAG00000009675 | 13 | 75936325 | A>G | 0/1 | c.73A>G | p.Lys25Glu | ENSBTAT00000065334.1 | Tolerated (0.08) |
| ENSBTAG00000009675 | 13 | 75936331 | G>C | 0/1 | c.79G>C | p.Glu27Gln | ENSBTAT00000065334.1 | Deleterious (0.04) |
| ENSBTAG00000009675 | 13 | 75936341 | G>A | 0/1 | c.89G>A | p.ArgXGln | ENSBTAT00000065334.1 | Deleterious (0.02) |
| ENSBTAG00000009675 | 13 | 75936359 | C>A | 0/1 | c.107C>A | p.Thr36Asn | ENSBTAT00000065334.1 | Tolerated (1) |
| STAU1 | 13 | 77965086 | A>G | 0/1 | c.1619T>C | p.Val540Ala | ENSBTAT00000000X1.5 | Tolerated (0.94) |
| SLA | 14 | 9369354 | GT>G | 0/1 | c.777delT | p.Ser259fs | ENSBTAT00000010298.4 | - |
| RNF139 | 14 | 17173460 | A>T | 0/1 | c.1000T>A | p.Phe334Ile | ENSBTAT00000027187.5 | Deleterious (0) |
| FAM91A1 | 14 | 17687544 | CT>C | 0/1 | c.1077delA | p.Asp360fs | ENSBTAT00000006615.4 | - |
| CYP7A1 | 14 | 26356227 | A>C | 0/1 | c.282T>G | p.Phe94Leu | ENSBTAT00000006957.2 | Tolerated (1) |
| CYP7A1 | 14 | 26356254 | A>C | 0/1 | c.255T>G | p.His85Gln | ENSBTAT00000006957.2 | Tolerated (0.46) |
| DNAJC5B | 14 | 32143251 | A>G | 0/1 | c.572A>G | p.Glu191Gly | ENSBTAT00000020262.3 | Tolerated (1) |
| SBSPON | 14 | 38561404 | TA>T | 0/1 | c.420delT | p.Phe140fs | ENSBTAT00000022924.5 | - |
| CRISPLD1 | 14 | 40422527 | G>C | 0/1 | c.250G>C | p.Glu84Gln | ENSBTAT00000005784.5 | Deleterious (0.02) |
| TMEM74 | 14 | 57784693 | A>G | 0/1 | c.535A>G | p.Ile179Val | ENSBTAT00000052642.2 | Tolerated (0.16) |
| TMEM74 | 14 | 57784747 | A>G | 0/1 | c.589A>G | p.Ile197Val | ENSBTAT00000052642.2 | Tolerated (1) |
| DCAF13 | 14 | 63253485 | T>C | 0/1 | c.124A>G | p.Ile42Val | ENSBTAT00000028594.5 | Tolerated (0.84) |
| MATN2 | 14 | 68595888 | T>A | 0/1 | c.685A>T | p.Thr229Ser | ENSBTAT00000022815.5 | Tolerated (0.67) |
| MATN2 | 14 | 68595892 | A>C | 0/1 | c.681T>G | p.Ile227Met | ENSBTAT00000022815.5 | Tolerated (0.17) |
| **Gene** | **BTA** | **Position** | **Base change** | **Genotype** | **cDNA** | **Protein** | **Transcript** | **SIFT** |
| RBM12B | 14 | 72875741 | G>C | 0/1 | c.877G>C | p.Val293Leu | ENSBTAT00000017837.4 | Tolerated (1) |
| DECR1 | 14 | 76023536 | G>C | 0/1 | c.943C>G | p.Gln315Glu | ENSBTAT00000026202.4 | Tolerated (0.33) |
| DECR1 | 14 | 76023537 | A>C | 0/1 | c.942T>G | p.Asp314Glu | ENSBTAT00000026202.4 | Tolerated (1) |
| JRKL | 15 | 14118784 | G>A | 0/1 | c.763C>T | p.His255Tyr | ENSBTAT00000056224.2 | Tolerated (0.44) |
| MAML2 | 15 | 14555943 | T>C | 0/1 | c.2428T>C | p.Ser810Pro | ENSBTAT00000054262.2 | Tolerated (1) |
| DIXDC1 | 15 | 22637482 | A>G | 0/1 | c.1047A>G | p.Ile349Met | ENSBTAT00000040095.3 | Tolerated (1) |
| UBE4A | 15 | 29507205 | CA>C | 0/1 | c.2552delA | p.Asn851fs | ENSBTAT00000026289.4 | - |
| KMT2A | 15 | 29672451 | G>A | 0/1 | c.1921G>A | p.Gly641Ser | ENSBTAT00000024084.5 | - |
| NLRX1 | 15 | X282276 | TC>T | 0/1 | c.667delC | p.Leu223fs | ENSBTAT00000016601.5 | - |
| TECTA | 15 | 32279355 | G>T | 0/1 | c.1153G>T | p.Val385Leu | ENSBTAT00000047085.2 | Deleterious (0.01) |
| BTBD10 | 15 | 39800540 | A>G | 0/1 | c.319A>G | p.Ile107Val | ENSBTAT00000039968.2 | Tolerated low confidence(0.42) |
| PPFIBP2 | 15 | 45695295 | T>C | 0/1 | c.1264A>G | p.Thr422Ala | ENSBTAT00000021483.5 | Tolerated (0.19) |
| ENSBTAG00000038452 | 15 | 47810298 | A>C | 0/1 | c.649A>C | p.Ile217Leu | ENSBTAT00000056783.2 | Tolerated (0.81) |
| ENSBTAG00000038452 | 15 | 47810343 | C>T | 0/1 | c.694C>T | p.Gln232* | ENSBTAT00000056783.2 | - |
| ENSBTAG00000038452 | 15 | 47810344 | A>G | 0/1 | c.695A>G | p.Gln232Arg | ENSBTAT00000056783.2 | Tolerated (0.92) |
| HBB | 15 | 49023371 | A>G | 0/1 | c.214A>G | p.Asn72Asp | ENSBTAT00000045694.3 | Tolerated (0.68) |
| HBB | 15 | 49023377 | A>C | 0/1 | c.220A>C | p.Met74Leu | ENSBTAT00000045694.3 | Tolerated (0.53) |
| HBB | 15 | 49023383 | C>A | 0/1 | c.226C>A | p.His76Asn | ENSBTAT00000045694.3 | Tolerated (1) |
| HBB | 15 | 49023392 | G>A | 0/1 | c.235G>A | p.Asp79Asn | ENSBTAT00000045694.3 | Tolerated (1) |
| **Gene** | **BTA** | **Position** | **Base change** | **Genotype** | **cDNA** | **Protein** | **Transcript** | **SIFT** |
| HBB | 15 | 49023413 | G>A | 0/1 | c.256G>A | p.Ala86Thr | ENSBTAT00000045694.3 | Tolerated (0.16) |
| HBB | 15 | 49023414 | C>A | 0/1 | c.257C>A | p.Ala86Glu | ENSBTAT00000045694.3 | Tolerated (0.42) |
| ENSBTAG00000039298 | 15 | 5013X58 | G>T | 0/1 | c.868C>A | p.Leu290Ile | ENSBTAT0000005X48.2 | Tolerated (0.05) |
| ENSBTAG00000039298 | 15 | 5013X91 | C>T | 0/1 | c.835G>A | p.Val279Met | ENSBTAT0000005X48.2 | Tolerated (0.07) |
| ATG16L2 | 15 | 53147715 | T>C | 0/1 | c.842T>C | p.Met281Thr | ENSBTAT00000025375.5 | Tolerated (0.22) |
| C2CD3 | 15 | 54320367 | G>T | 0/1 | c.1531C>A | p.Leu511Ile | ENSBTAT00000061233.2 | Tolerated (0.45) |
| PGM2L1 | 15 | 54491258 | C>A | 0/1 | c.1564G>T | p.Ala522Ser | ENSBTAT00000001019.5 | Tolerated (0.45) |
| ANO3 | 15 | 58011380 | CT>C | 0/1 | c.685delT | p.Tyr229fs | ENSBTAT00000029137.5 | Tolerated (0.31) |
| MADD | 15 | 78367234 | T>C | 0/1 | c.3818T>C | p.Phe1273Ser | ENSBTAT00000028927.5 | - |
| KBTBD4 | 15 | 78578674 | T>A | 0/1 | c.868A>T | p.Thr290Ser | ENSBTAT00000024595.5 | Tolerated (0.65) |
| OR4X1 | 15 | 79187484 | C>T | 0/1 | c.200C>T | p.Ala67Val | ENSBTAT00000039564.2 | Tolerated (0.06) |
| OR4X1 | 15 | 79187539 | G>T | 0/1 | c.255G>T | p.Met85Ile | ENSBTAT00000039564.2 | Tolerated (1) |
| OR4X1 | 15 | 79187546 | A>C | 0/1 | c.262A>C | p.Lys88Gln | ENSBTAT00000039564.2 | Tolerated (0.48) |
| OR4X1 | 15 | 79187549 | G>A | 0/1 | c.265G>A | p.Val89Ile | ENSBTAT00000039564.2 | Deleterious (0.02) |
| OR4X1 | 15 | 79187550 | T>C | 0/1 | c.266T>C | p.Val89Ala | ENSBTAT00000039564.2 | Tolerated (0.08) |
| ENSBTAG00000040341 | 15 | 79726339 | C>T | 0/1 | c.404G>A | p.Ser135Asn | ENSBTAT00000056973.2 | Tolerated (0.14) |
| ENSBTAG00000040341 | 15 | 79726346 | C>T | 0/1 | c.397G>A | p.Ala133Thr | ENSBTAT00000056973.2 | Tolerated (0.72) |
| ENSBTAG00000040341 | 15 | 79726360 | C>T | 0/1 | c.383G>A | p.Ser128Asn | ENSBTAT00000056973.2 | Tolerated (1) |
| ATP2B4 | 16 | 1281221 | G>A | 0/1 | c.776G>A | p.Ser259Asn | ENSBTAT00000018688.5 | Tolerated (1) |
| **Gene** | **BTA** | **Position** | **Base change** | **Genotype** | **cDNA** | **Protein** | **Transcript** | **SIFT** |
| ZBED6 | 16 | 1402395 | G>A | 0/1 | c.1939G>A | p.Ala647Thr | ENSBTAT00000063570.1 | Tolerated (0.62) |
| IL24 | 16 | 4608644 | G>A | 0/1 | c.559C>T | p.Leu187Phe | ENSBTAT00000010325.5 | Tolerated (0.77) |
| IL24 | 16 | 4608647 | G>C | 0/1 | c.556C>G | p.Gln186Glu | ENSBTAT00000010325.5 | Deleterious low confidence(0.04) |
| IL24 | 16 | 4608667 | C>G | 0/1 | c.536G>C | p.Ser179Thr | ENSBTAT00000010325.5 | Deleterious (0.01) |
| IL24 | 16 | 4608681 | C>G | 0/1 | c.522G>C | p.Met174Ile | ENSBTAT00000010325.5 | Tolerated (1) |
| IL24 | 16 | 4608683 | T>C | 0/1 | c.520A>G | p.Met174Val | ENSBTAT00000010325.5 | Tolerated (0.08) |
| USH2A | 16 | 20137947 | T>C | 0/1 | c.4739A>G | p.Tyr1580Cys | ENSBTAT00000061112.2 | - |
| USH2A | 16 | 20137972 | T>G | 0/1 | c.4714A>C | p.Ile1572Leu | ENSBTAT00000061112.2 | - |
| DUSP10 | 16 | 25934261 | C>G | 0/1 | c.2XG>C | p.Ser77Thr | ENSBTAT00000002265.3 | Deleterious low confidence(0.03) |
| DUSP10 | 16 | 25934283 | A>C | 0/1 | c.208T>G | p.Ser70Ala | ENSBTAT00000002265.3 | Tolerated (0.14) |
| SMYD3 | 16 | 31589426 | A>T | 0/1 | c.31A>T | p.Thr11Ser | ENSBTAT00000047138.2 | Tolerated (1) |
| SMYD3 | 16 | 31589429 | A>G | 0/1 | c.34A>G | p.Thr12Ala | ENSBTAT00000047138.2 | Tolerated (0.48) |
| SMYD3 | 16 | 31589432 | G>A | 0/1 | c.37G>A | p.Asp13Asn | ENSBTAT00000047138.2 | Tolerated (0.16) |
| SMYD3 | 16 | 31589433 | A>G | 0/1 | c.38A>G | p.Asp13Gly | ENSBTAT00000047138.2 | Tolerated (1) |
| SMYD3 | 16 | 31589436 | A>G | 0/1 | c.41A>G | p.Lys14Arg | ENSBTAT00000047138.2 | Tolerated (0.38) |
| SMYD3 | 16 | 31589457 | T>C | 0/1 | c.62T>C | p.Leu21Ser | ENSBTAT00000047138.2 | Tolerated (0.86) |
| SMYD3 | 16 | 31589463 | C>A | 0/1 | c.68C>A | p.Pro23Gln | ENSBTAT00000047138.2 | Tolerated (0.58) |
| SMYD3 | 16 | 31589472 | C>CCG | 0/1 | c.77 78insCG | p.Leu29fs | ENSBTAT00000047138.2 | - |
| **Gene** | **BTA** | **Position** | **Base change** | **Genotype** | **cDNA** | **Protein** | **Transcript** | **SIFT** |
| SMYD3 | 16 | 31589477 | GAA>G | 0/1 | c.83 84delAA | p.Glu28fs | ENSBTAT00000047138.2 | - |
| HNRNPU | 16 | 33168984 | TTG>T | 0/1 | c.1119 1120delTG | p.Cys373fs | ENSBTAT00000052059.2 | - |
| METTL13 | 16 | 40051598 | G>C | 0/1 | c.980G>C | p.Ser327Thr | ENSBTAT00000023571.4 | Tolerated (0.06) |
| KIF1B | 16 | 44122735 | TAA>T | 0/1 | c.2895 2896delTT | p.Phe965fs | ENSBTAT00000006538.5 | - |
| ERRFI1 | 16 | 46213334 | AC>A | 0/1 | c.710delC | p.Pro237fs | ENSBTAT00000008675.4 | - |
| PARK7 | 16 | 46263205 | C>T | 0/1 | c.151G>A | p.Val51Ile | ENSBTAT00000027339.3 | Tolerated (0.3) |
| ESPN | 16 | 47771052 | C>G | 0/1 | c.1097G>C | p.Arg366Pro | ENSBTAT00000045529.3 | Tolerated (1) |
| ESPN | 16 | 47771114 | G>C | 0/1 | c.1035C>G | p.Asp345Glu | ENSBTAT00000045529.3 | Tolerated (1) |
| ESPN | 16 | 47771119 | T>C | 0/1 | c.10XA>G | p.Thr344Ala | ENSBTAT00000045529.3 | Tolerated (1) |
| SLC9C2 | 16 | 56489540 | A>T | 0/1 | c.298T>A | p.Leu100Ile | ENSBTAT00000048236.3 | Tolerated (0.76) |
| RFWD2 | 16 | 58685139 | A>T | 0/1 | c.1356T>A | p.Tyr452* | ENSBTAT00000044452.3 | - |
| CEP350 | 16 | 62666910 | C>T | 0/1 | c.1622C>T | p.Ala541Val | ENSBTAT00000010631.5 | Tolerated (0.16) |
| CEP350 | 16 | 62666915 | A>G | 0/1 | c.1627A>G | p.Thr543Ala | ENSBTAT00000010631.5 | Tolerated (0.63) |
| GLUL | 16 | 64956003 | A>C | 0/1 | c.72T>G | p.Asp24Glu | ENSBTAT00000038488.4 | Tolerated (0.6) |
| GLUL | 16 | 64956020 | C>A | 0/1 | c.55G>T | p.Ala19Ser | ENSBTAT00000038488.4 | Tolerated (1) |
| DHX9 | 16 | 65395117 | C>G | 0/1 | c.763C>G | p.Pro255Ala | ENSBTAT00000026409.4 | Tolerated (1) |
| LAMC1 | 16 | 65662401 | A>T | 0/1 | c.4618A>T | p.Thr1540Ser | ENSBTAT00000015881.5 | Tolerated (0.9) |
| RGL1 | 16 | 66397441 | A>G | 0/1 | c.787A>G | p.Ile263Val | ENSBTAT00000026153.5 | Tolerated (0.81) |
| HMCN1 | 16 | 68407352 | A>G | 0/1 | c.920A>G | p.LysX7Arg | ENSBTAT00000061144.2 | - |
| **Gene** | **BTA** | **Position** | **Base change** | **Genotype** | **cDNA** | **Protein** | **Transcript** | **SIFT** |
| CENPF | 16 | 70454145 | T>G | 0/1 | c.8185A>C | p.Ile2729Leu | ENSBTAT00000033913.4 | - |
| CENPF | 16 | 70454148 | C>G | 0/1 | c.8182G>C | p.Glu2728Gln | ENSBTAT00000033913.4 | - |
| CENPF | 16 | 70465609 | G>A | 0/1 | c.4358C>T | p.Ser1453Phe | ENSBTAT00000033913.4 | - |
| CENPF | 16 | 70465612 | C>T | 0/1 | c.4355G>A | p.Ser1452Asn | ENSBTAT00000033913.4 | - |
| CENPF | 16 | 70465623 | C>T | 0/1 | c.4344G>A | p.Met1448Ile | ENSBTAT00000033913.4 | - |
| CENPF | 16 | 70465624 | A>G | 0/1 | c.4343T>C | p.Met1448Thr | ENSBTAT00000033913.4 | - |
| CENPF | 16 | 70465634 | C>T | 0/1 | c.4333G>A | p.Val1445Ile | ENSBTAT00000033913.4 | - |
| ANGEL2 | 16 | 72476148 | T>A | 0/1 | c.316T>A | p.Phe106Ile | ENSBTAT00000004825.5 | Tolerated low confidence(1) |
| CAMSAP2 | 16 | 81318913 | T>G | 0/1 | c.2252T>G | p.Leu751Arg | ENSBTAT00000010723.5 | Tolerated (0.37) |
| MSMO1 | 17 | 493522 | A>G | 0/1 | c.145A>G | p.Ile49Val | ENSBTAT00000003991.2 | Tolerated (0.28) |
| MSMO1 | 17 | 493540 | C>T | 0/1 | c.163C>T | p.Leu55Phe | ENSBTAT00000003991.2 | Tolerated (0.6) |
| MSMO1 | 17 | 493547 | T>C | 0/1 | c.170T>C | p.Val57Ala | ENSBTAT00000003991.2 | Tolerated (0.2) |
| MSMO1 | 17 | 501919 | A>C | 0/1 | c.577A>C | p.Ile193Leu | ENSBTAT00000003991.2 | Tolerated (1) |
| MSMO1 | 17 | 501921 | A>G | 0/1 | c.579A>G | p.Ile193Met | ENSBTAT00000003991.2 | Tolerated (0.15) |
| GATB | 17 | 5983831 | G>C | 0/1 | c.324G>C | p.Leu108Phe | ENSBTAT0000005X94.1 | Deleterious (0) |
| SCLT1 | 17 | 29212962 | A>G | 0/1 | c.197A>G | p.Lys66Arg | ENSBTAT00000061229.2 | Tolerated (1) |
| SCLT1 | 17 | 29212971 | A>G | 0/1 | c.206A>G | p.Lys69Arg | ENSBTAT00000061229.2 | Tolerated (0.41) |
| HSPA4L | 17 | X285478 | A>G | 0/1 | c.83T>C | p.Leu28Ser | ENSBTAT00000020838.5 | Tolerated low confidence(0.74) |
| **Gene** | **BTA** | **Position** | **Base change** | **Genotype** | **cDNA** | **Protein** | **Transcript** | **SIFT** |
| C4orf45 | 17 | 40987534 | C>G | 0/1 | c.186C>G | p.Asn62Lys | ENSBTAT00000047680.1 | Tolerated (0.33) |
| ZNF140 | 17 | 45116197 | A>G | 0/1 | c.1265A>G | p.Asn422Ser | ENSBTAT00000052977.1 | Deleterious (0.01) |
| ZNF140 | 17 | 45116223 | C>T | 0/1 | c.1291C>T | p.Leu431Phe | ENSBTAT00000052977.1 | Deleterious (0.04) |
| ZNF140 | 17 | 45116235 | G>C | 0/1 | c.1X3G>C | p.Glu435Gln | ENSBTAT00000052977.1 | Tolerated (1) |
| ZNF140 | 17 | 45116250 | C>G | 0/1 | c.1318C>G | p.Leu440Val | ENSBTAT00000052977.1 | Tolerated (0.06) |
| EP400 | 17 | 46111281 | A>G | 0/1 | c.1598T>C | p.Val533Ala | ENSBTAT00000027216.5 | - |
| EP400 | 17 | 46111X2 | A>G | 0/1 | c.1577T>C | p.Val526Ala | ENSBTAT00000027216.5 | - |
| RNF10 | 17 | 65092167 | G>A | 0/1 | c.1471G>A | p.Val491Ile | ENSBTAT00000020937.5 | Tolerated (0.39) |
| RNF10 | 17 | 65092179 | A>G | 0/1 | c.1483A>G | p.Ser495Gly | ENSBTAT00000020937.5 | Tolerated (0.86) |
| RNF10 | 17 | 65092182 | G>T | 0/1 | c.1486G>T | p.Val496Phe | ENSBTAT00000020937.5 | Tolerated (0.5) |
| DRG1 | 17 | 72275600 | G>A | 0/1 | c.271G>A | p.Val91Met | ENSBTAT00000011834.5 | Tolerated (0.06) |
| DRG1 | 17 | 72275610 | A>G | 0/1 | c.281A>G | p.Tyr94Cys | ENSBTAT00000011834.5 | Deleterious (0.01) |
| DRG1 | 17 | 72275616 | T>A | 0/1 | c.287T>A | p.Phe96Tyr | ENSBTAT00000011834.5 | Deleterious (0.04) |
| ENSBTAG00000033890 | 17 | 7X34543 | G>C | 0/1 | c.851C>G | p.Ser284* | ENSBTAT00000050479.3 | - |
| ENSBTAG00000033890 | 17 | 7X34549 | G>T | 0/1 | c.845C>A | p.Ser282* | ENSBTAT00000050479.3 | - |
| ENSBTAG00000033890 | 17 | 7X34555 | A>T | 0/1 | c.839T>A | p.Leu280Gln | ENSBTAT00000050479.3 | Tolerated (0.23) |
| CDH13 | 18 | 10069100 | A>G | 0/1 | c.979A>G | p.Met327Val | ENSBTAT00000054165.2 | Tolerated (1) |
| MBTPS1 | 18 | 10426686 | G>T | 0/1 | c.242C>A | p.Ala81Glu | ENSBTAT00000013425.5 | Tolerated (0.96) |
| ATP2C2 | 18 | 10681005 | A>G | 0/1 | c.2177A>G | p.Asn726Ser | ENSBTAT00000061207.2 | Tolerated (0.26) |
| **Gene** | **BTA** | **Position** | **Base change** | **Genotype** | **cDNA** | **Protein** | **Transcript** | **SIFT** |
| N4BP1 | 18 | 17049920 | A>C | 0/1 | c.206T>G | p.Ile69Ser | ENSBTAT00000026560.5 | Deleterious (0) |
| N4BP1 | 18 | 170499X | T>C | 0/1 | c.196A>G | p.Ile66Val | ENSBTAT00000026560.5 | Tolerated (0.19) |
| DYNC1LI2 | 18 | 34597715 | G>A | 0/1 | c.59C>T | p.Pro20Leu | ENSBTAT00000000581.4 | Tolerated low confidence(0.05) |
| TERB1 | 18 | 34603288 | T>C | 0/1 | c.2164A>G | p.Lys722Glu | ENSBTAT00000032367.2 | Tolerated (1) |
| CMTR2 | 18 | 39903257 | A>G | 0/1 | c.397A>G | p.Asn133Asp | ENSBTAT00000063376.1 | Tolerated (0.36) |
| RYR1 | 18 | 48538662 | C>T | 0/1 | c.4112C>T | p.Ala1371Val | ENSBTAT00000009228.5 | - |
| RYR1 | 18 | 48538668 | C>T | 0/1 | c.4118C>T | p.Ala1373Val | ENSBTAT00000009228.5 | - |
| SPTBN4 | 18 | 50096945 | A>T | 0/1 | c.1166A>T | p.Tyr389Phe | ENSBTAT00000007434.5 | Tolerated (0.15) |
| PINLYP | 18 | 52120425 | A>C | 0/1 | c.6A>C | p.Lys2Asn | ENSBTAT00000031940.4 | Deleterious (0.01) |
| ZNF180 | 18 | 52640192 | T>C | 0/1 | c.643A>G | p.Lys215Glu | ENSBTAT00000052471.1 | Tolerated (0.17) |
| ZNF180 | 18 | 52640195 | C>T | 0/1 | c.640G>A | p.Glu214Lys | ENSBTAT00000052471.1 | Tolerated (0.78) |
| ZNF180 | 18 | 52640197 | G>C | 0/1 | c.638C>G | p.Thr213Ser | ENSBTAT00000052471.1 | Tolerated (1) |
| ZNF180 | 18 | 52640203 | G>T | 0/1 | c.632C>A | p.Thr211Asn | ENSBTAT00000052471.1 | Tolerated (0.71) |
| ZNF180 | 18 | 52640204 | T>C | 0/1 | c.631A>G | p.Thr211Ala | ENSBTAT00000052471.1 | Tolerated (0.74) |
| ZNF180 | 18 | 52640213 | G>C | 0/1 | c.622C>G | p.Gln208Glu | ENSBTAT00000052471.1 | Tolerated (0.35) |
| ZNF180 | 18 | 52640218 | G>C | 0/1 | c.617C>G | p.Thr206Ser | ENSBTAT00000052471.1 | Tolerated (1) |
| ZNF180 | 18 | 52640226 | C>A | 0/1 | c.609G>T | p.Met203Ile | ENSBTAT00000052471.1 | Tolerated (0.3) |
| PPP1R15A | 18 | 55928947 | A>C | 0/1 | c.1740A>C | p.Arg580Ser | ENSBTAT00000001702.4 | Deleterious (0) |
| RCN3 | 18 | 56427588 | G>A | 0/1 | c.641G>A | p.Arg214Lys | ENSBTAT00000031877.2 | - |
| **Gene** | **BTA** | **Position** | **Base change** | **Genotype** | **cDNA** | **Protein** | **Transcript** | **SIFT** |
| ENSBTAG00000038526 | 18 | 57750182 | G>A | 0/1 | c.716G>A | p.Arg239His | ENSBTAT00000057440.2 | Tolerated (1) |
| ENSBTAG00000045880 | 18 | 57938918 | AC>A | 0/1 | c.1525delG | p.Val509fs | ENSBTAT00000066015.1 | Tolerated (1) |
| ENSBTAG00000004925 | 18 | 60X4278 | A>G | 0/1 | c.2117A>G | p.Asn706Ser | ENSBTAT00000054007.2 | Tolerated (0.44) |
| ENSBTAG000000009X | 18 | 63226493 | A>G | 0/1 | c.184A>G | p.Lys62Glu | ENSBTAT00000001231.5 | Deleterious (0) |
| ENSBTAG00000046101 | 18 | 64015225 | GGCCGCACTCCCCGCACGCGTACGGCTTCTCGCCGGTGTGCGTGCGCCGGTGCACCACCAAGTGCATGCTCTGGCTGAAGGCCTT>G | 1/1 | c.1519 1602delAAGGCCTTCAGCCAGAGCATGCACTTGGTGGTGCACCGGCGCACGCACACCGGCGAGAAGCCGTACGCGTGCGGGGAGTGCGGC | p.Lys507 Gly534del | ENSBTAT00000064558.1 | - |
| GDPD1 | 19 | 10460208 | G>T | 0/1 | c.*58+1G>T |  | ENSBTAT00000048450.2 | - |
| SYNRG | 19 | 14176509 | A>T | 0/1 | c.939T>A | p.Ser313Arg | ENSBTAT00000013601.4 | Tolerated (0.72) |
| SYNRG | 19 | 14176510 | C>T | 0/1 | c.938G>A | p.Ser313Asn | ENSBTAT00000013601.4 | Tolerated (0.61) |
| SYNRG | 19 | 14176516 | A>G | 0/1 | c.932T>C | p.Met311Thr | ENSBTAT00000013601.4 | Tolerated (1) |
| RNF135 | 19 | 18331751 | AG>A | 0/1 | c.965delC | p.Ala322fs | ENSBTAT00000022890.5 | - |
| OMG | 19 | 19046133 | T>G | 0/1 | c.1011T>G | p.Asp337Glu | ENSBTAT00000035344.2 | Tolerated low confidence(0.49) |
| OMG | 19 | 19046141 | C>T | 0/1 | c.1019C>T | p.Thr340Ile | ENSBTAT00000035344.2 | Deleterious low confidence(0.05) |
| OMG | 19 | 19046159 | G>A | 0/1 | c.1037G>A | p.Arg346His | ENSBTAT00000035344.2 | Tolerated low confidence(1) |
| NSRP1 | 19 | 21843785 | G>A | 0/1 | c.1261G>A | p.Val421Met | ENSBTAT00000025760.5 | Tolerated (0.81) |
| **Gene** | **BTA** | **Position** | **Base change** | **Genotype** | **cDNA** | **Protein** | **Transcript** | **SIFT** |
| NSRP1 | 19 | 21843839 | A>C | 0/1 | c.1315A>C | p.Lys439Gln | ENSBTAT00000025760.5 | Tolerated (0.14) |
| NSRP1 | 19 | 21843843 | G>A | 0/1 | c.1319G>A | p.Arg440Gln | ENSBTAT00000025760.5 | Tolerated low confidence(0.63) |
| NSRP1 | 19 | 21843848 | G>A | 0/1 | c.1324G>A | p.Val442Ile | ENSBTAT00000025760.5 | Tolerated low confidence(0.36) |
| NSRP1 | 19 | 21843852 | G>A | 0/1 | c.1328G>A | p.Ser443Asn | ENSBTAT00000025760.5 | Tolerated low confidence(0.08) |
| NSRP1 | 19 | 21843857 | G>C | 0/1 | c.1333G>C | p.Glu445Gln | ENSBTAT00000025760.5 | Tolerated (1) |
| NSRP1 | 19 | 21843867 | A>G | 0/1 | c.1343A>G | p.Glu448Gly | ENSBTAT00000025760.5 | Deleterious low confidence(0.01) |
| CHRNB1 | 19 | 27764421 | T>C | 0/1 | c.1267T>C | p.Phe423Leu | ENSBTAT00000025624.4 | Tolerated (0.7) |
| CHRNB1 | 19 | 27764424 | A>G | 0/1 | c.1270A>G | p.Ile424Val | ENSBTAT00000025624.4 | Tolerated (0.32) |
| CHRNB1 | 19 | 27764440 | G>C | 0/1 | c.1286G>C | p.Arg429Pro | ENSBTAT00000025624.4 | Tolerated (0.28) |
| EIF4A1 | 19 | 27920827 | G>T | 0/1 | c.1145G>T | p.Arg382Met | ENSBTAT00000000144.2 | Deleterious (0.01) |
| EIF4A1 | 19 | 27920836 | G>A | 0/1 | c.1154G>A | p.Arg385Gln | ENSBTAT00000000144.2 | Tolerated (0.08) |
| EIF4A1 | 19 | 27920844 | G>A | 0/1 | c.1162G>A | p.Glu388Lys | ENSBTAT00000000144.2 | Deleterious (0) |
| MYH8 | 19 | X033263 | T>C | 0/1 | c.5546A>G | p.Lys1849Arg | ENSBTAT00000061119.2 | Tolerated (1) |
| MYH8 | 19 | X033291 | T>C | 0/1 | c.5518A>G | p.Ile1840Val | ENSBTAT00000061119.2 | Tolerated (1) |
| ADPRM | 19 | X298814 | A>G | 0/1 | c.158A>G | p.Gln53Arg | ENSBTAT00000065793.1 | Tolerated (0.8) |
| ADPRM | 19 | X298837 | C>A | 0/1 | c.181C>A | p.Gln61Lys | ENSBTAT00000065793.1 | Tolerated (0.54) |
| ADPRM | 19 | X298838 | A>C | 0/1 | c.182A>C | p.Gln61Pro | ENSBTAT00000065793.1 | Tolerated (0.92) |
| **Gene** | **BTA** | **Position** | **Base change** | **Genotype** | **cDNA** | **Protein** | **Transcript** | **SIFT** |
| ADPRM | 19 | X298843 | C>G | 0/1 | c.187C>G | p.Arg63Gly | ENSBTAT00000065793.1 | Tolerated (1) |
| ADPRM | 19 | X298846 | A>C | 0/1 | c.190A>C | p.Ser64Arg | ENSBTAT00000065793.1 | Tolerated (0.15) |
| ADPRM | 19 | X298850 | C>T | 0/1 | c.194C>T | p.Pro65Leu | ENSBTAT00000065793.1 | Tolerated (0.1) |
| ADPRM | 19 | X298855 | C>T | 0/1 | c.199C>T | p.Arg67Cys | ENSBTAT00000065793.1 | Tolerated (0.22) |
| DNAH9 | 19 | 31086835 | T>C | 0/1 | c.6077T>C | p.Leu2026Ser | ENSBTAT00000006131.5 | Tolerated (0.21) |
| WFIKKN2 | 19 | 36539X3 | C>T | 0/1 | c.398G>A | p.Arg133Lys | ENSBTAT00000000973.4 | Tolerated (0.47) |
| ABCC3 | 19 | 36706787 | C>T | 0/1 | c.1321G>A | p.Val441Met | ENSBTAT00000026744.5 | Tolerated (0.14) |
| CDK12 | 19 | 40541205 | A>G | 0/1 | c.2071A>G | p.Ile691Val | ENSBTAT00000002005.5 | Tolerated (0.47) |
| CCR7 | 19 | 414X189 | C>T | 0/1 | c.817G>A | p.Val273Ile | ENSBTAT00000053921.1 | Tolerated (0.76) |
| KRT26 | 19 | 41618900 | TC>T | 1/1 | c.1294delG | p.Glu432fs | ENSBTAT00000043126.2 | - |
| STAT3 | 19 | 4X63784 | G>C | 0/1 | c.2043C>G | p.Asp681Glu | ENSBTAT00000028687.5 | Tolerated (1) |
| CCR10 | 19 | 43377356 | GGCC>G | 0/1 | c.1018 1020delGGC | p.Gly340del | ENSBTAT00000025420.3 | - |
| CNTD1 | 19 | 43478693 | C>T | 0/1 | c.1001C>T | p.Thr334Ile | ENSBTAT00000026529.5 | - |
| MEOX1 | 19 | 44188717 | GC>G | 0/1 | c.335delG | p.Gly112fs | ENSBTAT00000054676.1 | - |
| MEIOC | 19 | 45044819 | G>A | 0/1 | c.1375G>A | p.Val459Met | ENSBTAT00000042938.3 | - |
| MEIOC | 19 | 45044831 | G>T | 0/1 | c.1387G>T | p.Ala463Ser | ENSBTAT00000042938.3 | Tolerated (0.07) |
| MEIOC | 19 | 45044835 | A>G | 0/1 | c.1391A>G | p.Asn464Ser | ENSBTAT00000042938.3 | Tolerated (0.53) |
| MEIOC | 19 | 45044868 | A>G | 0/1 | c.1424A>G | p.Asn475Ser | ENSBTAT00000042938.3 | Tolerated (0.1) |
| MEIOC | 19 | 45044915 | T>C | 0/1 | c.1471T>C | p.Phe491Leu | ENSBTAT00000042938.3 | Tolerated (0.21) |
| **Gene** | **BTA** | **Position** | **Base change** | **Genotype** | **cDNA** | **Protein** | **Transcript** | **SIFT** |
| MEIOC | 19 | 45044916 | T>C | 0/1 | c.1472T>C | p.Phe491Ser | ENSBTAT00000042938.3 | Tolerated (1) |
| MRC2 | 19 | 47721801 | C>T | 0/1 | c.91C>T | p.Pro31Ser | ENSBTAT00000020897.5 | Tolerated (0.65) |
| ACE | 19 | 48461385 | T>G | 0/1 | c.2519T>G | p.Met840Arg | ENSBTAT00000061106.2 | Deleterious (0) |
| ACE | 19 | 48461387 | T>G | 0/1 | c.2521T>G | p.Phe841Val | ENSBTAT00000061106.2 | Deleterious (0.01) |
| SECTM1A | 19 | 50944964 | G>T | 0/1 | c.206C>A | p.Pro69His | ENSBTAT00000034714.4 | Tolerated (0.12) |
| BAHCC1 | 19 | 51912424 | C>G | 0/1 | c.6914G>C | p.Gly2X5Ala | ENSBTAT00000022317.4 | Tolerated low confidence(0.09) |
| DNAH11 | 19 | 54341295 | G>A | 0/1 | c.3100G>A | p.Ala1034Thr | ENSBTAT00000022637.5 | Tolerated (0.91) |
| DNAH11 | 19 | 54341X2 | T>C | 0/1 | c.3107T>C | p.Met1036Thr | ENSBTAT00000022637.5 | Tolerated (0.47) |
| DNAH11 | 19 | 54341316 | C>A | 0/1 | c.3121C>A | p.Pro1041Thr | ENSBTAT00000022637.5 | Tolerated (0.26) |
| DNAH11 | 19 | 54341319 | G>C | 0/1 | c.3124G>C | p.Val1042Leu | ENSBTAT00000022637.5 | Tolerated (0.73) |
| KCTD2 | 19 | 57019917 | CG>C | 0/1 | c.98delC | p.Pro33fs | ENSBTAT00000042553.3 | - |
| OTOP3 | 19 | 57100095 | G>A | 0/1 | c.11XC>T | p.Ala377Val | ENSBTAT00000049720.3 | Tolerated (0.61) |
| OTOP3 | 19 | 57100126 | C>T | 0/1 | c.1099G>A | p.Ala367Thr | ENSBTAT00000049720.3 | Tolerated (0.19) |
| OTOP3 | 19 | 57100134 | G>A | 0/1 | c.1091C>T | p.Ala364Val | ENSBTAT00000049720.3 | Tolerated (1) |
| OTOP3 | 19 | 57100141 | C>T | 0/1 | c.1084G>A | p.Val362Met | ENSBTAT00000049720.3 | Deleterious (0.01) |
| OTOP3 | 19 | 57100159 | C>G | 0/1 | c.1066G>C | p.Val356Leu | ENSBTAT00000049720.3 | Tolerated (0.91) |
| DNAI2 | 19 | 57767869 | A>C | 0/1 | c.939T>G | p.His313Gln | ENSBTAT00000022242.4 | Tolerated (0.88) |
| DNAI2 | 19 | 57767879 | T>C | 0/1 | c.929A>G | p.Lys310Arg | ENSBTAT00000022242.4 | Tolerated (0.51) |
| DNAI2 | 19 | 57767892 | T>G | 0/1 | c.916A>C | p.MetX6Leu | ENSBTAT00000022242.4 | Tolerated (1) |
| **Gene** | **BTA** | **Position** | **Base change** | **Genotype** | **cDNA** | **Protein** | **Transcript** | **SIFT** |
| SH3PXD2B | 20 | 4010514 | T>A | 0/1 | c.2606A>T | p.Gln869Leu | ENSBTAT00000011874.5 | Tolerated (1) |
| SH3PXD2B | 20 | 4010544 | T>C | 0/1 | c.2576A>G | p.Asn859Ser | ENSBTAT00000011874.5 | Tolerated (0.06) |
| ERGIC1 | 20 | 4576432 | C>T | 0/1 | c.229C>T | p.Leu77Phe | ENSBTAT00000021218.5 | Tolerated (0.48) |
| ANKRD55 | 20 | 2X40805 | G>T | 0/1 | c.1XG>T | p.Ala44Ser | ENSBTAT00000001325.5 | Tolerated (0.27) |
| CCNO | 20 | 23926727 | A>G | 0/1 | c.904A>G | p.MetX2Val | ENSBTAT00000066316.1 | Tolerated (1) |
| ROPN1L | 20 | 62924534 | T>C | 0/1 | c.193A>G | p.Met65Val | ENSBTAT00000036661.2 | Tolerated (1) |
| ENSBTAG00000039409 | 20 | 71729037 | AGGTGG>A | 1/1 | c.500 504delGTGGG | p.Gly167fs | ENSBTAT00000052090.2 | - |
| ENSBTAG00000039409 | 20 | 71729044 | CCTGAGGTGGCCCAGGGTCCAGGTGGCCCTGAGGTGGCCCAGGGTCCAGGTGGCCCTCAGGTGGCTCCAGGGTCCAGG>C | 1/1 | c.506 545delCTGAGGTGGCCCAGGGTCCAGGTGGCCCTGAGGTGGCCCAGGGTCCAGGTGGCCCTCAGGTGGCTCCAGGGTCCAGG | p.Pro169fs | ENSBTAT00000052090.2 | - |
| SLCO3A1 | 21 | 15205714 | T>C | 0/1 | c.2057A>G | p.Asn686Ser | ENSBTAT00000002169.4 | Tolerated (0.53) |
| SLCO3A1 | 21 | 15205927 | G>A | 0/1 | c.1844C>T | p.Ala615Val | ENSBTAT00000002169.4 | Tolerated (0.32) |
| POLG | 21 | 21201648 | T>A | 0/1 | c.2797A>T | p.Ile933Phe | ENSBTAT00000011993.4 | Tolerated (1) |
| ENSBTAG00000002612 | 21 | 55394139 | G>A | 0/1 | c.1333G>A | p.Val445Ile | ENSBTAT00000003379.5 | Tolerated (0.08) |
| ENSBTAG00000002612 | 21 | 55394159 | T>A | 0/1 | c.1353T>A | p.Ser451Arg | ENSBTAT00000003379.5 | Tolerated (0.06) |
| ENSBTAG00000002612 | 21 | 55394161 | A>G | 0/1 | c.1355A>G | p.Asn452Ser | ENSBTAT00000003379.5 | Tolerated (1) |
| ENSBTAG00000002612 | 21 | 55394164 | T>A | 0/1 | c.1358T>A | p.Ile453Lys | ENSBTAT00000003379.5 | Tolerated (1) |
| ENSBTAG00000002612 | 21 | 55394172 | A>G | 0/1 | c.1366A>G | p.Thr456Ala | ENSBTAT00000003379.5 | Tolerated (1) |
| **Gene** | **BTA** | **Position** | **Base change** | **Genotype** | **cDNA** | **Protein** | **Transcript** | **SIFT** |
| ENSBTAG00000002612 | 21 | 55394199 | A>C | 0/1 | c.1393A>C | p.Asn465His | ENSBTAT00000003379.5 | Tolerated (0.06) |
| MIS18BP1 | 21 | 55435896 | C>T | 0/1 | c.935G>A | p.Arg312Lys | ENSBTAT00000003380.4 | Tolerated (0.54) |
| MIS18BP1 | 21 | 55435899 | T>C | 0/1 | c.932A>G | p.Gln311Arg | ENSBTAT00000003380.4 | Tolerated (1) |
| MIS18BP1 | 21 | 55435909 | T>C | 0/1 | c.922A>G | p.ThrX8Ala | ENSBTAT00000003380.4 | Tolerated (0.45) |
| MIS18BP1 | 21 | 55435918 | A>AT | 0/1 | c.912 913insA | p.PheX5fs | ENSBTAT00000003380.4 | - |
| MIS18BP1 | 21 | 55435920 | TG>T | 0/1 | c.910delC | p.GlnX4fs | ENSBTAT00000003380.4 | - |
| MIS18BP1 | 21 | 55435926 | G>C | 0/1 | c.905C>G | p.ThrX2Ser | ENSBTAT00000003380.4 | Tolerated (0.97) |
| MIS18BP1 | 21 | 55435957 | T>A | 0/1 | c.874A>T | p.Met292Leu | ENSBTAT00000003380.4 | Tolerated (0.46) |
| MIS18BP1 | 21 | 55435965 | G>A | 0/1 | c.866C>T | p.Pro289Leu | ENSBTAT00000003380.4 | Tolerated (0.2) |
| MIS18BP1 | 21 | 55435969 | A>G | 0/1 | c.862T>C | p.Phe288Leu | ENSBTAT00000003380.4 | Tolerated (1) |
| MIS18BP1 | 21 | 55435983 | T>G | 0/1 | c.848A>C | p.His283Pro | ENSBTAT00000003380.4 | Tolerated (0.28) |
| MIS18BP1 | 21 | 55435985 | C>G | 0/1 | c.846G>C | p.Met282Ile | ENSBTAT00000003380.4 | Tolerated (0.77) |
| MIS18BP1 | 21 | 55435992 | A>T | 0/1 | c.839T>A | p.Ile280Lys | ENSBTAT00000003380.4 | Tolerated (1) |
| BTBD7 | 21 | 58455855 | T>C | 0/1 | c.3275A>G | p.His1092Arg | ENSBTAT00000063586.1 | Tolerated low confidence(0.46) |
| BTBD7 | 21 | 58455869 | A>ACTG | 0/1 | c.3260 3261insCAG | p.Asp1087 Glu1088insSer | ENSBTAT00000063586.1 | - |
| WARS | 21 | 66897013 | G>T | 0/1 | c.705C>A | p.Asn235Lys | ENSBTAT00000006139.2 | Tolerated (0.71) |
| ENSBTAG00000025595 | 22 | 7112051 | G>A | 0/1 | c.275G>A | p.Arg92His | ENSBTAT00000036093.4 | Tolerated (0.29) |
| DLEC1 | 22 | 11619353 | G>A | 0/1 | c.5146G>A | p.Val1716Met | ENSBTAT00000011298.5 | Tolerated (1) |
| **Gene** | **BTA** | **Position** | **Base change** | **Genotype** | **cDNA** | **Protein** | **Transcript** | **SIFT** |
| DLEC1 | 22 | 11619368 | C>G | 0/1 | c.5161C>G | p.Pro1721Ala | ENSBTAT00000011298.5 | Tolerated (0.38) |
| XIRP1 | 22 | 12553287 | C>G | 0/1 | c.2518G>C | p.Ala840Pro | ENSBTAT00000065632.1 | Tolerated (1) |
| XIRP1 | 22 | 12553352 | T>C | 0/1 | c.2453A>G | p.His818Arg | ENSBTAT00000065632.1 | Tolerated (0.34) |
| XIRP1 | 22 | 12553357 | G>C | 0/1 | c.2448C>G | p.Ser816Arg | ENSBTAT00000065632.1 | Tolerated (0.55) |
| XIRP1 | 22 | 12553359 | T>C | 0/1 | c.2446A>G | p.Ser816Gly | ENSBTAT00000065632.1 | Tolerated (0.41) |
| XIRP1 | 22 | 12553360 | G>C | 0/1 | c.2445C>G | p.Asp815Glu | ENSBTAT00000065632.1 | Tolerated (0.21) |
| XIRP1 | 22 | 12553361 | T>C | 0/1 | c.2444A>G | p.Asp815Gly | ENSBTAT00000065632.1 | Tolerated (1) |
| XIRP1 | 22 | 12553365 | G>C | 0/1 | c.2440C>G | p.Gln814Glu | ENSBTAT00000065632.1 | Tolerated (0.08) |
| XIRP1 | 22 | 12553374 | A>C | 0/1 | c.2431T>G | p.Cys811Gly | ENSBTAT00000065632.1 | Tolerated (1) |
| XIRP1 | 22 | 12555667 | GT>G | 0/1 | c.137delA | p.His46fs | ENSBTAT00000065632.1 | - |
| NKTR | 22 | 15394506 | T>C | 0/1 | c.761T>C | p.Ile254Thr | ENSBTAT000000X863.4 | Tolerated low confidence(0.82) |
| NKTR | 22 | 15394507 | A>G | 0/1 | c.762A>G | p.Ile254Met | ENSBTAT000000X863.4 | Tolerated low confidence(0.19) |
| POMGNT2 | 22 | 15707590 | G>C | 0/1 | c.1614G>C | p.Met538Ile | ENSBTAT00000000583.5 | Tolerated (1) |
| ZNF197 | 22 | 16477659 | C>A | 0/1 | c.698C>A | p.Ala233Glu | ENSBTAT00000055475.1 | Tolerated (1) |
| ZNF197 | 22 | 16477667 | A>G | 0/1 | c.706A>G | p.Ser236Gly | ENSBTAT00000055475.1 | Tolerated (0.78) |
| ZNF197 | 22 | 16477673 | G>A | 0/1 | c.712G>A | p.Val238Ile | ENSBTAT00000055475.1 | Tolerated (0.33) |
| EDEM1 | 22 | 21279364 | TA>T | 0/1 | c.1233delT | p.Cys411fs | ENSBTAT00000017152.5 | - |
| FRMD4B | 22 | 32366700 | A>G | 0/1 | c.169A>G | p.Thr57Ala | ENSBTAT00000010028.5 | Tolerated (0.52) |
| **Gene** | **BTA** | **Position** | **Base change** | **Genotype** | **cDNA** | **Protein** | **Transcript** | **SIFT** |
| DNAH1 | 22 | 49086415 | C>T | 0/1 | c.2623G>A | p.Val875Ile | ENSBTAT00000066095.1 | Tolerated (1) |
| RAD54L2 | 22 | 49735362 | T>C | 0/1 | c.2761A>G | p.Thr921Ala | ENSBTAT000000457X.3 | Tolerated (0.84) |
| RBM6 | 22 | 50887725 | C>A | 0/1 | c.763G>T | p.Ala255Ser | ENSBTAT00000008X0.5 | Tolerated (1) |
| RBM6 | 22 | 50887737 | T>C | 0/1 | c.751A>G | p.Asn251Asp | ENSBTAT00000008X0.5 | Tolerated (1) |
| EFCAB12 | 22 | 56980775 | C>G | 0/1 | c.1413C>G | p.Asp471Glu | ENSBTAT00000028840.4 | Tolerated (0.63) |
| CFAP100 | 22 | 61332528 | C>T | 0/1 | c.X7C>T | p.Arg103Cys | ENSBTAT00000025850.5 | Deleterious (0) |
| UBR2 | 23 | 16363756 | C>A | 0/1 | c.4672C>A | p.Leu1558Ile | ENSBTAT00000007833.4 | Tolerated (0.3) |
| UBR2 | 23 | 16363779 | G>T | 0/1 | c.4695G>T | p.Lys1565Asn | ENSBTAT00000007833.4 | Tolerated (0.5) |
| UBR2 | 23 | 16363787 | T>C | 0/1 | c.4703T>C | p.Met1568Thr | ENSBTAT00000007833.4 | Tolerated (0.19) |
| UBR2 | 23 | 16363788 | G>A | 0/1 | c.4704G>A | p.Met1568Ile | ENSBTAT00000007833.4 | Tolerated (0.61) |
| GLTSCR1L | 23 | 16509115 | G>C | 0/1 | c.2554G>C | p.Val852Leu | ENSBTAT00000019726.2 | Tolerated low confidence(0.68) |
| GLTSCR1L | 23 | 16509120 | T>G | 0/1 | c.2559T>G | p.His853Gln | ENSBTAT00000019726.2 | Tolerated low confidence(0.38) |
| PEX6 | 23 | 16584857 | T>C | 0/1 | c.2432A>G | p.Asn811Ser | ENSBTAT00000007275.5 | Tolerated (1) |
| ICK | 23 | 24995024 | T>C | 0/1 | c.695A>G | p.Asn232Ser | ENSBTAT00000020711.5 | Tolerated (1) |
| ENSBTAG00000038397 | 23 | 25568610 | A>AGT | 0/1 | c.134 135dupTG | p.Gly46fs | ENSBTAT00000022181.4 | Deleterious (0.02) |
| ENSBTAG00000034945 | 23 | 25639404 | T>C | 0/1 | c.683A>G | p.Glu228Gly | ENSBTAT00000049419.2 | Deleterious (0.03) |
| ENSBTAG00000034945 | 23 | 25639439 | C>T | 0/1 | c.648G>A | p.Met216Ile | ENSBTAT00000049419.2 | Tolerated (1) |
| ENSBTAG00000034945 | 23 | 25639441 | T>C | 0/1 | c.646A>G | p.Met216Val | ENSBTAT00000049419.2 | Tolerated (0.58) |
| **Gene** | **BTA** | **Position** | **Base change** | **Genotype** | **cDNA** | **Protein** | **Transcript** | **SIFT** |
| ENSBTAG00000034945 | 23 | 25639444 | G>C | 0/1 | c.643C>G | p.Gln215Glu | ENSBTAT00000049419.2 | Tolerated (1) |
| ENSBTAG00000034945 | 23 | 25639449 | G>T | 0/1 | c.638C>A | p.Thr213Lys | ENSBTAT00000049419.2 | Tolerated (1) |
| ENSBTAG00000034945 | 23 | 25639525 | C>G | 0/1 | c.562G>C | p.Val188Leu | ENSBTAT00000049419.2 | - |
| SLC44A4 | 23 | 27XX37 | C>T | 0/1 | c.1328C>T | p.Ala443Val | ENSBTAT00000045367.3 | Deleterious (0) |
| SLC44A4 | 23 | 27XX46 | G>C | 0/1 | c.1337G>C | p.Gly446Ala | ENSBTAT00000045367.3 | Tolerated (0.09) |
| SLC44A4 | 23 | 27XX60 | A>G | 0/1 | c.1351A>G | p.Ile451Val | ENSBTAT00000045367.3 | Tolerated (1) |
| ENSBTAG00000031832 | 23 | 28992862 | GA>G | 0/1 | c.164delT | p.Leu55fs | ENSBTAT00000032794.4 | - |
| ENSBTAG00000037577 | 23 | 29622936 | A>G | 0/1 | c.661A>G | p.Ile221Val | ENSBTAT00000057288.2 | Tolerated (0.14) |
| ENSBTAG00000037577 | 23 | 29622997 | A>G | 0/1 | c.722A>G | p.Gln241Arg | ENSBTAT00000057288.2 | Tolerated (0.94) |
| ENSBTAG00000037577 | 23 | 29622998 | A>T | 0/1 | c.723A>T | p.Gln241His | ENSBTAT00000057288.2 | Tolerated (0.87) |
| GPLD1 | 23 | 3X26571 | T>G | 0/1 | c.1593T>G | p.Ile531Met | ENSBTAT00000006557.3 | Tolerated (0.12) |
| GPLD1 | 23 | 3X26613 | T>G | 0/1 | c.1635T>G | p.Ile545Met | ENSBTAT00000006557.3 | Tolerated (0.89) |
| GPLD1 | 23 | 3X26633 | G>C | 0/1 | c.1655G>C | p.Gly552Ala | ENSBTAT00000006557.3 | Tolerated (0.55) |
| GPLD1 | 23 | 3X26635 | T>C | 0/1 | c.1657T>C | p.Ser553Pro | ENSBTAT00000006557.3 | Tolerated (0.34) |
| GPLD1 | 23 | 3X26642 | A>G | 0/1 | c.1664A>G | p.Tyr555Cys | ENSBTAT00000006557.3 | Tolerated (0.16) |
| GPLD1 | 23 | 3X26643 | C>G | 0/1 | c.1665C>G | p.Tyr555* | ENSBTAT00000006557.3 | - |
| GPLD1 | 23 | 3X26648 | G>A | 0/1 | c.1670G>A | p.Ser557Asn | ENSBTAT00000006557.3 | Tolerated (0.67) |
| TSHZ1 | 24 | 3675313 | C>G | 0/1 | c.2734G>C | p.Ala912Pro | ENSBTAT00000000543.5 | Tolerated (0.47) |
| TMX3 | 24 | 8769076 | A>G | 0/1 | c.700A>G | p.Met234Val | ENSBTAT00000009957.5 | Tolerated (0.7) |
| **Gene** | **BTA** | **Position** | **Base change** | **Genotype** | **cDNA** | **Protein** | **Transcript** | **SIFT** |
| LAMA3 | 24 | 33204946 | T>C | 0/1 | c.3539A>G | p.His1180Arg | ENSBTAT00000060991.2 | Tolerated (0.22) |
| LAMA3 | 24 | 33204952 | G>A | 0/1 | c.3533C>T | p.Thr1178Ile | ENSBTAT00000060991.2 | Tolerated (0.51) |
| LAMA3 | 24 | 33204962 | C>T | 0/1 | c.3523G>A | p.Ala1175Thr | ENSBTAT00000060991.2 | Tolerated (0.35) |
| EPG5 | 24 | 46136599 | A>G | 0/1 | c.2545T>C | p.Ser849Pro | ENSBTAT00000016012.4 | Tolerated (0.39) |
| PRR35 | 25 | 495336 | G>T | 0/1 | c.1719G>T | p.Glu573Asp | ENSBTAT00000056123.2 | Deleterious low confidence(0.05) |
| TSC2 | 25 | 1600841 | A>G | 0/1 | c.374A>G | p.Lys125Arg | ENSBTAT00000049485.2 | Tolerated (0.51) |
| TSC2 | 25 | 1600844 | T>C | 0/1 | c.377T>C | p.Val126Ala | ENSBTAT00000049485.2 | Deleterious (0.04) |
| TSC2 | 25 | 1600861 | T>G | 0/1 | c.394T>G | p.Ser132Ala | ENSBTAT00000049485.2 | Tolerated (0.42) |
| CLUAP1 | 25 | 2898335 | C>A | 0/1 | c.173C>A | p.Thr58Asn | ENSBTAT00000052696.1 | Deleterious (0) |
| SEC14L5 | 25 | 4058706 | G>C | 0/1 | c.904G>C | p.GluX2Gln | ENSBTAT00000046931.3 | Tolerated (1) |
| ENSBTAG00000026383 | 25 | 4133651 | G>A | 0/1 | c.1429C>T | p.His477Tyr | ENSBTAT00000037483.4 | Tolerated low confidence(0.34) |
| GSPT1 | 25 | 10638509 | TTA>T | 0/1 | c.528 529delTA | p.Lys177fs | ENSBTAT00000004656.5 | - |
| ERCC4 | 25 | 12937155 | A>C | 0/1 | c.459A>C | p.Arg153Ser | ENSBTAT00000046336.3 | Deleterious (0) |
| ERCC4 | 25 | 12937258 | A>G | 0/1 | c.562A>G | p.Arg188Gly | ENSBTAT00000046336.3 | Tolerated (0.09) |
| DCUN1D3 | 25 | 18743228 | G>A | 0/1 | c.631C>T | p.Leu211Phe | ENSBTAT00000045756.2 | Deleterious (0) |
| DNAH3 | 25 | 19156363 | C>T | 0/1 | c.1151G>A | p.Arg384Gln | ENSBTAT00000024702.5 | Tolerated (0.62) |
| DNAH3 | 25 | 19165781 | A>T | 0/1 | c.674T>A | p.Val225Asp | ENSBTAT00000024702.5 | Tolerated (1) |
| DNAH3 | 25 | 19165782 | C>G | 0/1 | c.673G>C | p.Val225Leu | ENSBTAT00000024702.5 | Tolerated (0.61) |
| **Gene** | **BTA** | **Position** | **Base change** | **Genotype** | **cDNA** | **Protein** | **Transcript** | **SIFT** |
| DNAH3 | 25 | 19165791 | T>C | 0/1 | c.664A>G | p.Lys222Glu | ENSBTAT00000024702.5 | Tolerated (0.63) |
| DNAH3 | 25 | 19165792 | G>T | 0/1 | c.663C>A | p.Asp221Glu | ENSBTAT00000024702.5 | Tolerated (0.24) |
| UQCRC2 | 25 | 19864107 | G>T | 0/1 | c.427G>T | p.Ala143Ser | ENSBTAT00000028853.4 | Tolerated (0.07) |
| ENSBTAG00000013917 | 25 | 27105852 | C>A | 0/1 | c.4235C>A | p.Pro1412Gln | ENSBTAT00000018503.4 | - |
| ENSBTAG00000013917 | 25 | 27105884 | G>A | 0/1 | c.4267G>A | p.Val1423Met | ENSBTAT00000018503.4 | - |
| CALN1 | 25 | 28989569 | A>G | 0/1 | c.122A>G | p.Glu41Gly | ENSBTAT00000061255.1 | Deleterious (0.03) |
| STX1A | 25 | 34062152 | C>G | 0/1 | c.82C>G | p.Arg28Gly | ENSBTAT0000000X31.5 | Tolerated (0.53) |
| TBL2 | 25 | 34155732 | G>A | 0/1 | c.994G>A | p.Val332Met | ENSBTAT00000000422.5 | Tolerated (0.29) |
| TBL2 | 25 | 34155736 | C>T | 0/1 | c.998C>T | p.Pro333Leu | ENSBTAT00000000422.5 | Tolerated (0.14) |
| ZAN | 25 | 36377409 | ACCC>A | 0/1 | c.5039 5041delGGG | p.Gly1680del | ENSBTAT00000044014.2 | - |
| ZCWPW1 | 25 | 36661995 | G>A | 0/1 | c.637G>A | p.Asp213Asn | ENSBTAT00000001417.4 | Tolerated (0.52) |
| ZCWPW1 | 25 | 36661996 | A>C | 0/1 | c.638A>C | p.Asp213Ala | ENSBTAT00000001417.4 | Tolerated (1) |
| AIMP2 | 25 | 38583888 | G>A | 0/1 | c.676G>A | p.Asp226Asn | ENSBTAT00000009687.4 | Tolerated (0.49) |
| ELFN1 | 25 | 41819740 | C>T | 0/1 | c.628G>A | p.Ala210Thr | ENSBTAT00000043470.3 | Tolerated (0.64) |
| FFAR4 | 26 | 14917242 | A>T | 0/1 | c.226A>T | p.Ser76Cys | ENSBTAT00000000560.4 | Tolerated (1) |
| ZNF518A | 26 | 17372384 | G>A | 0/1 | c.4440G>A | p.Met1480Ile | ENSBTAT00000051963.2 | Tolerated (0.54) |
| PIK3AP1 | 26 | 17804831 | C>CG | 0/1 | c.1359 1360insC | p.Ala454fs | ENSBTAT00000026477.5 | - |
| PIK3AP1 | 26 | 17804837 | GA>G | 0/1 | c.1353delT | p.Phe453fs | ENSBTAT00000026477.5 | - |
| MMS19 | 26 | 18539726 | G>C | 0/1 | c.2843C>G | p.Ser948Cys | ENSBTAT00000061179.2 | Tolerated (0.13) |
| **Gene** | **BTA** | **Position** | **Base change** | **Genotype** | **cDNA** | **Protein** | **Transcript** | **SIFT** |
| MMS19 | 26 | 18539733 | G>A | 0/1 | c.2836C>T | p.Pro946Ser | ENSBTAT00000061179.2 | Tolerated (0.45) |
| DNMBP | 26 | 20697838 | CA>C | 0/1 | c.4359delT | p.Asp1453fs | ENSBTAT00000011772.4 | - |
| SH3PXD2A | 26 | 24414595 | T>G | 0/1 | c.1572A>C | p.Lys524Asn | ENSBTAT00000061357.2 | Deleterious low confidence(0.03) |
| COL17A1 | 26 | 24875888 | A>C | 0/1 | c.1008T>G | p.Asp336Glu | ENSBTAT00000013271.5 | - |
| DCLRE1A | 26 | 34574959 | T>C | 0/1 | c.2392A>G | p.Ile798Val | ENSBTAT00000026896.3 | - |
| DCLRE1A | 26 | 34574968 | T>C | 0/1 | c.2383A>G | p.Ile795Val | ENSBTAT00000026896.3 | Tolerated (1) |
| FAM160B1 | 26 | 35599524 | A>T | 0/1 | c.1332A>T | p.Glu444Asp | ENSBTAT00000003978.5 | Tolerated (0.89) |
| FAM160B1 | 26 | 35599550 | G>T | 0/1 | c.1358G>T | p.Arg453Met | ENSBTAT00000003978.5 | Tolerated (1) |
| EMX2 | 26 | 38164292 | AT>A | 0/1 | c.388delT | p.Tyr1Xfs | ENSBTAT00000003937.4 | Deleterious (0) |
| TENM3 | 27 | 12803761 | GA>G | 0/1 | c.6852delA | p.Glu2284fs | ENSBTAT00000061514.2 | - |
| ING2 | 27 | 13345496 | A>C | 0/1 | c.822A>C | p.Glu274Asp | ENSBTAT00000066213.1 | - |
| ING2 | 27 | 13345500 | A>G | 0/1 | c.826A>G | p.Thr276Ala | ENSBTAT00000066213.1 | Tolerated low confidence(0.48) |
| SLC25A4 | 27 | 14548572 | C>G | 0/1 | c.656C>G | p.Thr219Ser | ENSBTAT00000017580.2 | Tolerated low confidence(0.22) |
| MTUS1 | 27 | 18687266 | A>T | 0/1 | c.X28A>T | p.Thr1010Ser | ENSBTAT00000061385.2 | Tolerated (0.91) |
| KAT6A | 27 | 36537889 | TG>T | 0/1 | c.3138delC | p.Ile1047fs | ENSBTAT00000005242.5 | Tolerated (0.38) |
| PGBD5 | 28 | 1388953 | C>T | 0/1 | c.392G>A | p.Ser131Asn | ENSBTAT00000039008.4 | - |
| TARBP1 | 28 | 7268192 | G>T | 0/1 | c.1468C>A | p.Arg490Ser | ENSBTAT00000005936.5 | Tolerated (0.91) |
| TARBP1 | 28 | 7268198 | T>A | 0/1 | c.1462A>T | p.Met488Leu | ENSBTAT00000005936.5 | Tolerated (0.99) |
| **Gene** | **BTA** | **Position** | **Base change** | **Genotype** | **cDNA** | **Protein** | **Transcript** | **SIFT** |
| TARBP1 | 28 | 7268212 | C>T | 0/1 | c.1448G>A | p.Arg483Gln | ENSBTAT00000005936.5 | Tolerated (0.46) |
| TARBP1 | 28 | 7268277 | A>C | 0/1 | c.1383T>G | p.Asn461Lys | ENSBTAT00000005936.5 | Tolerated (0.36) |
| ZNF248 | 28 | 13245447 | T>A | 0/1 | c.1139T>A | p.Val380Glu | ENSBTAT00000025240.5 | Tolerated (1) |
|  |  |  |  |  |  |  |  |  |
| PHYHIPL | 28 | 14990173 | G>GACTATTCAAAA | 0/1 | c.478 478+1insACTATTCAAAA | - | ENSBTAT00000031362.4 | Tolerated (1) |
| PHYHIPL | 28 | 14990174 | G> GTTCATC | 0/1 | c.478+1 478+2insTTCATC | - | ENSBTAT00000031362.4 | - |
| ARID5B | 28 | 18036312 | G>A | 0/1 | c.362G>A | p.Cys121Tyr | ENSBTAT00000008847.5 | - |
| NRBF2 | 28 | 19380037 | T>G | 0/1 | c.279T>G | p.Asp93Glu | ENSBTAT00000052928.2 | Tolerated (1) |
| NRBF2 | 28 | 19380048 | A>C | 0/1 | c.290A>C | p.Gln97Pro | ENSBTAT00000052928.2 | Tolerated (0.78) |
| JMJD1C | 28 | 19437598 | T>A | 0/1 | c.1540A>T | p.Thr514Ser | ENSBTAT00000002067.5 | Tolerated (0.14) |
| JMJD1C | 28 | 19437679 | T>C | 0/1 | c.1459A>G | p.Met487Val | ENSBTAT00000002067.5 | Tolerated (1) |
| JMJD1C | 28 | 19437703 | G>A | 0/1 | c.1435C>T | p.Arg479Cys | ENSBTAT00000002067.5 | Tolerated (0.54) |
| WDFY4 | 28 | 43500729 | G>T | 0/1 | c.3679G>T | p.Ala1227Ser | ENSBTAT00000031292.4 | Tolerated low confidence(0.15) |
| FAT3 | 29 | 2079267 | C>A | 0/1 | c.7849G>T | p.Ala2617Ser | ENSBTAT00000005332.5 | Deleterious (0.03) |
| FAT3 | 29 | 2079272 | G>A | 0/1 | c.7844C>T | p.Ala2615Val | ENSBTAT00000005332.5 | Tolerated (0.21) |
| INTS4 | 29 | 18191662 | G>A | 0/1 | c.1065G>A | p.Trp355* | ENSBTAT00000061534.2 | Tolerated (0.16) |
| INTS4 | 29 | 18191667 | A>G | 0/1 | c.1070A>G | p.Asp357Gly | ENSBTAT00000061534.2 | Deleterious (0.05) |
| **Gene** | **BTA** | **Position** | **Base change** | **Genotype** | **cDNA** | **Protein** | **Transcript** | **SIFT** |
| UEVLD | 29 | 26417144 | C>G | 0/1 | c.411C>G | p.Asp137Glu | ENSBTAT00000018040.4 | Deleterious (0) |
| UEVLD | 29 | 26417154 | C>A | 0/1 | c.421C>A | p.Leu141Met | ENSBTAT00000018040.4 | Tolerated (1) |
| UEVLD | 29 | 26417158 | G>A | 0/1 | c.425G>A | p.Cys142Tyr | ENSBTAT00000018040.4 | Deleterious (0.04) |
| VWA5A | 29 | 27203340 | G>T | 0/1 | c.879G>T | p.Gln293His | ENSBTAT000000XX6.3 | Tolerated (1) |
| VWA5A | 29 | 27203341 | A>T | 0/1 | c.880A>T | p.Ser294Cys | ENSBTAT000000XX6.3 | Tolerated (0.19) |
| VWA5A | 29 | 27203359 | A>G | 0/1 | c.898A>G | p.SerX0Gly | ENSBTAT000000XX6.3 | Tolerated (1) |
| VWA5A | 29 | 27203360 | G>A | 0/1 | c.899G>A | p.SerX0Asn | ENSBTAT000000XX6.3 | Tolerated (0.38) |
| PANX3 | 29 | 28463189 | G>A | 0/1 | c.494G>A | p.Arg165Gln | ENSBTAT00000012385.3 | Tolerated (0.41) |
| PANX3 | 29 | 28463194 | G>A | 0/1 | c.499G>A | p.Glu167Lys | ENSBTAT00000012385.3 | Tolerated (1) |
| PANX3 | 29 | 28463206 | A>C | 0/1 | c.511A>C | p.Thr171Pro | ENSBTAT00000012385.3 | Tolerated (1) |
| PANX3 | 29 | 28463209 | G>C | 0/1 | c.514G>C | p.Asp172His | ENSBTAT00000012385.3 | Tolerated (0.16) |
| TIRAP | 29 | X016342 | A>T | 0/1 | c.611A>T | p.Tyr204Phe | ENSBTAT00000028660.3 | Tolerated (1) |
| TIRAP | 29 | X016373 | A>T | 0/1 | c.642A>T | p.Glu214Asp | ENSBTAT00000028660.3 | Tolerated (1) |
| TIRAP | 29 | X016384 | G>A | 0/1 | c.653G>A | p.Arg218His | ENSBTAT00000028660.3 | Tolerated (0.34) |
| KCNJ1 | 29 | 32757464 | AG>A | 0/1 | c.963delC | p.Phe322fs | ENSBTAT00000000008.4 | - |
| PTGDR2 | 29 | 37762043 | T>C | 0/1 | c.449A>G | p.Lys150Arg | ENSBTAT00000053928.2 | Tolerated (0.71) |
| PTGDR2 | 29 | 37762052 | G>A | 0/1 | c.440C>T | p.Ala147Val | ENSBTAT00000053928.2 | Tolerated (0.25) |
| PTGDR2 | 29 | 37762053 | C>T | 0/1 | c.439G>A | p.Ala147Thr | ENSBTAT00000053928.2 | Tolerated (0.29) |
| ENSBTAG00000048298 | 29 | 38X2703 | T>G | 0/1 | c.415A>C | p.Thr139Pro | ENSBTAT00000064140.1 | Deleterious (0) |
| **Gene** | **BTA** | **Position** | **Base change** | **Genotype** | **cDNA** | **Protein** | **Transcript** | **SIFT** |
| VWCE | 29 | 40497723 | C>T | 0/1 | c.644G>A | p.Arg215Gln | ENSBTAT00000028738.4 | Tolerated (0.12) |
| VWCE | 29 | 40497775 | T>C | 0/1 | c.592A>G | p.Ile198Val | ENSBTAT00000028738.4 | Tolerated (0.47) |
| FADS1 | 29 | 40941432 | A>G | 0/1 | c.1261T>C | p.Phe421Leu | ENSBTAT000000X146.4 | Tolerated (1) |
| FADS1 | 29 | 40941453 | T>C | 0/1 | c.1240A>G | p.Lys414Glu | ENSBTAT000000X146.4 | Tolerated (1) |
| AHNAK | 29 | 41577857 | T>C | 0/1 | c.15487A>G | p.Ile5163Val | ENSBTAT00000052103.2 | - |
| AHNAK | 29 | 41581645 | A>C | 0/1 | c.11699T>G | p.Leu3900Arg | ENSBTAT00000052103.2 | - |
| INTS5 | 29 | 41680453 | G>A | 0/1 | c.2677C>T | p.Arg893Cys | ENSBTAT00000013803.4 | Deleterious (0) |
| ENSBTAG00000010233 | 29 | 44099967 | T>A | 0/1 | c.955T>A | p.Phe319Ile | ENSBTAT00000013515.5 | Tolerated (0.79) |
| ENSBTAG00000010233 | 29 | 44099973 | G>A | 0/1 | c.961G>A | p.Ala321Thr | ENSBTAT00000013515.5 | Tolerated (0.61) |
| ENSBTAG00000010233 | 29 | 44099974 | C>G | 0/1 | c.962C>G | p.Ala321Gly | ENSBTAT00000013515.5 | Tolerated (0.41) |
| ENSBTAG00000010233 | 29 | 44099977 | G>T | 0/1 | c.965G>T | p.Gly322Val | ENSBTAT00000013515.5 | Tolerated (0.63) |
| ENSBTAG00000010233 | 29 | 44099994 | G>A | 0/1 | c.982G>A | p.Val328Ile | ENSBTAT00000013515.5 | Tolerated (0.65) |
| ENSBTAG00000010233 | 29 | 44100037 | G>T | 0/1 | c.1025G>T | p.Cys342Phe | ENSBTAT00000013515.5 | Tolerated (0.69) |
| LTBP3 | 29 | 44388233 | A>C | 0/1 | c.2347T>G | p.Cys783Gly | ENSBTAT00000008880.5 | Deleterious (0) |
| LTBP3 | 29 | 44388238 | T>G | 0/1 | c.2342A>C | p.Asp781Ala | ENSBTAT00000008880.5 | Deleterious (0.03) |
| KAT5 | 29 | 44546728 | CG>C | 0/1 | c.670delG | p.Asp224fs | ENSBTAT00000007328.5 | - |
| RAB1B | 29 | 44976471 | AACACCACTGCCAAGGTGAGCGGGCTAGGGCCAGGCCGCCGGGC>A | 0/1 | c.383 396+29delACACCACTGCCAAGGTGAGCGGGCTAGGGCCAGGCCGCCGGGC | p.Asn128fs | ENSBTAT00000064628.1 | - |
| **Gene** | **BTA** | **Position** | **Base change** | **Genotype** | **cDNA** | **Protein** | **Transcript** | **SIFT** |
| NUDT8 | 29 | 46124326 | A>G | 0/1 | c.353T>C | p.Leu118Pro | ENSBTAT00000044604.2 | Deleterious (0) |
| B4GALNT4 | 29 | 51296917 | CT>C | 0/1 | c.1016delA | p.Glu339fs | ENSBTAT00000034550.4 | - |
| CT47B1 | X | 4358389 | G>C | 0/1 | c.825G>C | p.Ter275Tyrext*? | ENSBTAT00000053470.2 | - |
| ATP11C | X | 23236085 | G>T | 0/1 | c.3297C>A | p.Ser1099Arg | ENSBTAT00000021914.4 | Tolerated (0.78) |
| CETN2 | X | 35386077 | T>C | 0/1 | c.40A>G | p.Thr14Ala | ENSBTAT00000010319.3 | Tolerated (0.7) |
| CETN2 | X | 35386080 | T>A | 0/1 | c.37A>T | p.Thr13Ser | ENSBTAT00000010319.3 | Tolerated (1) |
| CETN2 | X | 35386091 | T>C | 0/1 | c.26A>G | p.Asn9Ser | ENSBTAT00000010319.3 | Tolerated (1) |
| AVPR2 | X | 40037843 | G>GGAA | 0/1 | c.550 551insGAA | p.Asp184delinsGlyAsn | ENSBTAT00000065044.1 | - |
| AVPR2 | X | 40037874 | G>A | 0/1 | c.581G>A | p.Arg194His | ENSBTAT00000065044.1 | Tolerated (0.52) |
| HNRNPH2 | X | 55239877 | G>A | 0/1 | c.676G>A | p.Ala226Thr | ENSBTAT00000009864.4 | Tolerated (0.06) |
| HNRNPH2 | X | 55239900 | G>T | 0/1 | c.699G>T | p.Arg233Ser | ENSBTAT00000009864.4 | Tolerated (0.81) |
| COL4A5 | X | 61798261 | G>C | 0/1 | c.3495G>C | p.Lys1165Asn | ENSBTAT00000019400.5 | - |
| ACSL4 | X | 62508278 | T>A | 0/1 | c.47A>T | p.Gln16Leu | ENSBTAT00000054636.2 | Tolerated (0.29) |
| ACSL4 | X | 62508281 | T>C | 0/1 | c.44A>G | p.Lys15Arg | ENSBTAT00000054636.2 | Tolerated (0.37) |
| ACSL4 | X | 62508X8 | A>C | 0/1 | c.17T>G | p.Phe6Cys | ENSBTAT00000054636.2 | Tolerated low confidence(0.09) |
| GPR174 | X | 78187918 | GA>G | 0/1 | c.461delT | p.Leu154fs | ENSBTAT00000028584.4 | - |
| LPAR4 | X | 78629848 | G>A | 0/1 | c.52C>T | p.Leu18Phe | ENSBTAT00000004269.2 | Tolerated low confidence(0.06) |
| **Gene** | **BTA** | **Position** | **Base change** | **Genotype** | **cDNA** | **Protein** | **Transcript** | **SIFT** |
| LPAR4 | X | 78629850 | T>C | 0/1 | c.50A>G | p.Asn17Ser | ENSBTAT00000004269.2 | Tolerated low confidence(0.73) |
| ATP7A | X | 79448405 | G>T | 0/1 | c.1450C>A | p.Pro484Thr | ENSBTAT00000013214.4 | Tolerated (0.32) |
| ATP7A | X | 79448410 | T>A | 0/1 | c.1445A>T | p.Glu482Val | ENSBTAT00000013214.4 | Tolerated (0.18) |
| ATP7A | X | 79448411 | CCTT>C | 0/1 | c.1441 1443delAAG | p.Lys481del | ENSBTAT00000013214.4 | - |
| ATP7A | X | 79448444 | G>A | 0/1 | c.1411C>T | p.His471Tyr | ENSBTAT00000013214.4 | Tolerated (0.34) |
| **DGKK** | **X** | **93219478** | **T>C** | **0/1** | **c.2321A>G** | **p.Gln774Arg** | **ENSBTAT00000031198.4** | **Tolerated (0.05)** |
| NR0B1 | X | 118535364 | C>G | 0/1 | c.862C>G | p.Leu288Val | ENSBTAT00000020641.5 | Tolerated (1) |
| NR0B1 | X | 118535371 | G>A | 0/1 | c.869G>A | p.Ser290Asn | ENSBTAT00000020641.5 | Tolerated (0.25) |
| NR0B1 | X | 118535373 | T>A | 0/1 | c.871T>A | p.Cys291Ser | ENSBTAT00000020641.5 | Tolerated (0.18) |
| ZFY | X | 126164333 | C>T | 0/1 | c.601G>A | p.Gly201Ser | ENSBTAT00000010165.3 | Tolerated (1) |
| KLHL15 | X | 126287505 | A>G | 0/1 | c.466A>G | p.Thr156Ala | ENSBTAT00000027396.4 | Tolerated (0.63) |
| MGC134232 | X | 131212914 | A>G | 0/1 | c.406A>G | p.Asn136Asp | ENSBTAT00000054090.2 | Tolerated (0.12) |
| PHKA2 | X | 131764921 | G>T | 0/1 | c.832G>T | p.Val278Leu | ENSBTAT00000020422.5 | Tolerated (1) |
| ARHGAP6 | X | 137687448 | A>G | 0/1 | c.1357A>G | p.Met453Val | ENSBTAT00000003400.3 | Tolerated (0.35) |
