# Supplementary figures and images for: Clinical, cytogenetic and molecular genetic characterization of a tandem fusion translocation in a male Holstein cattle with congenital hypospadias and a ventricular septal defect
Source: PLoS One. 2020 Jan 10;15(1):e0227117. doi: 10.1371/journal.pone.0227117 (PMC6953810; doi:10.1371/journal.pone.0227117)

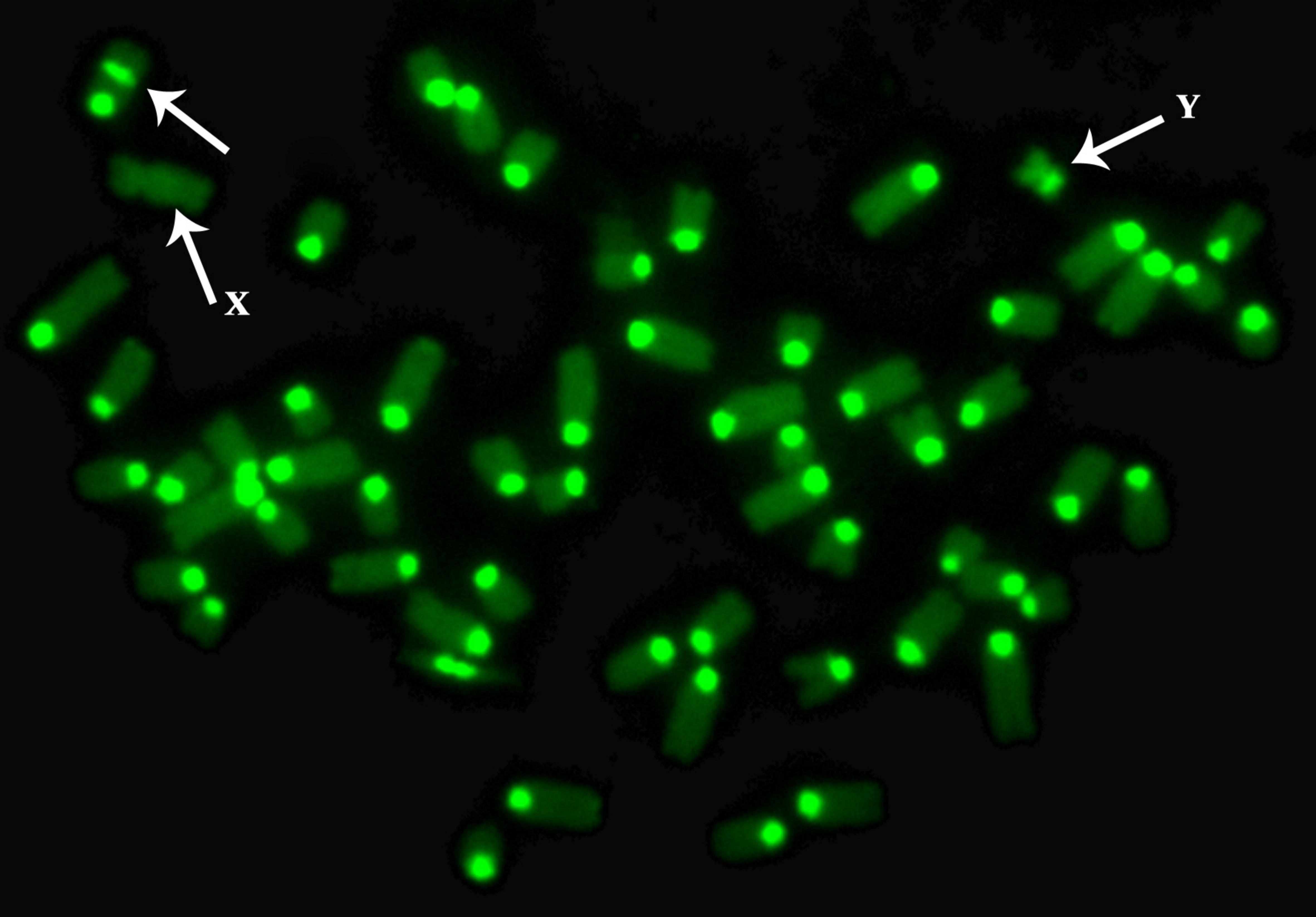

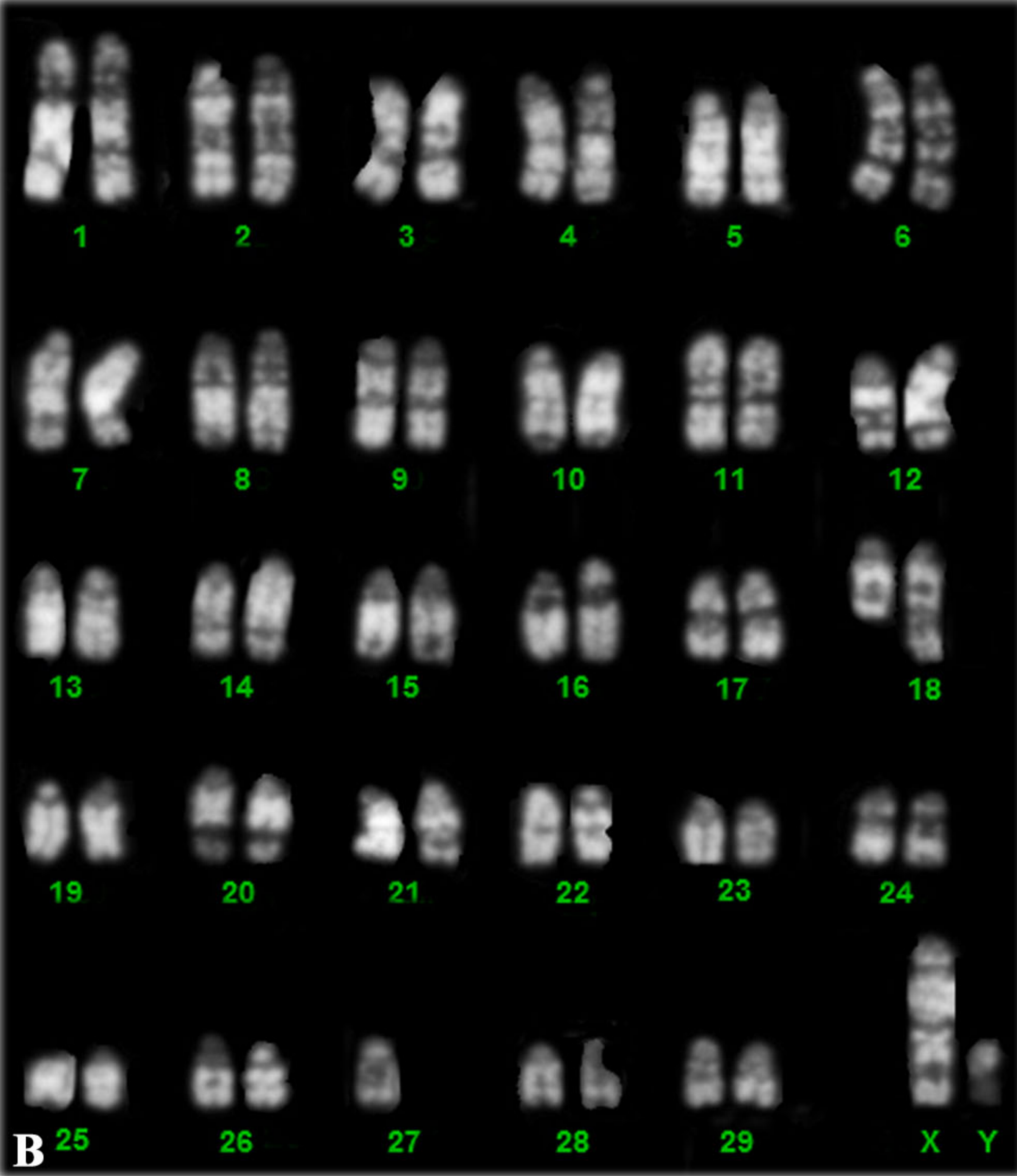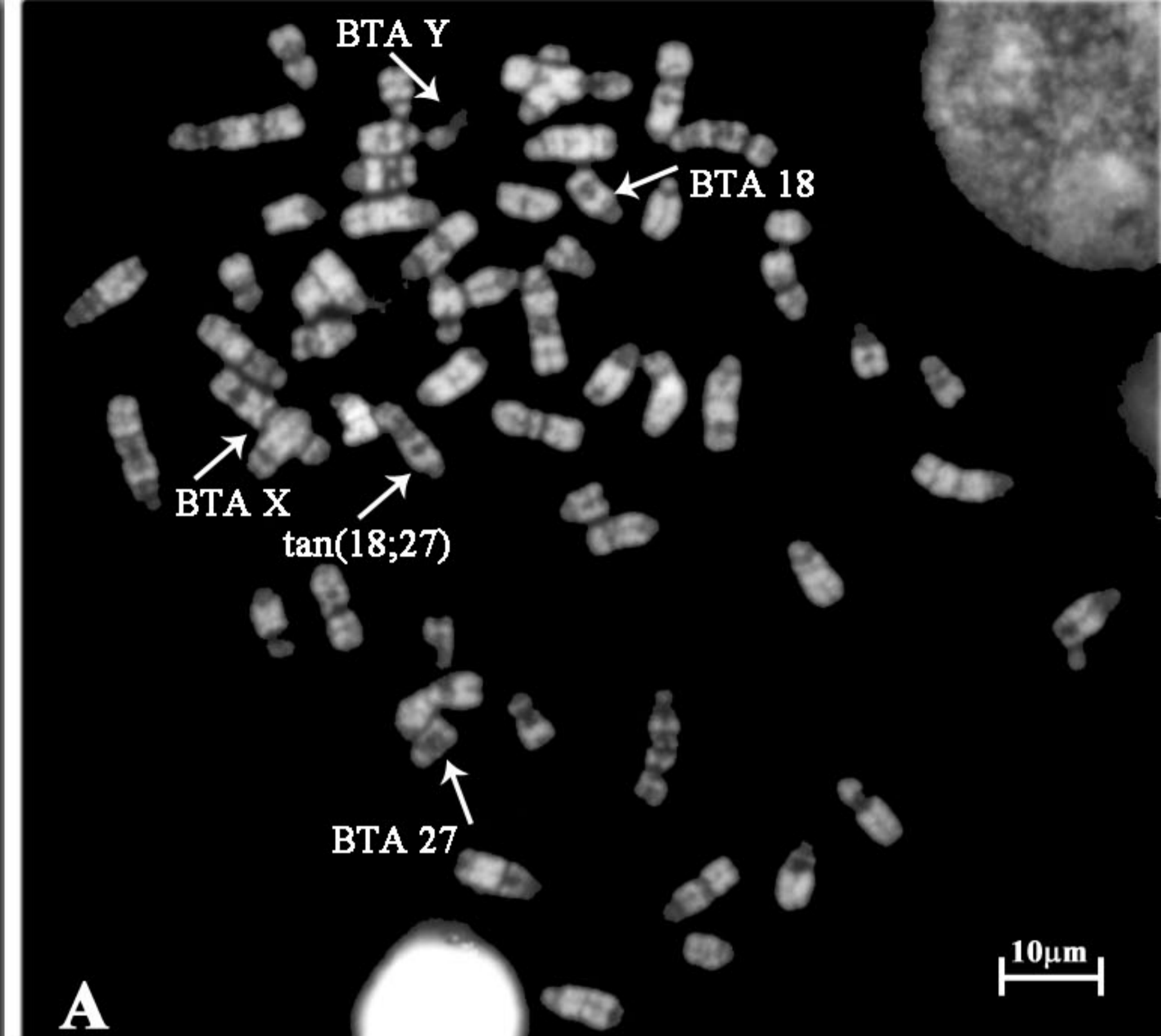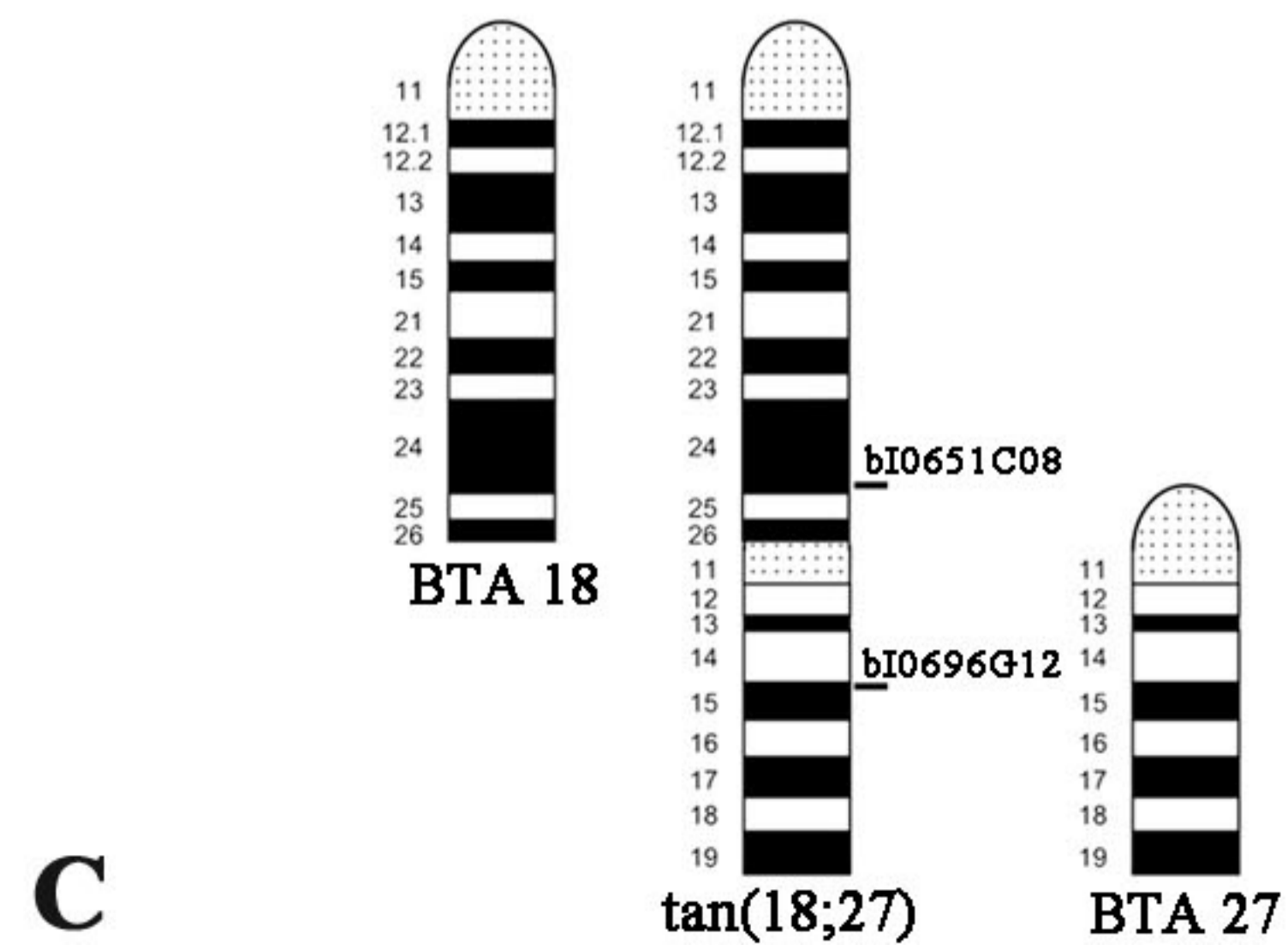

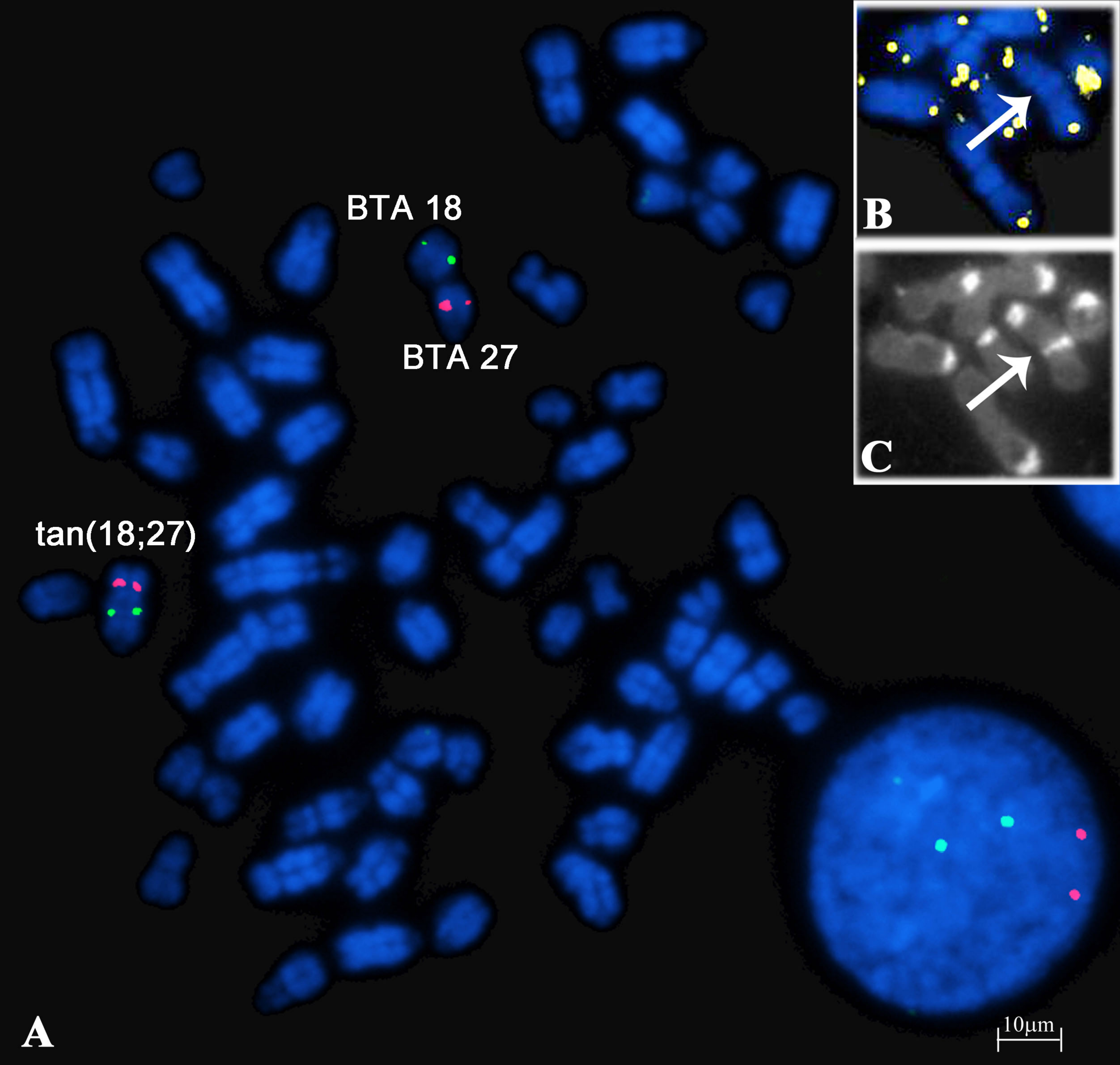

Supplement: S1 Raw Images — (PDF) [file pone.0227117.s003.pdf]
